# Supplementary material for: Cells recognize osmotic stress through liquid–liquid phase separation lubricated with poly(ADP-ribose)
Source: Nat Commun. 2021 Mar 1;12:1353. doi: 10.1038/s41467-021-21614-5 (PMC7921423; doi:10.1038/s41467-021-21614-5)
Supplement: Supplementary file 1 — Supplementary Information [file 41467_2021_21614_MOESM1_ESM.pdf]

Supplementary Information

Cells recognize osmotic stress through liquid–liquid phase separation lubricated with poly(ADP-ribose)

K. Watanabe, K. Morishita, X. Zhou, S. Shiizaki, Y. Uchiyama, M. Koike, I. Naguro, H. Ichijo.

Correspondence: kwatanabe@15.alumni.u-tokyo.ac.jp (K.W.), ichijo@mol.f.u-tokyo.ac.jp (H.I.)

This PDF file includes:

Supplementary Note
Supplementary Discussion
Supplementary Figures 1 to 8
Supplementary Methods

Other Supplementary Information files in this study include the following:

Supplementary Movies 1 to 4
Supplementary Data 1 to 2
Supplementary Software 1
Source Data

Supplementary Note

Full descriptions of the computational model in this study

To understand the essential principles of ASK3 condensation in a cell under hyperosmotic stress, we utilized and developed the previously reported simple computational model[19] with the effects of macromolecule crowding (Fig. 1c). According to the previous model, each single unit of self-associating ASK3 protein is regarded as an even square that occupies a lattice in a two-dimensional grid space corresponding to a single cell. When an ASK3 unit is adjacent to another, the ASK3 units have a binding relationship, which is independent of other binding pairs. Each ASK3 unit has no relationship between nonadjacent ASK3. A cluster of consecutive multiple ASK3 units is considered as a condensate in cells, which can be assumed to be a liquid-like or solid-like condensate depending on the system parameters mentioned below. At each time step, an ASK3 unit can move to an adjacent lattice.

Given the original position of an ASK3 unit, the ASK3 movement is categorized into three physicochemical actions: (1) diffusion, (2) exchange/vibration and (3) unbinding. If there are no neighboring ASK3 units before the movement (the destination 1 in Fig. 1c), the movement is considered simple diffusion with the rate constant k_1. If there is more than one neighboring ASK3 unit before the movement and if the destination position is already occupied by one of the neighbors (the destination 2 in Fig. 1c), the movement corresponds to an exchange between the ASK3 units in a cluster, namely, a rearrangement process within a liquid-like condensate. This exchange action is obeyed by the rate constant k_2. When k_2 is small, the action is considered vibration of ASK3 in a solid-like condensate. If there is more than one neighboring ASK3 units before the movement and if the destination position is occupied by none of the neighbors (the destination 3a–c in Fig. 1c), the movement can be accompanied by breakages of the binding relationships with the neighbors, i.e., the unbinding reaction. According to the simple Arrhenius equation, a rate constant of this overall unbinding movement k_3 is described as $k_3 = A \times \exp(-\Delta E \times n_{\mathrm{lost}} / \theta)$, where A is a frequency factor, ΔE is an activation energy in the unbinding reaction between a single pair of ASK3 units, n_{lost} is the number of neighboring ASK3 units whose binding relationship with the ASK3 unit to move will be lost by the movement, and θ is a temperature-like constant. Notably, the unbinding movement of ASK3 unit includes not only the dissociation process from the condensate but also the rearrangement process of the condensate (compare the destination 3a with the destinations 3b and 3c in Fig. 1c). Moreover, due to the penalty factor n_{lost}, the rearrangement that increases the surface of condensate (regarded as blue-colored positions in Fig. 1c for the clusters of 2 ASK3 units, for example) is usually less likely than the rearrangement that maintains (consider all potential patterns of the case when the destination 4b in Fig. 1c is occupied by not obstacle but ASK3 unit, for example). When $n_{\mathrm{lost}} = 0$ (the destination 3b in Fig. 1c), that is, $k_3 = A$, the unbinding movement is considered a shape-modified rearrangement process of the condensate with a void unbinding reaction.

In our model, obstacles were further added to reflect on the effects of macromolecular crowding in a cell. Each obstacle basically has the same properties as ASK3 unit; the obstacles takes the same size and shape as ASK3 unit, occupies a grid element and can move to an adjoining position. However, each obstacle has neither a binding relationship with ASK3 units nor one with the other obstacles, that is, each obstacle has neither a positive nor a negative effect on the other molecules. Hence, in addition to the above three physicochemical actions of an ASK3 unit, a new action arises: (4) reflection. If the destination position is occupied by an obstacle (the destination 4a and 4b in Fig. 1c), the ASK3 unit is not able to move to the

destination; therefore, the ASK3 unit is reflected by the obstacle and "moves to" the original position according to the rate constant k_4. Simultaneously, the movement of an obstacle is categorized into three physicochemical actions: (5) diffusion, (6) reflection by an ASK3 unit and (7) reflection by an obstacle. If the destination position is unoccupied (the destination 5 in Fig. 1c), the movement is simple diffusion of the obstacle according to the rate constant k_5. If the destination position is already occupied by an ASK3 unit or another obstacle (the destination 6 or 7 in Fig. 1c), the movement corresponds to reflection by the ASK3 unit or the other obstacle, and the obstacle "moves to" the original position depending on the rate constant k_6 or k_7, respectively.

We note that the ASK3 unit in our model may not necessarily correspond to a monomer of a single ASK3 peptide in the real world but homo-oligomer(s) of ASK3 or even hetero-oligomer(s). Likewise, the obstacle unit in our model is a virtual molecule that corresponds to the integration of subcellular molecules in the real world, such as macromolecules, small molecules and ions.

Supplementary Discussion

Technical descriptions of Supplementary Fig. 2a, b

We first utilized the previously reported simple computational model[19] to understand the driving force of ASK3 condensation in a cell under hyperosmotic stress. Hyperosmotic stress induces many effects on cells[1,7–9], but the first substantial trigger must be osmotically driven water efflux, followed by compulsive cell shrinkage. Therefore, we changed the grid space without any changes in the other parameters to mimic the initial cellular condition under hyperosmotic stress. We performed rejection kinetic Monte Carlo (rKMC) method with 10^6 iteration steps at each "cell size" ranging from 50 × 50 to 120 × 120 squares of the grid space. Our simulation results demonstrated that the decrease in the grid space progressively increased both the count and size of ASK3 clusters within the range of 120 × 120 to 85 × 85 squares. While continuously increasing the size, the further decrease from 85 × 85 to 50 × 50 squares reduced the count from the peak at 85 × 85. This phenomenon is explained by the result that the increase in the cluster size led to a decrease in the number of available free ASK3 units to seed new clusters and the result that the clusters easily fused with each other within the restricted grid space. However, the changing pattern was completely different from that of the cell-based experimental results (compare Supplementary Fig. 2b with Fig. 1b).

Technical descriptions of Fig. 1c–e

Although it is possible that the model is too simple to explain the phenomena in the real world, one of the biggest different assumptions in the model from cellular condition is the lack of macromolecular crowding[20]. In fact, ASK3 has a relatively high molecular mass of 147 kDa, and it is easy to speculate that the effects of macromolecular crowding on ASK3 are relatively large in cells. We therefore modified the model by adding obstacles to mimic macromolecular crowding (Fig. 1c). To maintain simplicity, the properties of obstacles were minimized to hold only an effect of size exclusion (details in Supplementary Note). Since the existence of obstacles prevented ASK3 unit movement and resulted in the slower convergence, we increased the number of iteration steps to 5×10^6 and executed rKMC method at each "cell size" ranging from 50 × 50 to 120 × 120 squares of the grid space (Fig. 1d, e, Supplementary Movie 1). Similar to Supplementary Fig. 2b, our simulation results indicated that the decrease in the grid space progressively increased both the number and size of ASK3 clusters, although the range was shifted from 120 × 120 to 65 × 65 squares (Fig. 1e). Nevertheless, while continuously increasing the count, the further decrease from 65 × 65 to 50 × 50 squares reduced the size from the peak at 65 × 65; this pattern of change was the same as that observed in the experimental results (Fig. 1b). Comparing Fig. 1e with Fig. 1b, we interpreted that the grid space from 65 × 65 to 50 × 50 (red shading in Fig. 1e) and the grid space of approximately 120 × 120 are homologous to the condition under hyperosmotic stress and isoosmotic state in cells, respectively. Indeed, this interpretation may be problematic: for example, (1) whereas the reduction of the grid space ranging from 120 × 120 to 65 × 65 squares gradually increased the count and size of clusters, hyperosmotic stress suddenly increased the count and size of condensates in cells (in other words, there was "dark matter" in the range between 120 × 120 and 65 × 65 squares); and (2) whereas there were small clusters even under isoosmotic conditions in silico, there were no condensates under isoosmotic conditions in cells. However, we should take into account the following three points. First, our model consists of the minimum elements and assumptions; hence, it is not surprising that the details are different. Second, in confocal microscopy

observations, we fixed the intensity of lasers and the sensitivity of detectors to acquire the overall appearance of ASK3 in cells from hypoosmotic to hyperosmotic stress. When raising the laser intensity and detector sensitivity, we were able to recognize much smaller condensates under hyperosmotic stress, although the intensity and size of larger condensates became too saturated and overestimated. Likewise, we might be able to detect smaller condensates even under isoosmotic conditions if the noise from ASK3 outside of the condensates could be eliminated. Finally, X-axis is plotted as osmolality in Fig. 1b and as the length of the grid space in Fig. 1e. When assuming a simple situation in which the Boyle–van't Hoff equation can be applied, the changes in cell volume are proportional to the inverse of the changes in osmolality. Hence, the interval of the X-axis variable is not the same between Fig. 1b and Fig. 1e. Altogether, the addition of obstacles enabled the model to properly represent the characteristics of ASK3 condensates in cells, which implies that macromolecular crowding is a critical driving force for hyperosmotic stress-induced ASK3 condensation in cells, although self-association of ASK3 is fundamental in the potential ability of ASK3 to form condensates.

Technical descriptions of Fig. 2c, d

By utilizing our computational model, we further performed in silico experiments to predict the changes in ASK3 condensates in cells after hyperosmotic stress is suddenly eliminated. We first iterated rKMC method with 5×10^6 steps at 55×55 squares of the grid space to grow up ASK3 clusters (as shown in Fig. 1d, e) and to determine the initial positions of ASK3 units and obstacles in subsequent simulations. We next expanded the grid space to 120×120 squares without any changes in the other parameters, including the molecules' positions, and began the simulation with 35×10^6 iteration steps (Fig. 2c, d, Supplementary Movie 3). The results demonstrated that the number of ASK3 clusters immediately and monotonously decreased as time progressed. Although the size of ASK3 clusters also decreased as time passed, it transiently increased just after the grid space was expanded to 120×120 squares. This interesting phenomenon can be interpreted as follows: obstacles gradually emerge from the original 55×55 squares after the grid space is extended, which is accompanied by the enormous decrease in the effects of size exclusion on ASK3 units. Free ASK3 units can therefore access the surface of clusters, which cause the clusters to grow. At the same time, ASK3 units can also emerge from the original grid space, which permits the dissociation of constitutive ASK3 units from the clusters and decreases the size of clusters. Hence, these two opposing behaviors compete with each other. Due to the proximity of the ASK3 clusters at the initial phase and the existence of the unbinding penalty, this interesting transition of power balance is observed.

Supplementary Figures

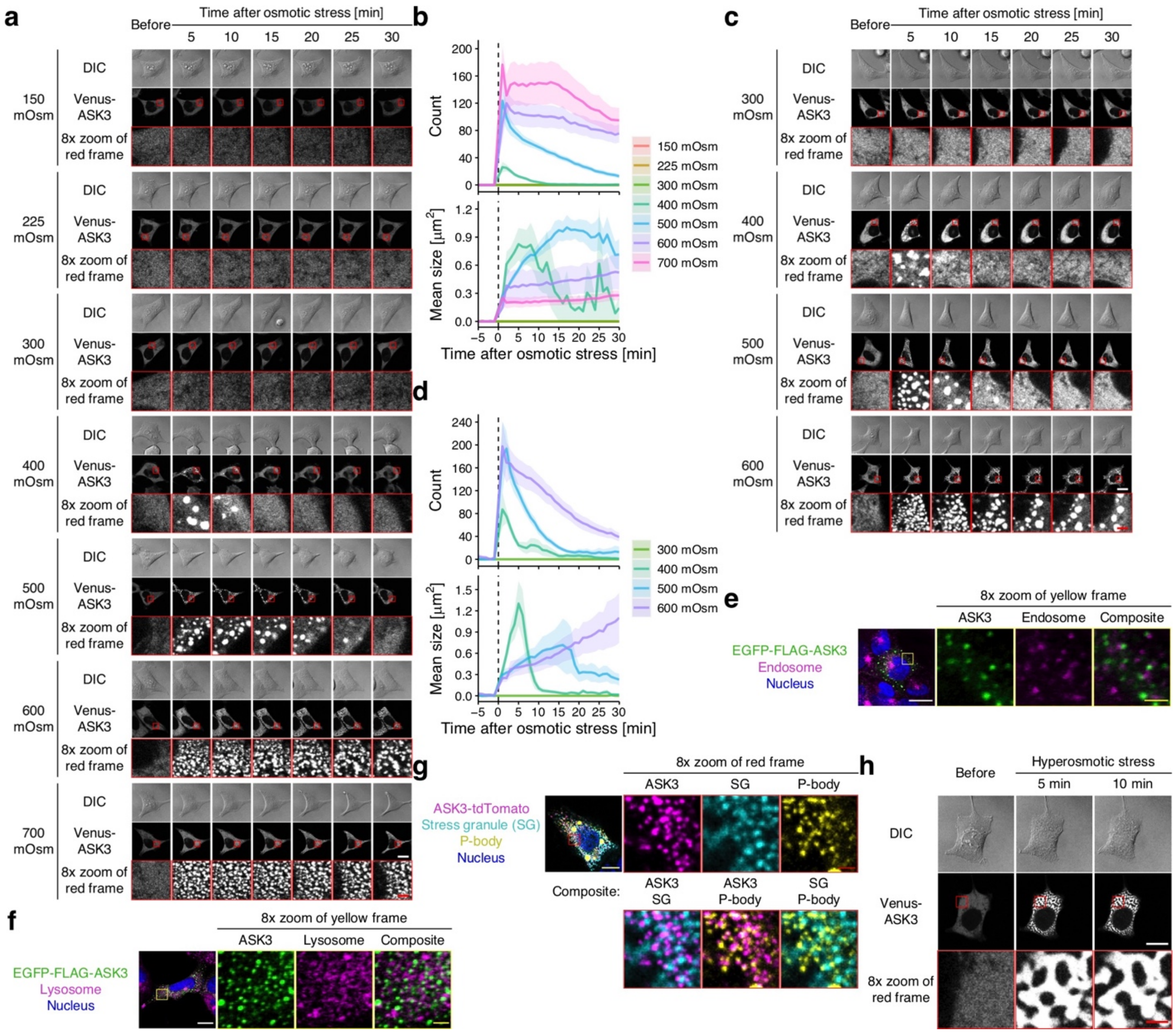

Supplementary Fig. 1 Characteristics of ASK3 condensates under hyperosmotic stress. a, **b** Time course of changes in the subcellular localization of ASK3 after osmotic stress in Venus-ASK3-stably expressing HEK293A (Venus-ASK3-HEK293A) cells. Hypoosmotic stress: ultrapure water-diluted medium, hyperosmotic stress: mannitol-supplemented medium, DIC: differential interference contrast, white bar: 20 μm, red bar: 2.5 μm. Data: mean ± SEM, n = 12 (700 mOsm), 14 (600 mOsm), 15 (400 mOsm), 16 (150 mOsm, 225 mOsm, 300 mOsm and 500 mOsm) cells pooled from 4 independent experiments. **c**, **d** Effects of sodium chloride on the subcellular localization of ASK3 in Venus-ASK3-HEK293A cells. Hyperosmotic stress: NaCl-supplemented medium, white bar: 20 μm, red bar: 2.5 μm. Data: mean ± SEM, n = 10 (400 mOsm, 500 mOsm and 600 mOsm), 12 (300 mOsm) cells pooled from 3 independent experiments. **e**, **f** Relationship between ASK3 condensates and early endosomes (e) or lysosomes (f). EGFP-FLAG-ASK3-transfected HEK293A cells were sampled after hyperosmotic stress (500 mOsm, 15 min). Endosome: immunofluorescence with an antibody against EEA1, lysosome: immunofluorescence with an antibody against LAMP1, white bar: 15 μm, yellow bar:

1.875 µm. A representative image set from 3 independent experiments is presented. **g** Relationship between ASK3 condensates and stress granules/P-bodies. Transfected HEK293A cells were sampled after hyperosmotic stress (800 mOsm, 45 min). ASK3: ASK3-tdTomato, stress granule (SG): HA-PABPC1 (immunofluorescence with an antibody against HA-tag), P-body: Venus-DCP1A, white bar: 10 µm, red bar: 1.25 µm. A representative image set from 3 independent experiments is presented. **h** An example of ASK3 condensates in spinodal decomposition-like pattern. Venus-ASK3-HEK293A cells were exposed to hyperosmotic stress (600 mOsm). White bar: 20 µm, red bar: 2.5 µm. A representative image set from 4 independent experiments is presented. Note that the DIC signal intensity cannot be compared among the images in a, c and h.

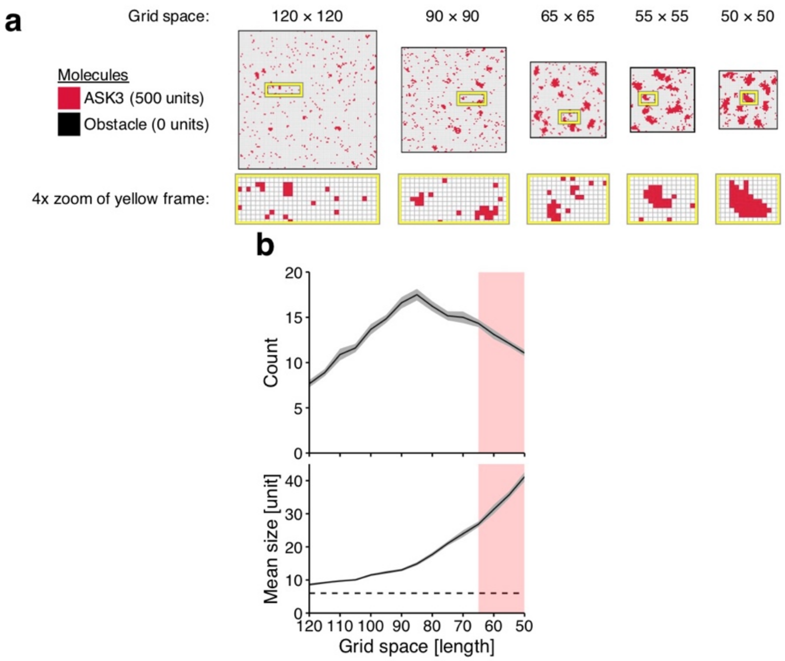

Supplementary Fig. 2 A computational model for protein diffusion and clustering in a two-dimensional grid space without obstacles. a, **b** A computational simulation of the relationship between the grid space and the number/size of ASK3 clusters using the previously reported model[19]. Results after 10^6 steps in the rejection kinetic Monte Carlo (rKMC) method at each grid space are presented. Red shading: range corresponding to red shading in Fig. 1e, dashed line: the minimum of ASK3 clusters definition. Data: mean ± SEM, n = 18 simulations.

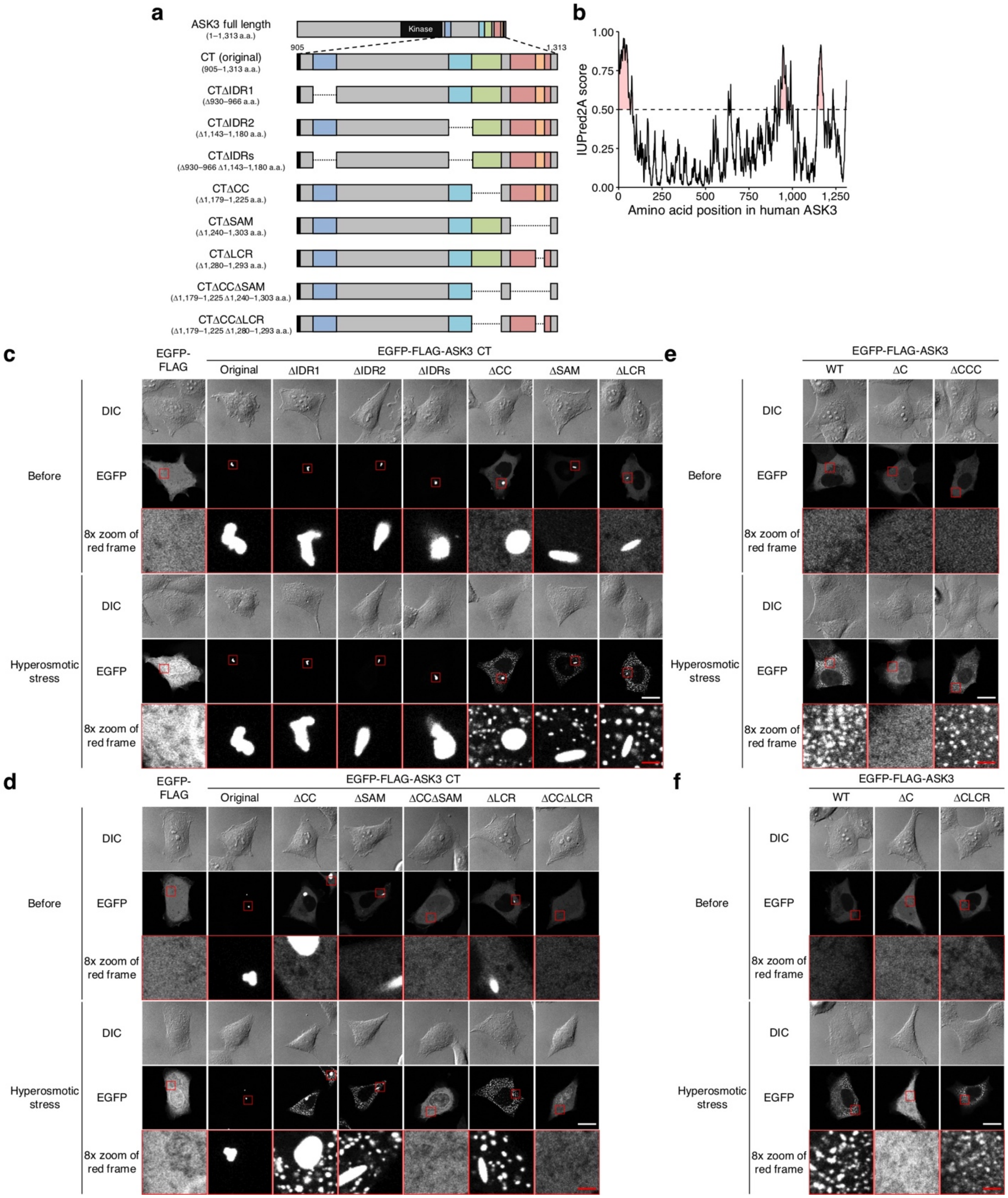

Supplementary Fig. 3 C-terminus coiled-coil domain and low complexity region are critical for the condensate formation of ASK3. a Schematic representation of ASK3 CT deletion mutants. The numbers indicate amino acid (a.a.) positions in the full-length wild-type (WT) ASK3. Black rectangle: kinase domain (652–908 a.a.), dark blue rectangle: C-terminus intrinsically disordered region 1 (CIDR1: 930–966 a.a.), light blue rectangle: CIDR2 (1,143–1,180 a.a.), green rectangle: C-terminus coiled-coil domain (CCC: 1,179–1,225 a.a.), red

rectangle: sterile alpha motif domain (SAM: 1,240–1,303 a.a.), orange rectangle: C-terminus low complexity region (CLCR: 1,280–1,293 a.a.). **b** Prediction of IDRs in ASK3 using the IUPred2A tool[67]. **c**, **d** Subcellular localization of ASK3 CT mutants in HEK293A cells. Hyperosmotic stress: 500 mOsm, 10 min. **e**, **f** Subcellular localization of the full-length ASK3 deletion mutants in HEK293A cells. Schematic representation of the mutants is presented in Fig. 3a. Hyperosmotic stress: 600 mOsm, 10 min. **c**–**f** DIC: differential interference contrast, white bar: 20 µm, red bar: 2.5 µm. A representative image set from 5 (c), 4 (d) or 3 (e and f) independent experiments is presented. Note that the DIC signal intensity cannot be compared among the images.

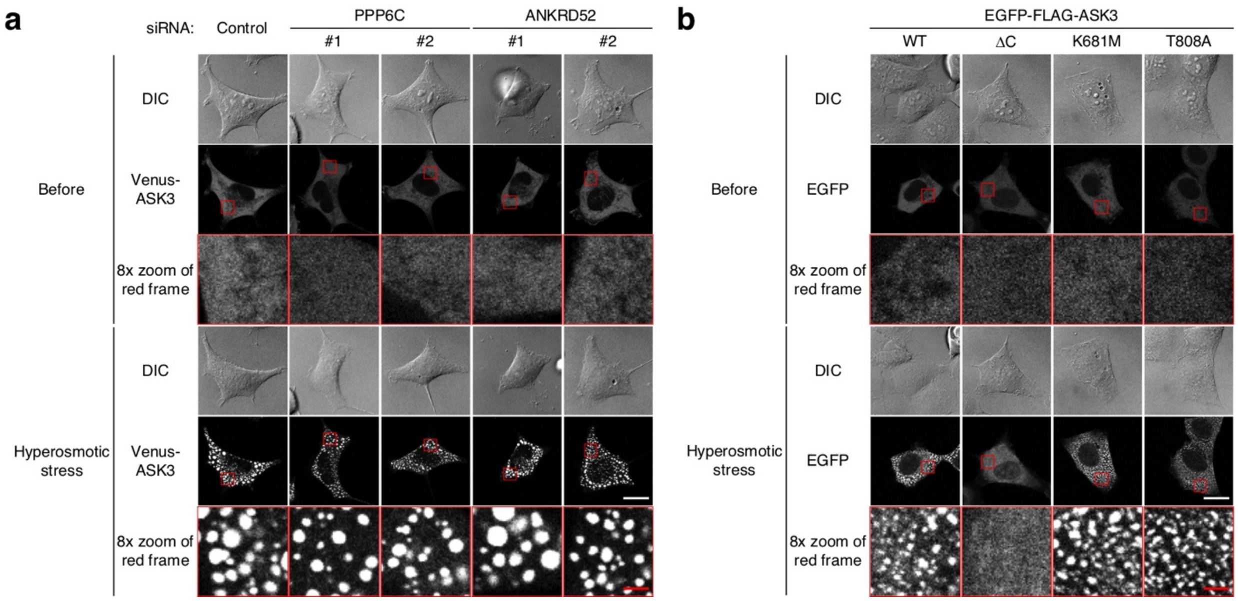

Supplementary Fig. 4 ASK3 inactivation is neither necessary nor sufficient for the condensate formation of ASK3. a Subcellular localization of ASK3 under the knockdown of PP6 in Venus-ASK3-stably expressing HEK293A (Venus-ASK3-HEK293A) cells. PPP6C: the catalytic subunit of PP6, ANKRD52: a PP6 subunit required for the substrate recognition[18,26]. Hyperosmotic stress: 500 mOsm, 10 min. **b** Subcellular localization of ASK3 mutants in HEK293A cells. WT: wild-type; K681M and T808A: kinase-inactive mutant[17]. Hyperosmotic stress: 600 mOsm, 10 min. **a**, **b** DIC: differential interference contrast, white bar: 20 µm, red bar: 2.5 µm. A representative image set from 3 (a) or 4 (b) independent experiments is presented. Note that the signal intensity of DIC cannot be compared among the images.

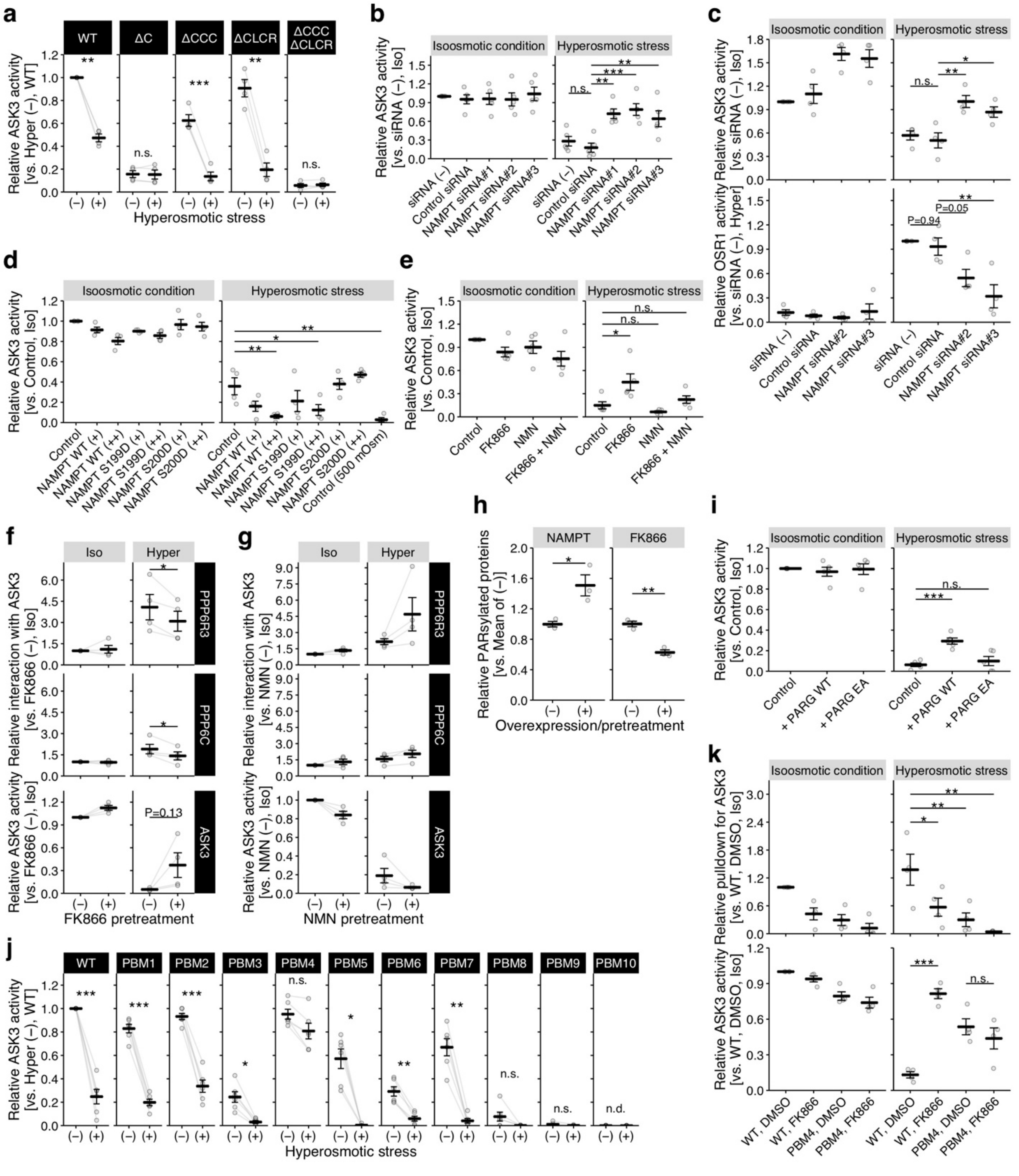

Supplementary Fig. 5 Quantified results of immunoblotting data in the main figures. a Quantification of 4 independent experiments corresponding to Fig. 3d. **b** Quantification of 5 independent experiments corresponding to Fig. 4c. **c** Quantification of 4 independent experiments corresponding to Fig. 4d. **d** Quantification of 4 independent experiments corresponding to Fig. 4e. **e** Quantification of 5 independent experiments corresponding to Fig. 4f. **f** Quantification of 4 independent experiments corresponding to Fig. 4g. **g** Quantification of 4 independent experiments corresponding to Fig. 4h. **h** Quantification of 3 independent

experiments corresponding to Fig. 5b. **i** Quantification of 5 independent experiments corresponding to Fig. 5c. **j** Quantification of 6 independent experiments corresponding to Fig. 6c. **k** Quantification of 4 independent experiments corresponding to Fig. 6d. **a**–**k** Individual values and the mean ± SEM are presented as gray points (connected with gray lines within the same experimental sets in a, f, g and j) and black lines, respectively. *$P < 0.05$, **$P < 0.01$, ***$P < 0.001$, n.s. (not significant) according to each statistical test (summarized in Supplementary Data 2), n.d. (not detected).

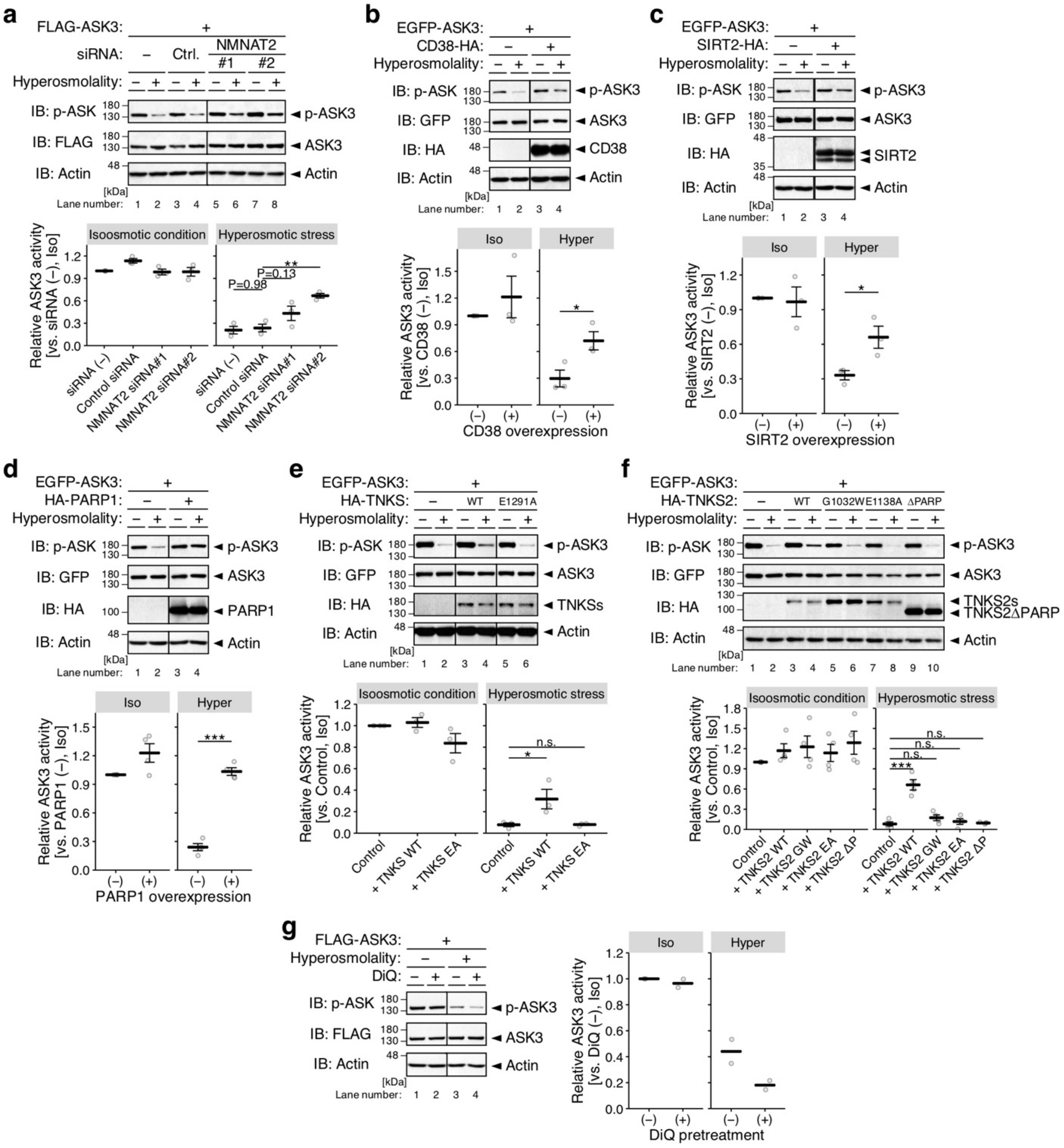

Supplementary Fig. 6 Major NAD-consuming enzymes downregulate ASK3 activity under hyperosmotic stress. **a** Effects of NMNAT2 depletion on ASK3 activity under hyperosmotic stress in FLAG-ASK3-stably expressing HEK293A (FLAG-ASK3-HEK293A) cells. **b**–**f** Effects of CD38 (b), SIRT2 (c), PARP1 (d), TNKS (e) or TNKS2 (f) overexpression on ASK3 activity under hyperosmotic stress in HEK293A cells. WT: wild-type; E1291A, G1032W and E1138A: PARP activity-inactive mutant; ΔPARP: catalytic domain-deleted mutant[65]. **g** Effects of DiQ pretreatment on ASK3 activity under hyperosmotic stress in FLAG-ASK3-HEK293A cells. DiQ (−): dimethyl sulfoxide (DMSO), solvent for DiQ; (+): 5 μM DiQ, a broad PARP inhibitor covering PARP1 and PARP2[66]; 30-min pretreatment. **a**–**g** The top or left panel is a representative image set of immunoblotting, and the bottom or right graph depicts the quantification of

independent experiments. Hyperosmolality (−): 300 mOsm; (+): 425 mOsm; 10 min. IB: immunoblotting. Note that superfluous lanes were digitally eliminated from blot images as indicated by vertical black lines. In the bottom and right graphs, individual values and the mean ± SEM are presented as gray points and black lines, respectively. $n = 3$ (a–c and e), 4 (d and f), 2 (g) independent experiments. *$P < 0.05$, **$P < 0.01$, ***$P < 0.001$, n.s. (not significant) according to each statistical test (summarized in Supplementary Data 2).

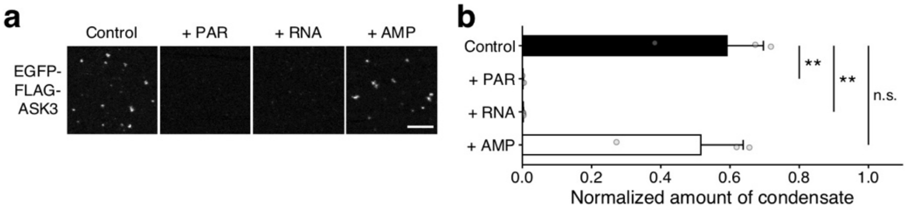

Supplementary Fig. 7 RNA inhibits the formation of solid-like ASK3 condensates. a, **b** Effects of RNA on solid-like ASK3 condensation in vitro. Control: 150 mM NaCl, 20 mM Tris (pH 7.5), 1 mM dithiothreitol (DTT), 20% polyethylene glycol (PEG), 15-min incubation on ice. PAR: 2.5 μM poly(ADP-ribose) (calculated as monomer unit), RNA: 2.5 μM poly(A) (calculated as monomer unit), AMP: 2.5 μM adenosine 5′-monophosphate, white bar: 5 μm. Data: mean ± SEM, $n = 3$ independent experiments. $^{**}P < 0.01$, n.s. (not significant) according to two-sided Dunnett's test. Of note, the chain length of PAR and poly(A) is 2–300 monomers and 2,100–10,000 monomers, respectively, according to manufacturers' datasheets, which would be more advantageous for RNA to interact with ASK3s multivalently and to melt the solid-like ASK3 condensates.

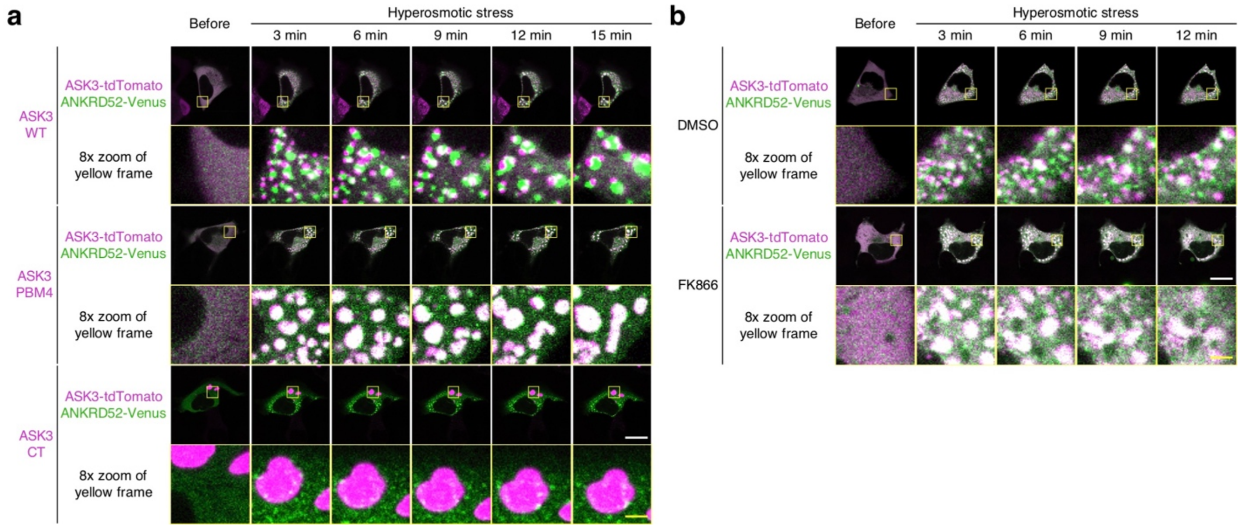

Supplementary Fig. 8 ASK3 condensates shares their phase boundary with PP6 condensates dependently on PAR. a Relationship between ANKRD52 and ASK3 PBM4 condensates in HEK293A cells. WT: wild-type, PBM4: PAR-unbinding mutant (Fig. 6a), CT: C-terminus fragment (Fig. 3a). **b** Effects of PAR depletion on the relationship between ANKRD52 and ASK3 condensates in HEK293A cells. DMSO: dimethyl sulfoxide (DMSO), solvent for FK866; FK866: 10 nM FK866; 18–24-hr pretreatment. **a**, **b** Magenta: ASK3-tdTomato, green: ANKRD52-Venus, hyperosmotic stress: 500 mOsm, white bar: 20 µm, yellow bar: 2.5 µm. A representative image set from 5 (a) or 4 (b) independent experiments is presented. Note that the signal intensity cannot be compared between samples.

Supplementary Methods

Reagents

FK866 (Cat. #F8557), β-nicotinamide mononucleotide (NMN; Cat. #N3501) and 1,5-dihydroxyisoquinoline (DiQ; Cat. #I138) were purchased from Sigma-Aldrich. FK866 and DiQ were dissolved at a final concentration of 1,000x in dimethyl sulfoxide (DMSO; Sigma-Aldrich, Cat. #D5879), and NMN were dissolved at a final concentration of 1,000x in ultrapure water. The solvents were used as each negative control.

Expression plasmids

Expression plasmids for this study were constructed by standard molecular biology techniques, and all constructs were verified by sequencing. Human ASK3 cDNA (cording sequence (CDS) of NM_001001671.3 with c.147C>T, c.574G>A) was previously cloned and subcloned into pcDNA3/GW (Invitrogen) with an N-terminal FLAG- or HA-tag[17] or into pcDNA4/TO (Invitrogen) with an N-terminal FLAG- or EGFP-tag[18]. Human ASK3 cDNA was also subcloned into pcDNA3 with a C-terminal tdTomato-tag (cDNA was gifted by M. Davidson, Florida State University, via Addgene: plasmid #54653) or pcDNA4/TO with an N-terminal Venus- or EGFP-FLAG-tag. EGFP-FLAG-tag cDNA was constructed by connecting EGFP-tag and FLAG-tag with a Gly-Gly linker and subcloned into pcDNA4/TO. cDNAs of ASK3 mutants ΔN (CDS of NM_001001671.3 with c.1_1,866del), ΔC (CDS of NM_001001671.3 with c.147C>T, c.574G>A, c.2,734_3,939del), NT (CDS of NM_001001671.3 with c.147C>T, c.574G>A, c.1,867_3,939del), KD (CDS of NM_001001671.3 with c.1_1,866del, c.2,734_3,939del), CT (CDS of NM_001001671.3 with c.1_2,712del), ΔCCC (CDS of NM_001001671.3 with c.147C>T, c.574G>A, c.3,535_3,675del), ΔCLCR (CDS of NM_001001671.3 with c.147C>T, c.574G>A, c.3,838_3,879del), ΔCCCΔCLCR (CDS of NM_001001671.3 with c.147C>T, c.574G>A, c.3,535_3,675del, c.3,838_3,879del), PBM1 (R58A/R59A; CDS of NM_001001671.3 with c.147C>T, c.172CGGCGG>GCCGCC, c.574G>A), PBM2 (R203A/R204A; CDS of NM_001001671.3 with c.147C>T, c.574G>A, c.607AGACGA>GCCGCC), PBM3 (R252A/K253A/R255A; CDS of NM_001001671.3 with c.147C>T, c.574G>A, c.754CGGAAA>GCCGCC, c.763AGA>GCC), PBM4 (R332A/R333A; CDS of NM_001001671.3 with c.147C>T, c.574G>A, c.994AGGAGA>GCCGCC), PBM5 (R391A/K392A; CDS of NM_001001671.3 with c.147C>T, c.574G>A, c.1,171CGCAAA>GCCGCC), PBM6 (R424A/K425A; CDS of NM_001001671.3 with c.147C>T, c.574G>A, c.1,270AGGAAA>GCCGCC), PBM7 (R436A/K437A; CDS of NM_001001671.3 with c.147C>T, c.574G>A, c.1,306AGAAAA>GCCGCC), PBM8 (R493A/R494A/K496A/K497A; CDS of NM_001001671.3 with c.147C>T, c.574G>A, c.1,477CGGCG>GCCGC, c.1,686AAGAAA>GCCGCC), PBM9 (K797A/R798A; CDS of NM_001001671.3 with c.147C>T, c.574G>A, c.2,389AAACGT>GCCGCC), PBM10 (K895A/R896A; CDS of NM_001001671.3 with c.147C>T, c.574G>A, c.2,683AAACGT>GCCGCC), CTΔIDR1 (CDS of NM_001001671.3 with c.1_2,712del, c.2,788_2,898del), CTΔIDR2 (CDS of NM_001001671.3 with c.1_2,712del, c.3,427_3,540del), CTΔIDRs (CDS of NM_001001671.3 with c.1_2,712del, c.2,788_2,898del, c.3,427_3,540del), CTΔCC (CDS of NM_001001671.3 with c.1_2,712del, c.3,535_3,675del), CTΔSAM (CDS of NM_001001671.3 with c.1_2,712del, c.3,718_3,909del), CTΔLCR (CDS of NM_001001671.3 with c.1_2,712del, c.3,838_3,879del), CTΔCCΔSAM (CDS of NM_001001671.3 with c.1_2,712del, c.3,535_3,675del, c.3,718_3,909del), CTΔCCΔLCR (CDS of NM_001001671.3

with c.1_2,712del, c.3,535_3,675del, c.3,838_3,879del) , K681M (CDS of NM_001001671.3 with c.147C>T, c.574G>A, c.2,042AA>TG), T808A (CDS of NM_001001671.3 with c.147C>T, c.574G>A, c.2,422A>G) were constructed from full-length ASK3 and subcloned into pcDNA4/TO with an N-terminal EGFP-FLAG-tag. CIDRs in ASK3 were predicted using the IUPred2A tool[67] (URL https://iupred2a.elte.hu/) (Supplementary Fig. 3b). PBMs in ASK3 were defined based on the central positively charged $[KR]_5$-$[KR]_6$ in the consensus sequence[45,46] (Fig. 6a). The ASK3 PBM4 mutant cDNA was also subcloned into pcDNA3/GW with an N-terminal HA-tag or pcDNA3 with a C-terminal tdTomato-tag. The ASK3 CT fragment cDNA was also subcloned into pcDNA3 with a C-terminal tdTomato-tag. Human ANKRD52 cDNA (CDS of NM_173595.3) was cloned previously[18] and subcloned into pcDNA3/GW with a C-terminal Venus-tag. Human NAMPT cDNA (CDS of NM_005746.2) was cloned from a cDNA pool derived from HEK293A cells and subcloned into pcDNA3/GW with an N-terminal FLAG-tag. cDNAs of NAMPT mutants S199D (CDS of NM_005746.2 with c.595TC>GA) and S200D (CDS of NM_005746.2 with c.598TC>GA)[29] were constructed from full-length NAMPT and subcloned into pcDNA3/GW with an N-terminal FLAG-tag. Human PPP6R3 (CDS of NM_001164161.1) with an N-terminal YFP-tag and human PPP6C (CDS of NM_002721.4) with a C-terminal HA-tag were constructed previously[18]. Human PARG cDNA (CDS of NM_001303486.1) and human SIRT2 cDNA (CDS of NM_030593.2) were cloned from a cDNA pool derived from HEK293A cells and subcloned into pcDNA3/GW with a C-terminal HA-tag. cDNA of PARG mutant E673A/E674A (CDS of NM_001303486.1 with c.2,018AAGAA>CCGCC)[41] was constructed from full-length PARG and subcloned into pcDNA3/GW with a C-terminal HA-tag. The wild-type PARG and PARG mutant cDNAs were also subcloned into pcDNA3/GW with a C-terminal Venus-tag. Human PABPC1 cDNA (CDS of NM_002568.4) and human DCP1A cDNA (CDS of NM_018403.7) were cloned from a cDNA pool derived from HEK293A cells and subcloned into pcDNA3/GW with an N-terminal HA- and Venus-tag, respectively. Human CD38 cDNA (CDS of NM_001775.3) was cloned from a cDNA pool derived from A594 cells and subcloned into pcDNA3/GW with a C-terminal HA-tag. Human PARP1 cDNA (CDS of NM_001618.3) and human TNKS2 cDNA (CDS of NM_025235.3 with c.2,361G>A) was cloned from a cDNA pool derived from HeLa cells and subcloned into pcDNA3/GW with an N-terminal HA-tag. cDNAs of TNKS2 mutants G1032W (CDS of NM_025235.3 with c.2,361G>A, c.3,094G>T), E1138A (CDS of NM_025235.3 with c.2,361G>A, c.3,413AA>CC) and ΔPARP (CDS of NM_025235.3 with c.2,361G>A, c.2,875_3,501del)[65] were constructed from full-length TNKS2 and subcloned into pcDNA3/GW with an N-terminal HA-tag. Human TNKS cDNA (CDS of NM_003747.2 with c.1,386A>G, c.3,447A>G) was kindly gifted from H. Seimiya (Japanese Foundation for Cancer Research) and subcloned into pcDNA3/GW with an N-terminal HA-tag. cDNA of TNKS mutant E1291A (CDS of NM_003747.2 with c.1,386A>G, c.3,447A>G, c.3,872A>C)[65] was constructed from full-length TNKS and subcloned into pcDNA3/GW with an N-terminal HA-tag. cDNA of WWE domain in human RNF146 (c.247–549 in CDS of NM_030963.3)[47,68] was cloned from a cDNA pool derived from HEK293A cells and subcloned into pcDNA4/TO with an N-terminal EGFP-FLAG-tag. Empty vectors were used as negative controls.

siRNAs

Small interfering RNAs (siRNAs) for human *NAMPT* (#1: 5′-CCACCGACUCCUACAAGGUUACUCA-3′, #2: 5′-GAUCUUCUCCAUACUGUCUUCAAGA-3′, #3: 5′-

GAAUAUUGAACUGGAAGCAGCACAU-3′), human *PPP6C* (#1: 5′-GACUACGUUUGUGACCUCCUCUUAG-3′, #2: 5′-GCAGCUUUAAUAGAUGAGCAGAUUU-3′) and human *ANKRD52* (#1: HSS178549, #2: HSS154260) were purchased as Stealth RNAi siRNAs from Invitrogen. For custom siRNAs, the target sequences were designed using the Block-iT RNAi Designer tool (Invitrogen, current URL https://rnaidesigner.thermofisher.com/rnaiexpress/). As the negative control for Stealth siRNAs, Stealth RNAi Negative Control Medium GC Duplex #2 (Invitrogen, Cat. #12935-112) was used. siRNA for human *NMNAT2* (#1: Cat. #D-008573-01, #2: Cat. #D-008573-02) were purchased as siGENOME siRNAs from Dharmacon. As the negative control for siGENOME siRNAs, siGENOME Non-Targeting siRNA #4 (Dharmacon, Cat. #D-001210-04) was used.

Protein purification

HEK293A cells were seeded in 10 cmφ dishes and transfected with EGFP-FLAG-tagged constructs. After washing with PBS, the cells were lysed in lysis buffer (20 mM Tris-HCl pH 7.5, 150 mM NaCl, 5 mM ethylene glycol-bis(2-aminoethylether)-*N*,*N*,*N*′,*N*′-tetraacetic acid (EGTA), 1% sodium deoxycholate, 1% Triton X-100 and 12 mM β-glycerophosphatase) supplemented with protease inhibitors (1 mM phenylmethylsulfonyl fluoride (PMSF) and 5 μg mL^{-1} leupeptin), phosphatase inhibitor cocktail I (8 mM NaF, 1 mM Na_3VO_4, 1.2 mM Na_2MoO_4, 5 μM cantharidin and 2 mM imidazole) and 1 mM dithiothreitol (DTT). The cell extracts were collected with a scraper from 3 dishes into a single microtube for each protein, followed by centrifugation at 4ºC and ~16,500 × *g* for 15 min. The supernatants were incubated with anti-FLAG antibody beads (Sigma-Aldrich, clone M2, Cat. #A2220) at 4ºC for ~3 hr. The beads were washed 4 times with wash buffer (20 mM Tris-HCl pH 7.5, 500 mM NaCl, 5 mM EGTA, 1% Triton X-100 and 2 mM DTT) and once with TBS (20 mM Tris-HCl pH 7.5, 150 mM NaCl and 1 mM DTT). The EGFP-FLAG-tagged proteins were eluted from the beads with 0.1 mg mL^{-1} 3x FLAG peptide (Sigma-Aldrich, Cat. #F4799) in TBS at 4ºC for more than 1 hr, followed by dilution to 40 μM with TBS. The concentration of the protein was estimated from the absorbance at 280 nm measured by a SimpliNano (GE healthcare) microvolume spectrophotometer with the extinction coefficient calculated by using the ExPASy ProtParam tool (https://web.expasy.org/protparam/).
