# Supplementary material for: Cells recognize osmotic stress through liquid–liquid phase separation lubricated with poly(ADP-ribose)
Source: Nat Commun. 2021 Mar 1;12:1353. doi: 10.1038/s41467-021-21614-5 (PMC7921423; doi:10.1038/s41467-021-21614-5)
Supplement: Supplementary file 3 — Description of Additional Supplementary Files [file 41467_2021_21614_MOESM3_ESM.pdf]

Description of Additional Supplementary Files

File Name: Supplementary Movie 1
Description: **A computational simulation for the relationship between the grid space and the number/size of ASK3 clusters.** Each frame was represented at every 10,000 iteration steps. Red squares: ASK3 units, black squares: obstacles.

File Name: Supplementary Movie 2
Description: **Dynamics and fusion of ASK3 condensates in Venus-ASK3-stably expressing HEK293A cells.** After 5 min, the cells were exposed to hyperosmotic stress (500 mOsm). White bar: 20 μm.

File Name: Supplementary Movie 3
Description: **A computational prediction for ASK3 cluster disassembly after the grid space expansion.** Each frame was represented at every 100,000 iteration steps. Red squares: ASK3 units, black squares: obstacles.

File Name: Supplementary Movie 4
Description: **Relationship between ANKRD52 and ASK3 condensates in HEK293A cells.** After 5 min, the cells were exposed to hyperosmotic stress (500 mOsm). Magenta: ASK3-tdTomato, green: ANKRD52-Venus, white bar: 20 μm.

File Name: Supplementary Data 1
Description: **List of key resources.** Key resources used in this study are indicated. IB: immunoblotting, IF: immunofluorescence, IEM: immunoelectron microscopy, CDS: cording sequence.

File Name: Supplementary Data 2
Description: **Summary of statistical analysis.** Statistical tests, the number of samples, the sample sizes, the test statistic, the degrees of freedom (Df) and (adjusted) *P* value are indicated.

File Name: Supplementary Software 1
Description: **In-silico-ASK3-condensates.** Python scripts for the computational simulations are included.
