# Supplementary figures and images for: Cells recognize osmotic stress through liquid–liquid phase separation lubricated with poly(ADP-ribose) (part 2 of 2)
Source: Nat Commun. 2021 Mar 1;12:1353. doi: 10.1038/s41467-021-21614-5 (PMC7921423; doi:10.1038/s41467-021-21614-5)

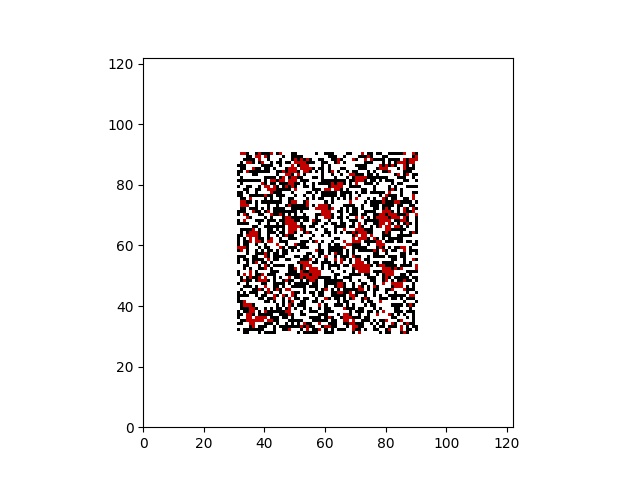

Supplement: Supplementary file 10 — Supplementary Software 1 [file 41467_2021_21614_MOESM10_ESM.zip › SupplementaryCode/DemoResult/Volume_60/seq00265.jpg]

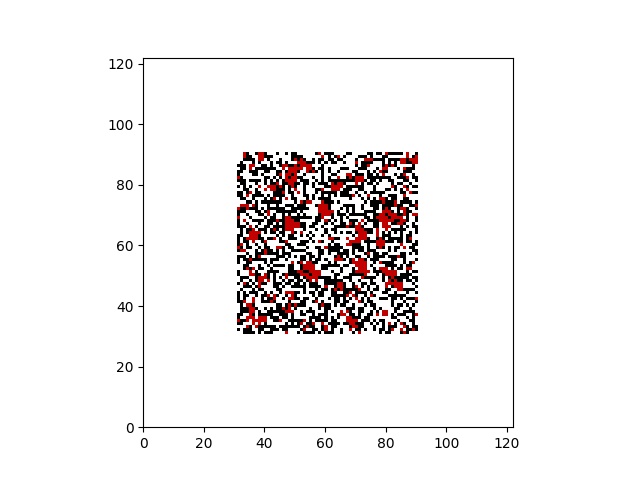

Supplement: Supplementary file 10 — Supplementary Software 1 [file 41467_2021_21614_MOESM10_ESM.zip › SupplementaryCode/DemoResult/Volume_60/seq00259.jpg]

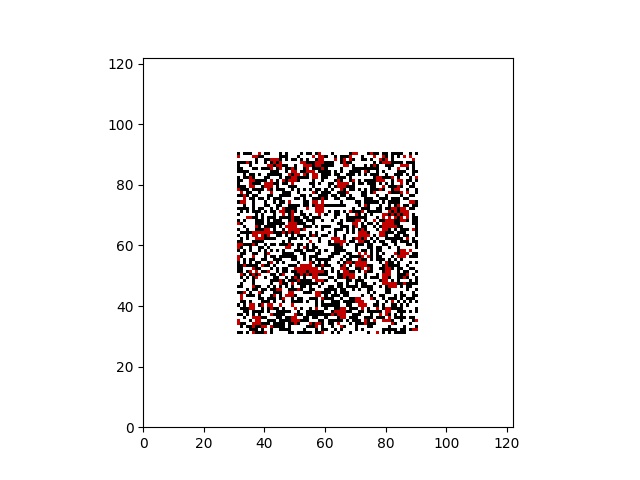

Supplement: Supplementary file 10 — Supplementary Software 1 [file 41467_2021_21614_MOESM10_ESM.zip › SupplementaryCode/DemoResult/Volume_60/seq00098.jpg]

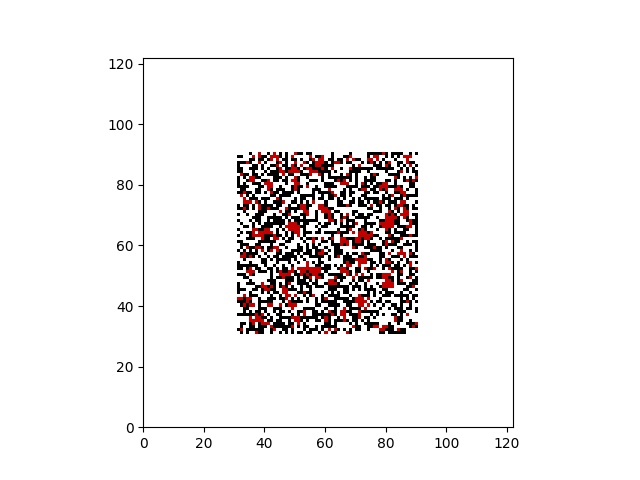

Supplement: Supplementary file 10 — Supplementary Software 1 [file 41467_2021_21614_MOESM10_ESM.zip › SupplementaryCode/DemoResult/Volume_60/seq00073.jpg]

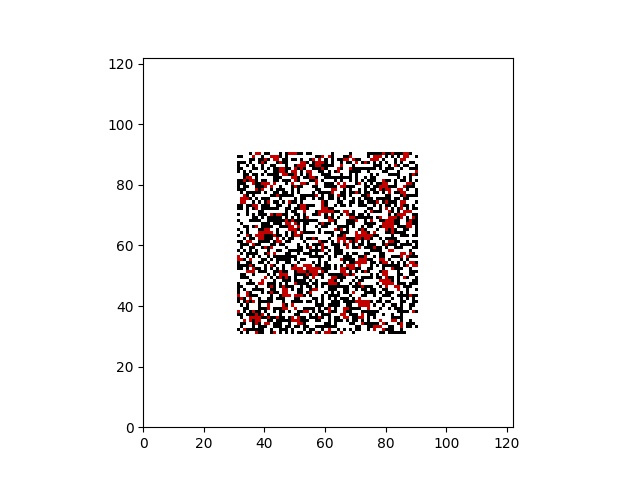

Supplement: Supplementary file 10 — Supplementary Software 1 [file 41467_2021_21614_MOESM10_ESM.zip › SupplementaryCode/DemoResult/Volume_60/seq00067.jpg]

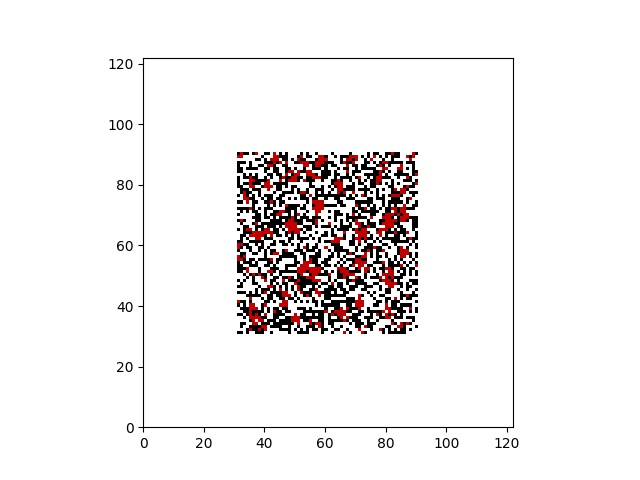

Supplement: Supplementary file 10 — Supplementary Software 1 [file 41467_2021_21614_MOESM10_ESM.zip › SupplementaryCode/DemoResult/Volume_60/seq00107.jpg]

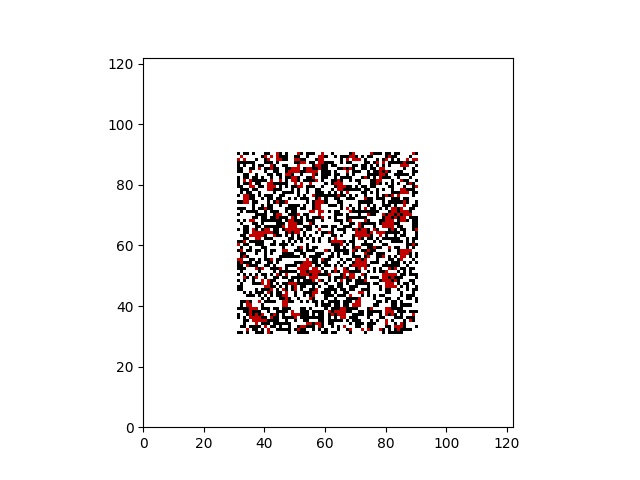

Supplement: Supplementary file 10 — Supplementary Software 1 [file 41467_2021_21614_MOESM10_ESM.zip › SupplementaryCode/DemoResult/Volume_60/seq00113.jpg]

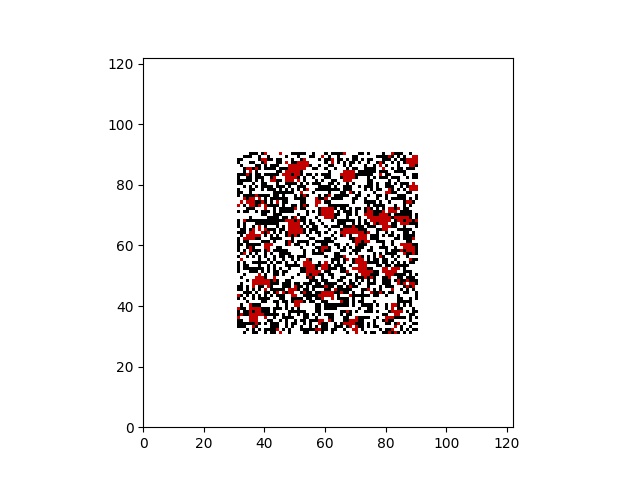

Supplement: Supplementary file 10 — Supplementary Software 1 [file 41467_2021_21614_MOESM10_ESM.zip › SupplementaryCode/DemoResult/Volume_60/seq00488.jpg]

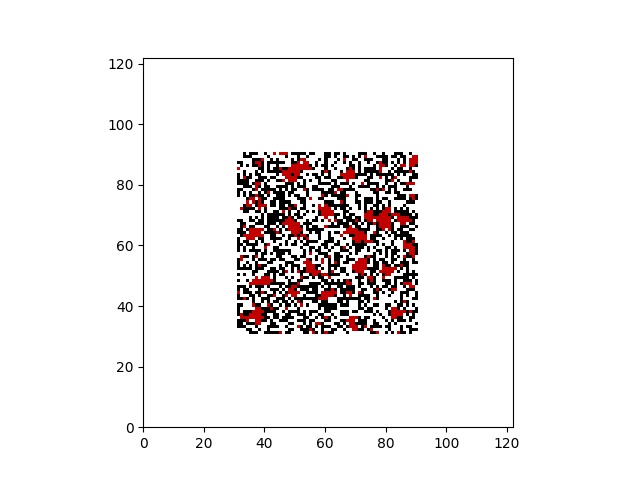

Supplement: Supplementary file 10 — Supplementary Software 1 [file 41467_2021_21614_MOESM10_ESM.zip › SupplementaryCode/DemoResult/Volume_60/seq00463.jpg]

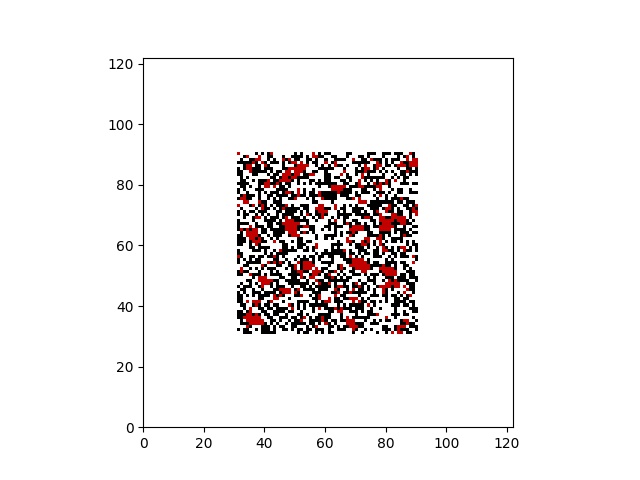

Supplement: Supplementary file 10 — Supplementary Software 1 [file 41467_2021_21614_MOESM10_ESM.zip › SupplementaryCode/DemoResult/Volume_60/seq00305.jpg]

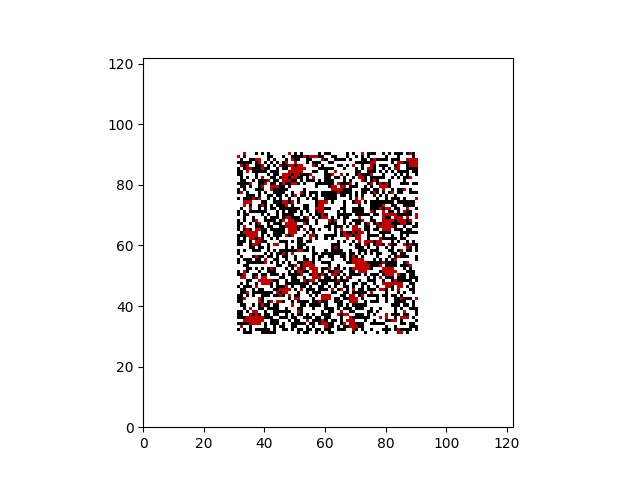

Supplement: Supplementary file 10 — Supplementary Software 1 [file 41467_2021_21614_MOESM10_ESM.zip › SupplementaryCode/DemoResult/Volume_60/seq00311.jpg]

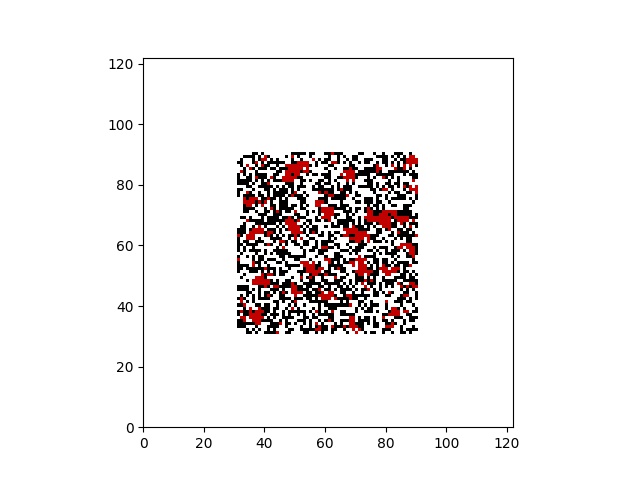

Supplement: Supplementary file 10 — Supplementary Software 1 [file 41467_2021_21614_MOESM10_ESM.zip › SupplementaryCode/DemoResult/Volume_60/seq00477.jpg]

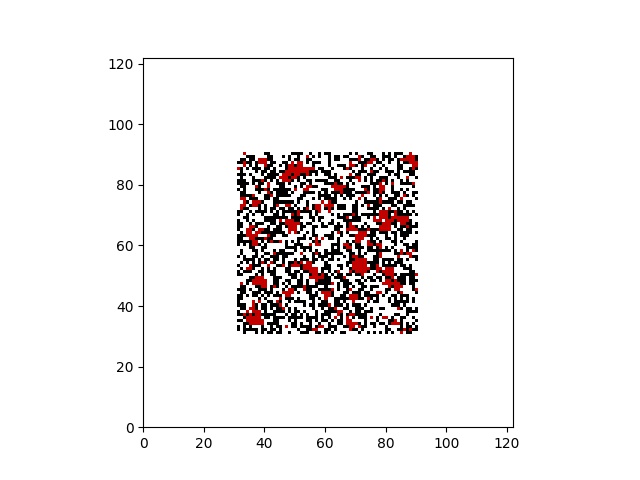

Supplement: Supplementary file 10 — Supplementary Software 1 [file 41467_2021_21614_MOESM10_ESM.zip › SupplementaryCode/DemoResult/Volume_60/seq00339.jpg]

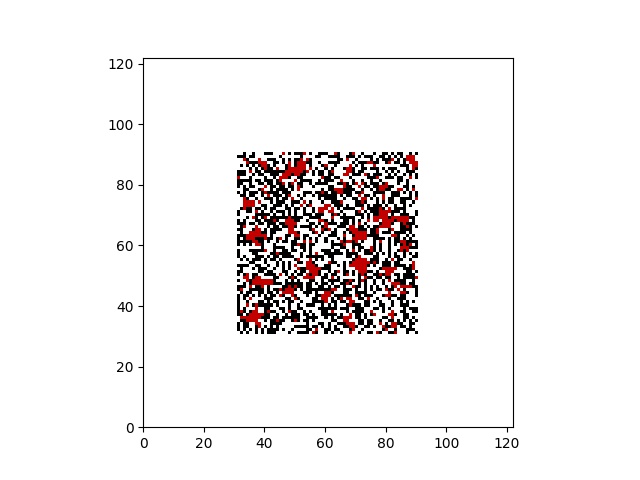

Supplement: Supplementary file 10 — Supplementary Software 1 [file 41467_2021_21614_MOESM10_ESM.zip › SupplementaryCode/DemoResult/Volume_60/seq00376.jpg]

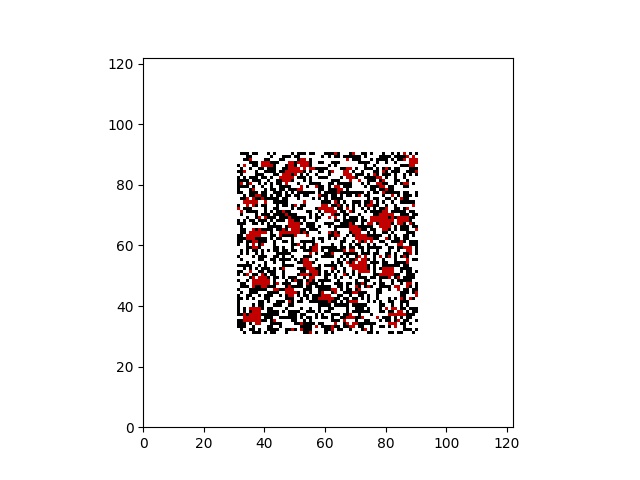

Supplement: Supplementary file 10 — Supplementary Software 1 [file 41467_2021_21614_MOESM10_ESM.zip › SupplementaryCode/DemoResult/Volume_60/seq00410.jpg]

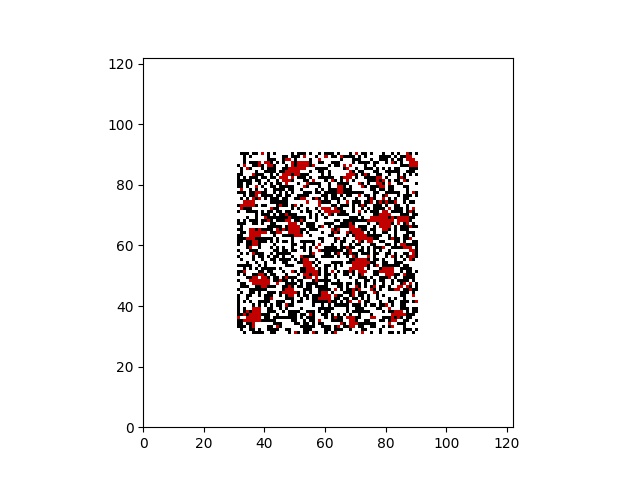

Supplement: Supplementary file 10 — Supplementary Software 1 [file 41467_2021_21614_MOESM10_ESM.zip › SupplementaryCode/DemoResult/Volume_60/seq00404.jpg]

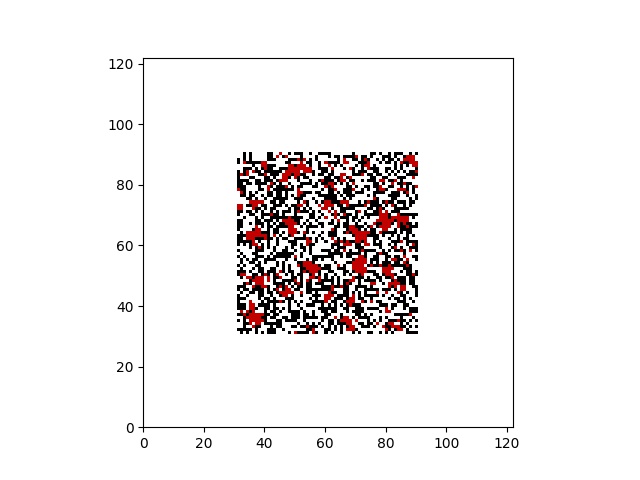

Supplement: Supplementary file 10 — Supplementary Software 1 [file 41467_2021_21614_MOESM10_ESM.zip › SupplementaryCode/DemoResult/Volume_60/seq00362.jpg]

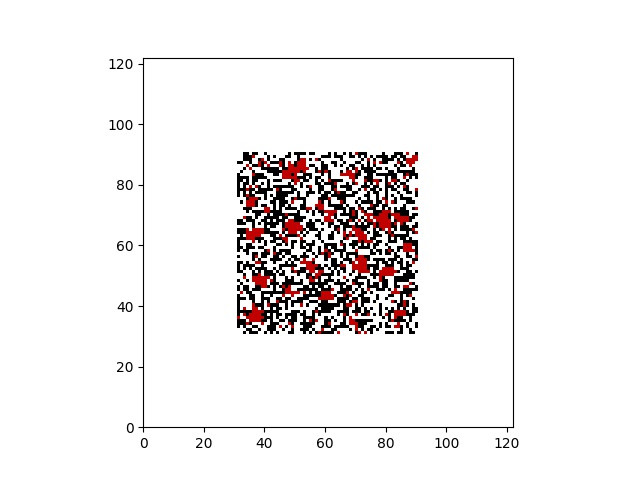

Supplement: Supplementary file 10 — Supplementary Software 1 [file 41467_2021_21614_MOESM10_ESM.zip › SupplementaryCode/DemoResult/Volume_60/seq00438.jpg]

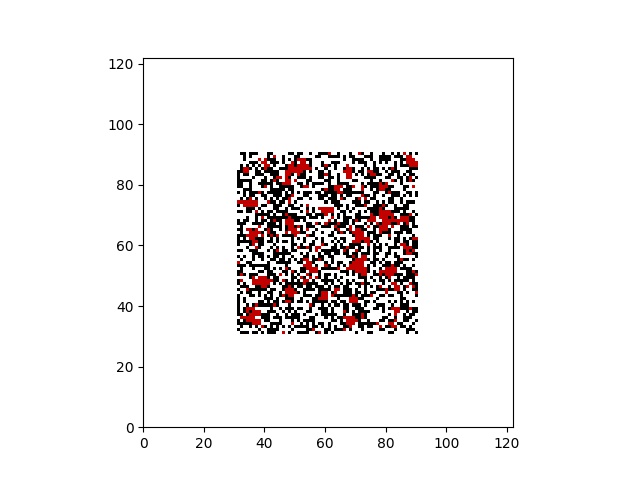

Supplement: Supplementary file 10 — Supplementary Software 1 [file 41467_2021_21614_MOESM10_ESM.zip › SupplementaryCode/DemoResult/Volume_60/seq00389.jpg]

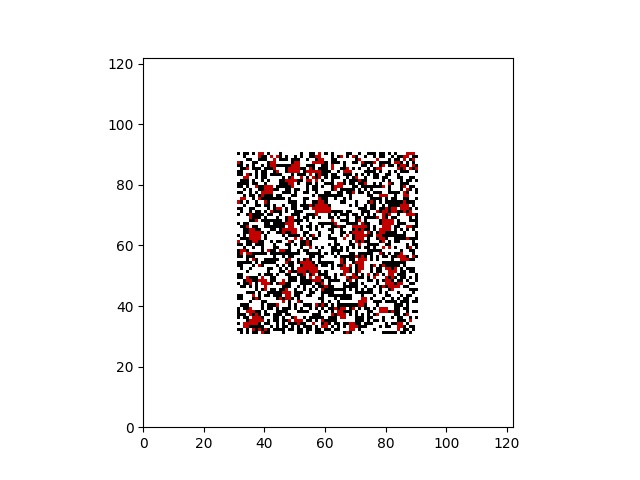

Supplement: Supplementary file 10 — Supplementary Software 1 [file 41467_2021_21614_MOESM10_ESM.zip › SupplementaryCode/DemoResult/Volume_60/seq00174.jpg]

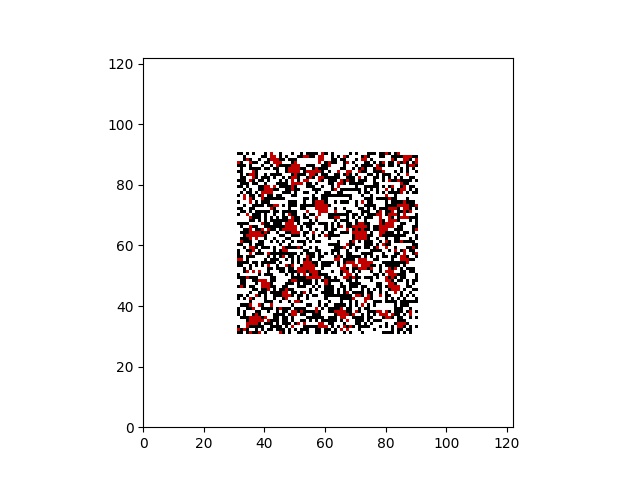

Supplement: Supplementary file 10 — Supplementary Software 1 [file 41467_2021_21614_MOESM10_ESM.zip › SupplementaryCode/DemoResult/Volume_60/seq00160.jpg]

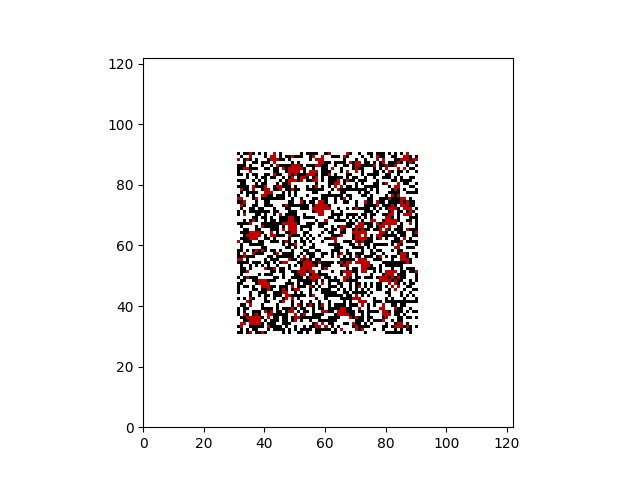

Supplement: Supplementary file 10 — Supplementary Software 1 [file 41467_2021_21614_MOESM10_ESM.zip › SupplementaryCode/DemoResult/Volume_60/seq00148.jpg]

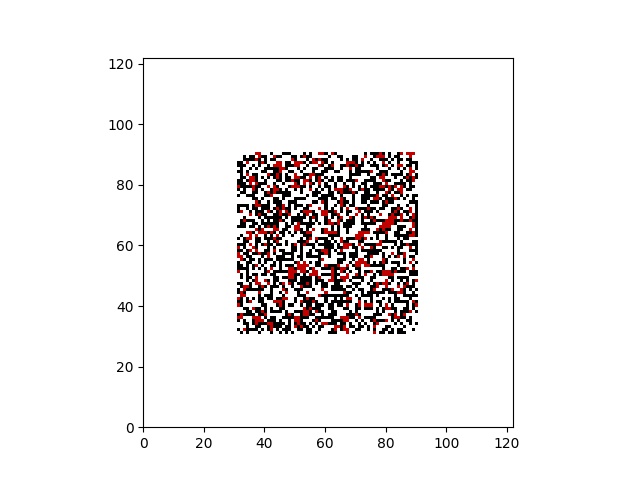

Supplement: Supplementary file 10 — Supplementary Software 1 [file 41467_2021_21614_MOESM10_ESM.zip › SupplementaryCode/DemoResult/Volume_60/seq00014.jpg]

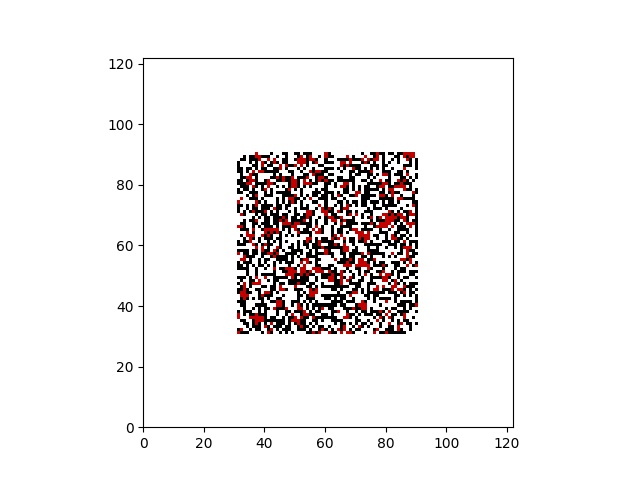

Supplement: Supplementary file 10 — Supplementary Software 1 [file 41467_2021_21614_MOESM10_ESM.zip › SupplementaryCode/DemoResult/Volume_60/seq00028.jpg]

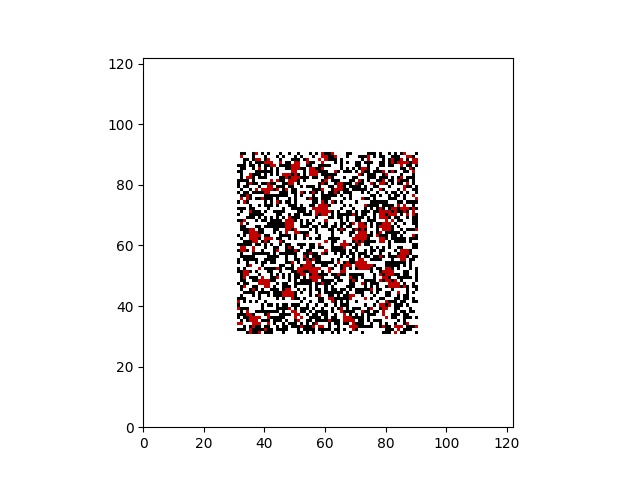

Supplement: Supplementary file 10 — Supplementary Software 1 [file 41467_2021_21614_MOESM10_ESM.zip › SupplementaryCode/DemoResult/Volume_60/seq00202.jpg]

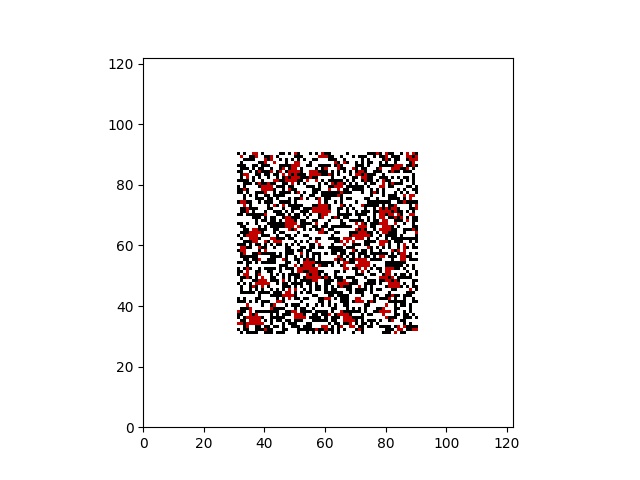

Supplement: Supplementary file 10 — Supplementary Software 1 [file 41467_2021_21614_MOESM10_ESM.zip › SupplementaryCode/DemoResult/Volume_60/seq00216.jpg]

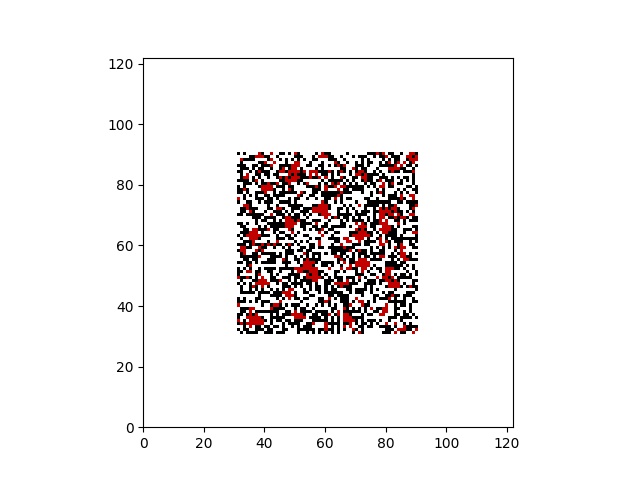

Supplement: Supplementary file 10 — Supplementary Software 1 [file 41467_2021_21614_MOESM10_ESM.zip › SupplementaryCode/DemoResult/Volume_60/seq00217.jpg]

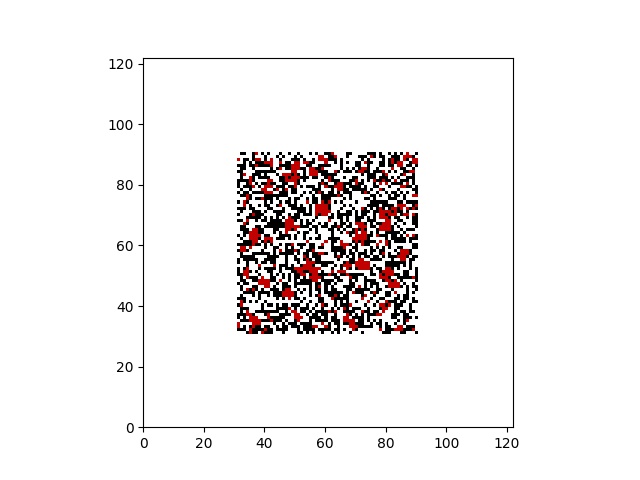

Supplement: Supplementary file 10 — Supplementary Software 1 [file 41467_2021_21614_MOESM10_ESM.zip › SupplementaryCode/DemoResult/Volume_60/seq00203.jpg]

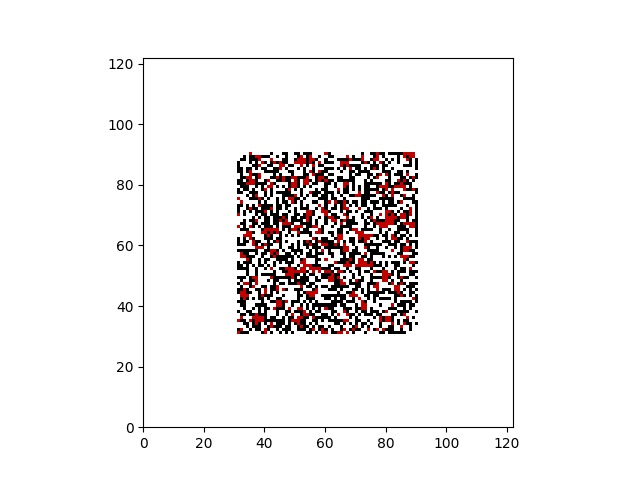

Supplement: Supplementary file 10 — Supplementary Software 1 [file 41467_2021_21614_MOESM10_ESM.zip › SupplementaryCode/DemoResult/Volume_60/seq00029.jpg]

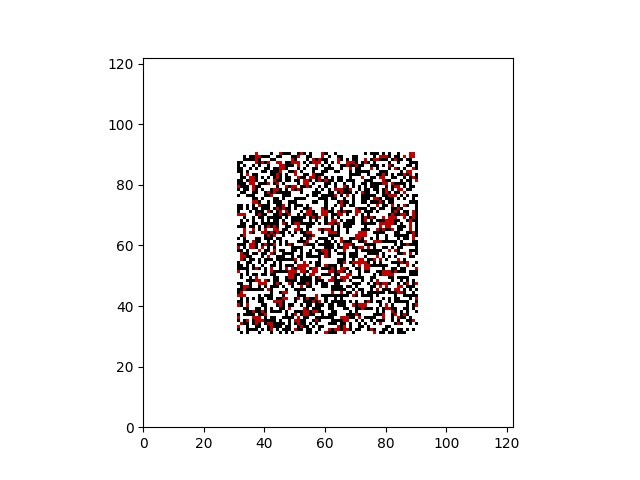

Supplement: Supplementary file 10 — Supplementary Software 1 [file 41467_2021_21614_MOESM10_ESM.zip › SupplementaryCode/DemoResult/Volume_60/seq00015.jpg]

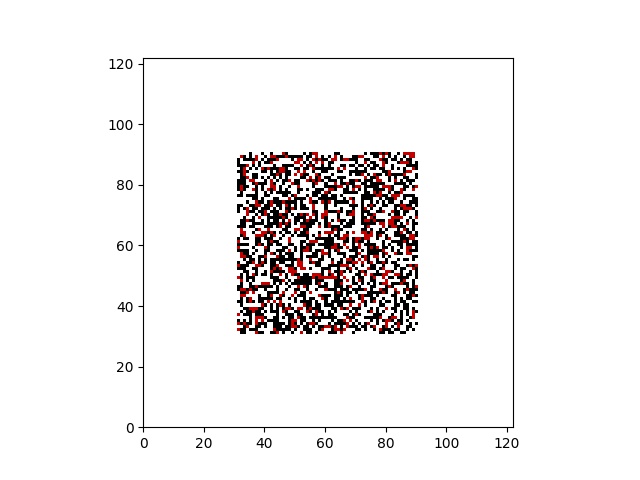

Supplement: Supplementary file 10 — Supplementary Software 1 [file 41467_2021_21614_MOESM10_ESM.zip › SupplementaryCode/DemoResult/Volume_60/seq00001.jpg]

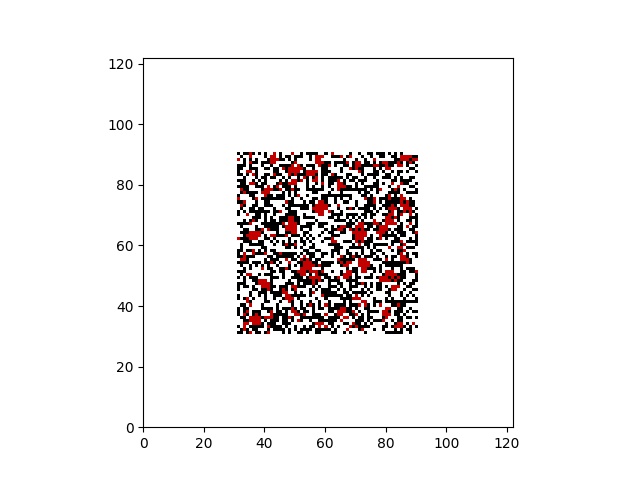

Supplement: Supplementary file 10 — Supplementary Software 1 [file 41467_2021_21614_MOESM10_ESM.zip › SupplementaryCode/DemoResult/Volume_60/seq00149.jpg]

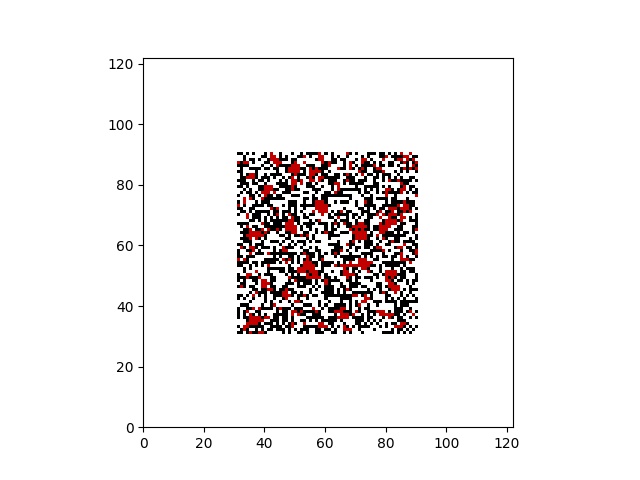

Supplement: Supplementary file 10 — Supplementary Software 1 [file 41467_2021_21614_MOESM10_ESM.zip › SupplementaryCode/DemoResult/Volume_60/seq00161.jpg]

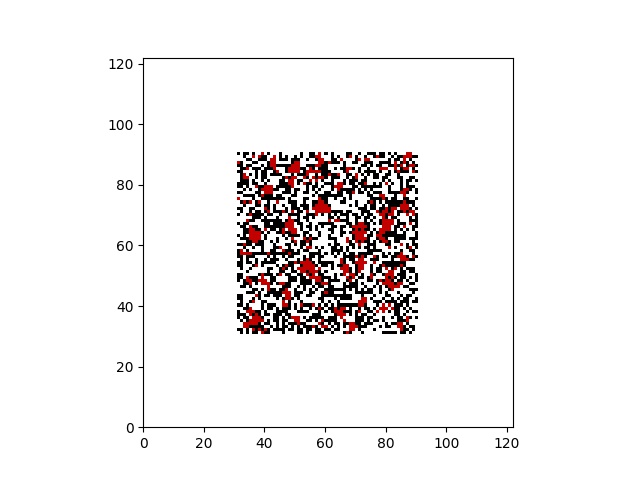

Supplement: Supplementary file 10 — Supplementary Software 1 [file 41467_2021_21614_MOESM10_ESM.zip › SupplementaryCode/DemoResult/Volume_60/seq00175.jpg]

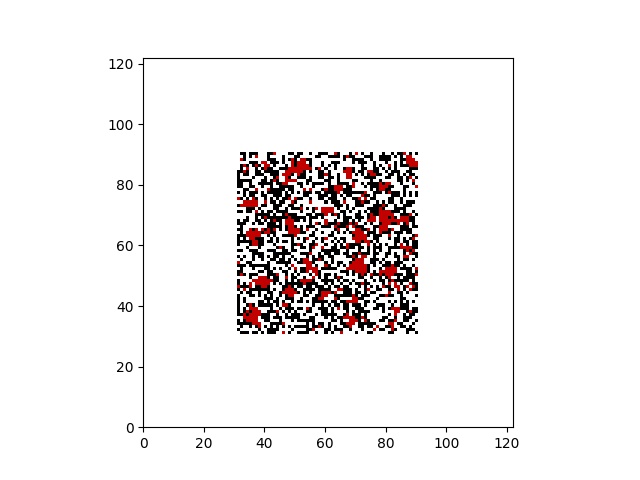

Supplement: Supplementary file 10 — Supplementary Software 1 [file 41467_2021_21614_MOESM10_ESM.zip › SupplementaryCode/DemoResult/Volume_60/seq00388.jpg]

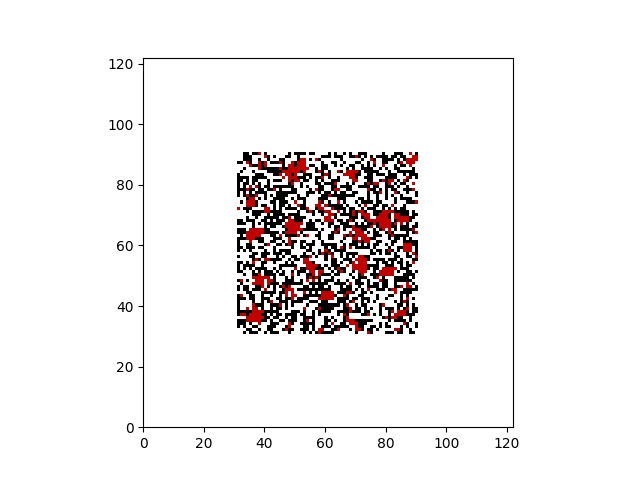

Supplement: Supplementary file 10 — Supplementary Software 1 [file 41467_2021_21614_MOESM10_ESM.zip › SupplementaryCode/DemoResult/Volume_60/seq00439.jpg]

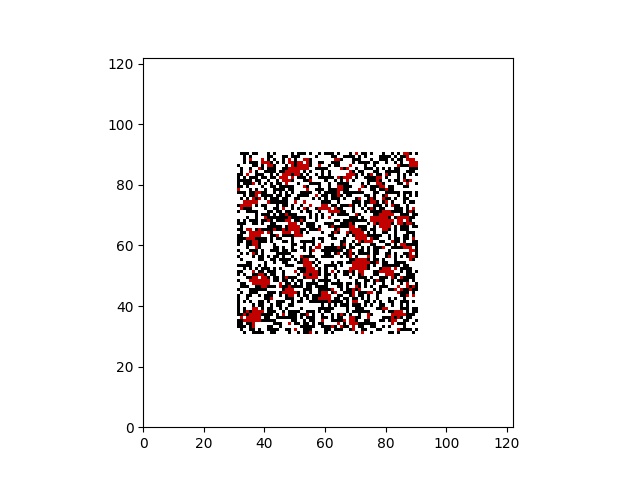

Supplement: Supplementary file 10 — Supplementary Software 1 [file 41467_2021_21614_MOESM10_ESM.zip › SupplementaryCode/DemoResult/Volume_60/seq00405.jpg]

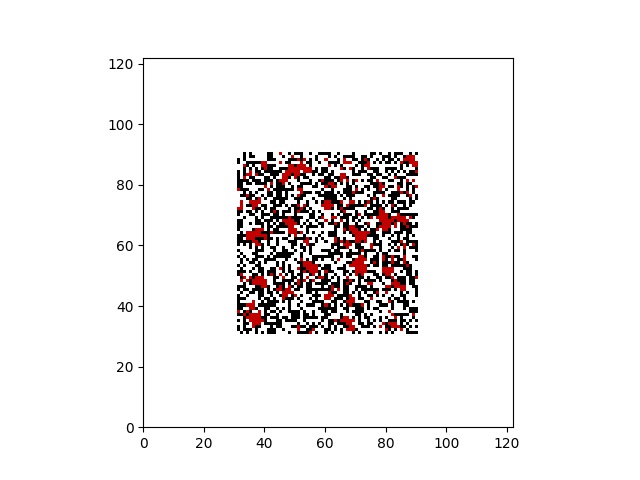

Supplement: Supplementary file 10 — Supplementary Software 1 [file 41467_2021_21614_MOESM10_ESM.zip › SupplementaryCode/DemoResult/Volume_60/seq00363.jpg]

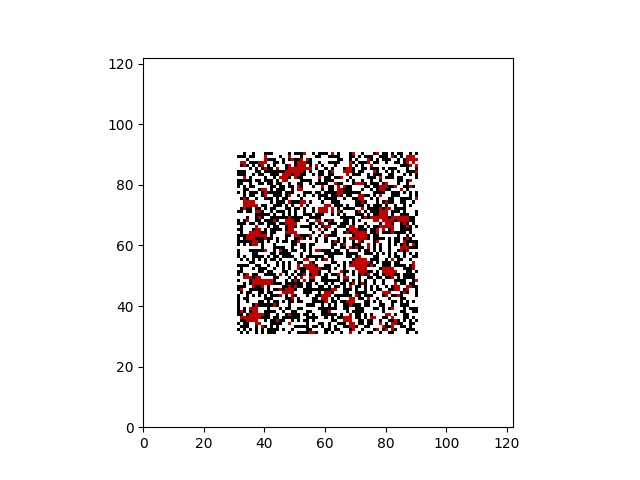

Supplement: Supplementary file 10 — Supplementary Software 1 [file 41467_2021_21614_MOESM10_ESM.zip › SupplementaryCode/DemoResult/Volume_60/seq00377.jpg]

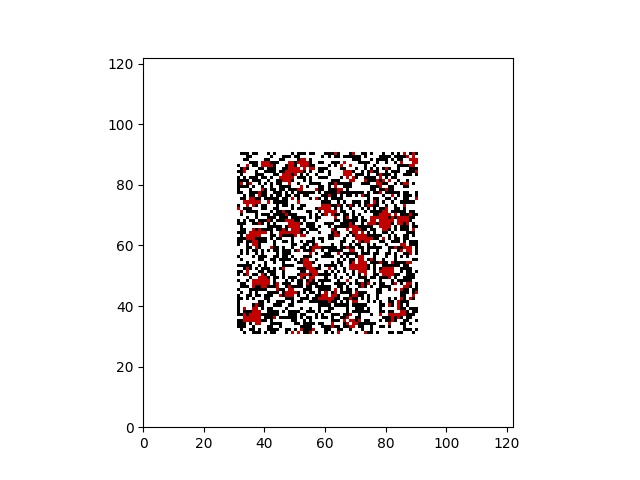

Supplement: Supplementary file 10 — Supplementary Software 1 [file 41467_2021_21614_MOESM10_ESM.zip › SupplementaryCode/DemoResult/Volume_60/seq00411.jpg]

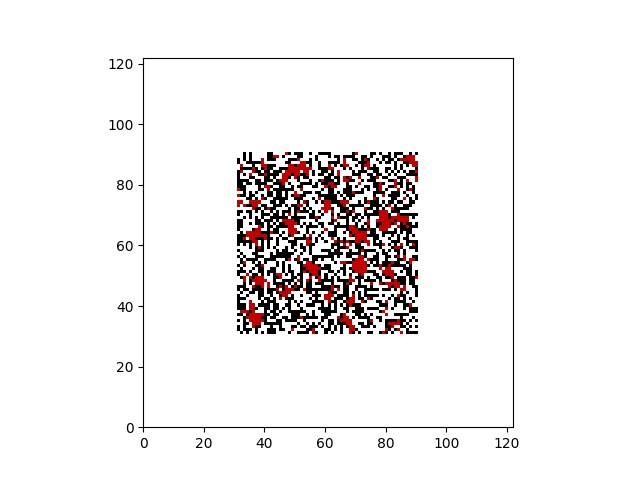

Supplement: Supplementary file 10 — Supplementary Software 1 [file 41467_2021_21614_MOESM10_ESM.zip › SupplementaryCode/DemoResult/Volume_60/seq00361.jpg]

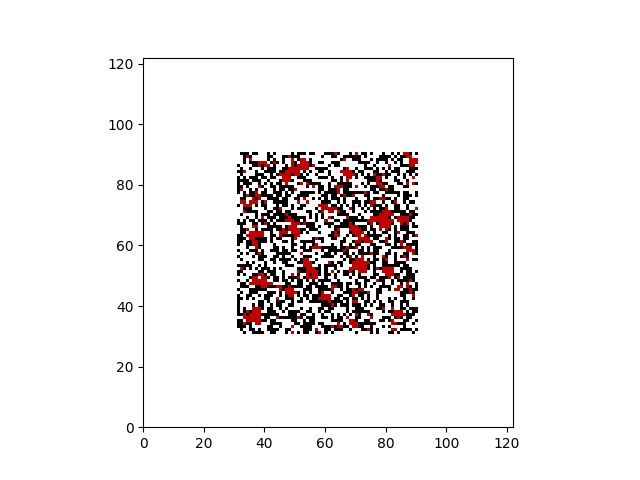

Supplement: Supplementary file 10 — Supplementary Software 1 [file 41467_2021_21614_MOESM10_ESM.zip › SupplementaryCode/DemoResult/Volume_60/seq00407.jpg]

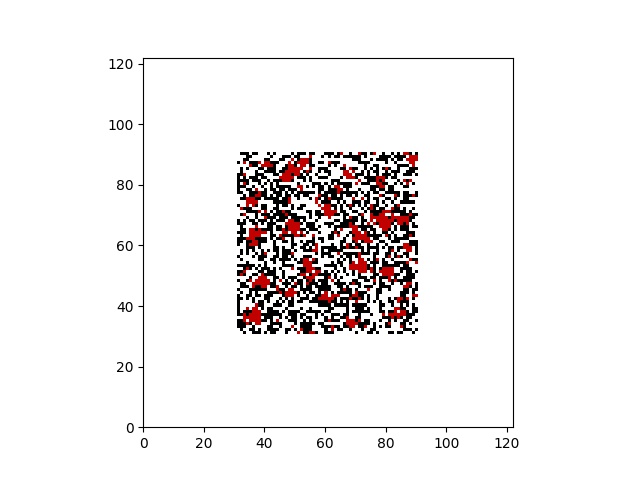

Supplement: Supplementary file 10 — Supplementary Software 1 [file 41467_2021_21614_MOESM10_ESM.zip › SupplementaryCode/DemoResult/Volume_60/seq00413.jpg]

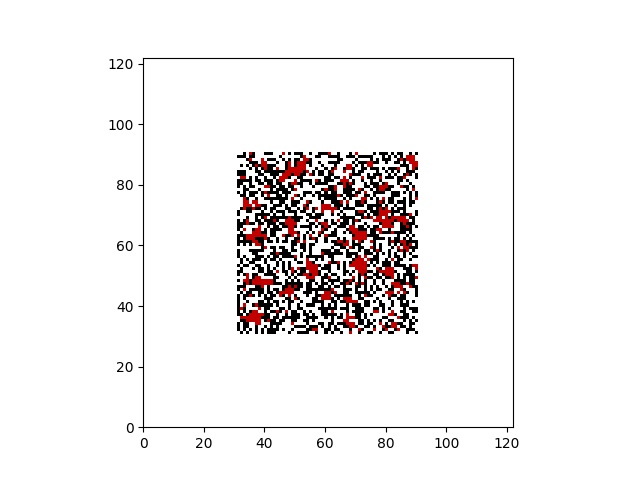

Supplement: Supplementary file 10 — Supplementary Software 1 [file 41467_2021_21614_MOESM10_ESM.zip › SupplementaryCode/DemoResult/Volume_60/seq00375.jpg]

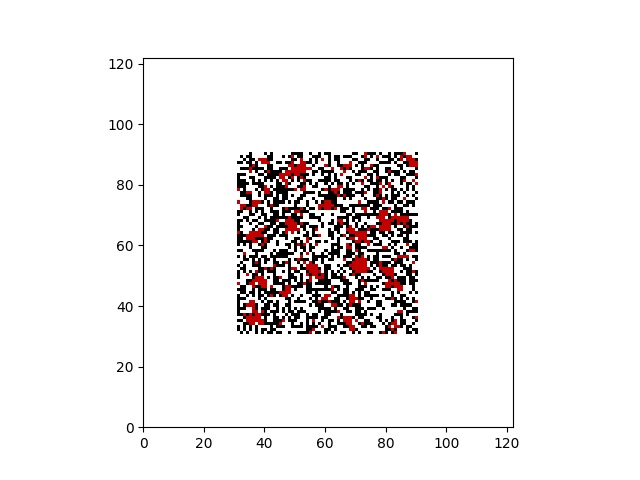

Supplement: Supplementary file 10 — Supplementary Software 1 [file 41467_2021_21614_MOESM10_ESM.zip › SupplementaryCode/DemoResult/Volume_60/seq00349.jpg]

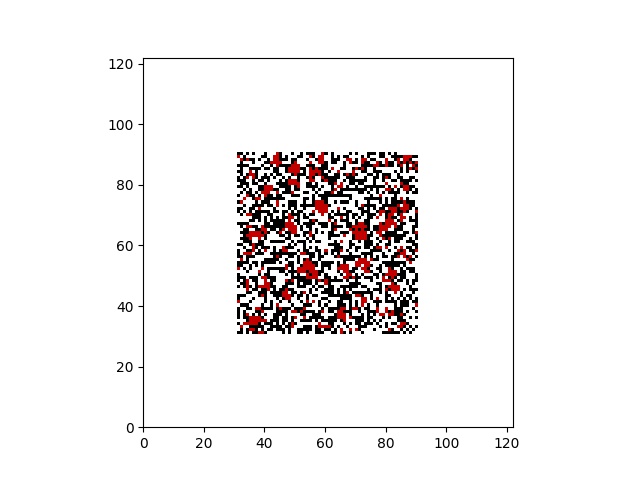

Supplement: Supplementary file 10 — Supplementary Software 1 [file 41467_2021_21614_MOESM10_ESM.zip › SupplementaryCode/DemoResult/Volume_60/seq00163.jpg]

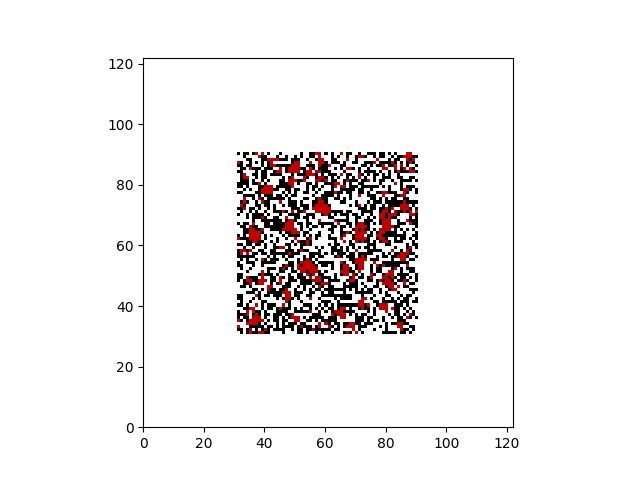

Supplement: Supplementary file 10 — Supplementary Software 1 [file 41467_2021_21614_MOESM10_ESM.zip › SupplementaryCode/DemoResult/Volume_60/seq00177.jpg]

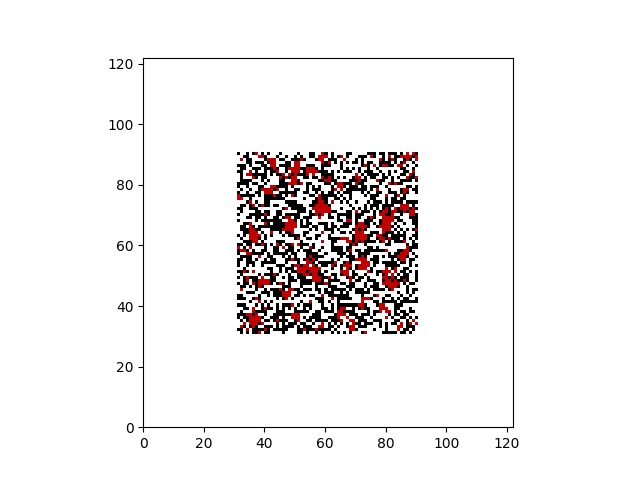

Supplement: Supplementary file 10 — Supplementary Software 1 [file 41467_2021_21614_MOESM10_ESM.zip › SupplementaryCode/DemoResult/Volume_60/seq00188.jpg]

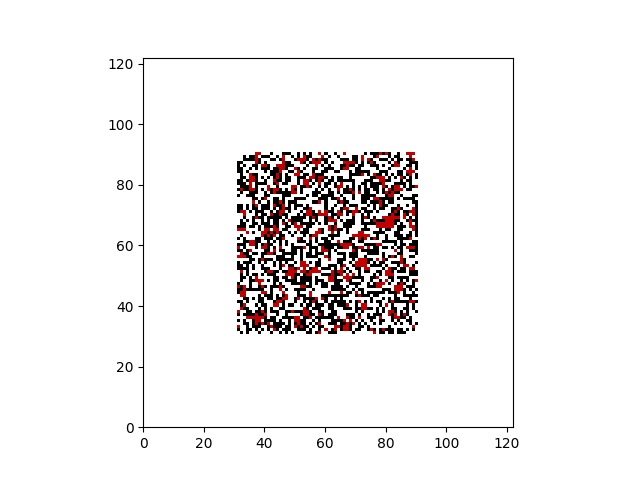

Supplement: Supplementary file 10 — Supplementary Software 1 [file 41467_2021_21614_MOESM10_ESM.zip › SupplementaryCode/DemoResult/Volume_60/seq00017.jpg]

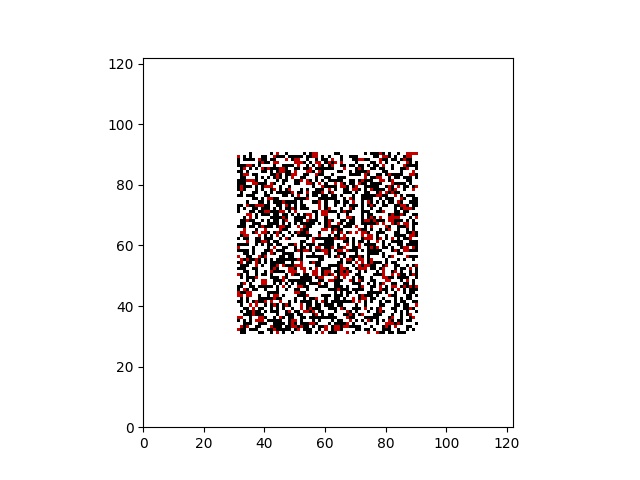

Supplement: Supplementary file 10 — Supplementary Software 1 [file 41467_2021_21614_MOESM10_ESM.zip › SupplementaryCode/DemoResult/Volume_60/seq00003.jpg]

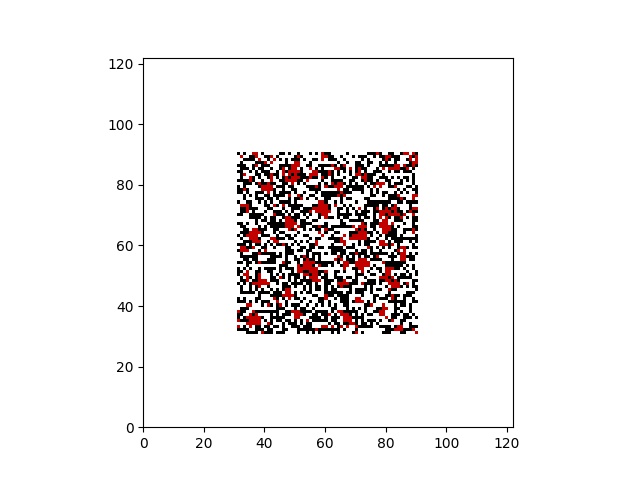

Supplement: Supplementary file 10 — Supplementary Software 1 [file 41467_2021_21614_MOESM10_ESM.zip › SupplementaryCode/DemoResult/Volume_60/seq00215.jpg]

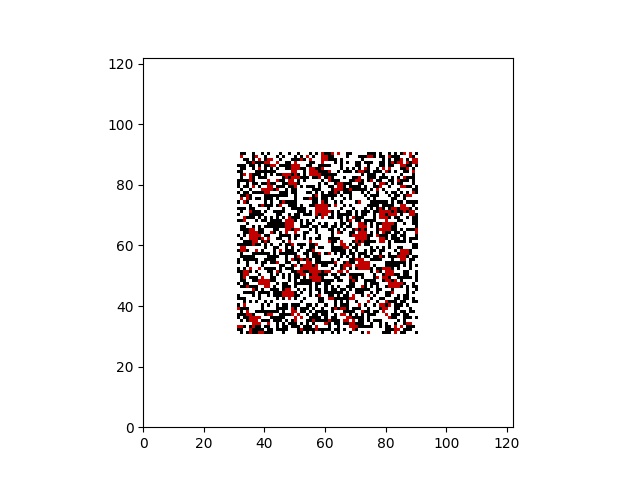

Supplement: Supplementary file 10 — Supplementary Software 1 [file 41467_2021_21614_MOESM10_ESM.zip › SupplementaryCode/DemoResult/Volume_60/seq00201.jpg]

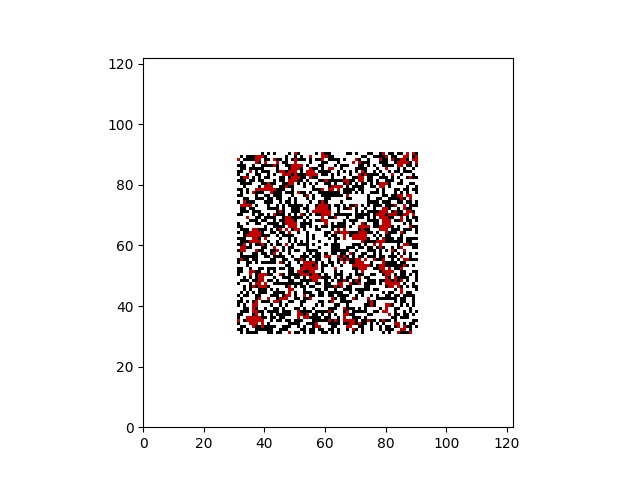

Supplement: Supplementary file 10 — Supplementary Software 1 [file 41467_2021_21614_MOESM10_ESM.zip › SupplementaryCode/DemoResult/Volume_60/seq00229.jpg]

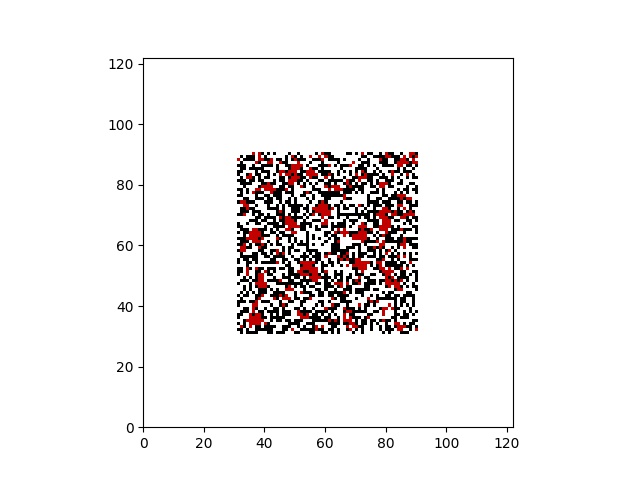

Supplement: Supplementary file 10 — Supplementary Software 1 [file 41467_2021_21614_MOESM10_ESM.zip › SupplementaryCode/DemoResult/Volume_60/seq00228.jpg]

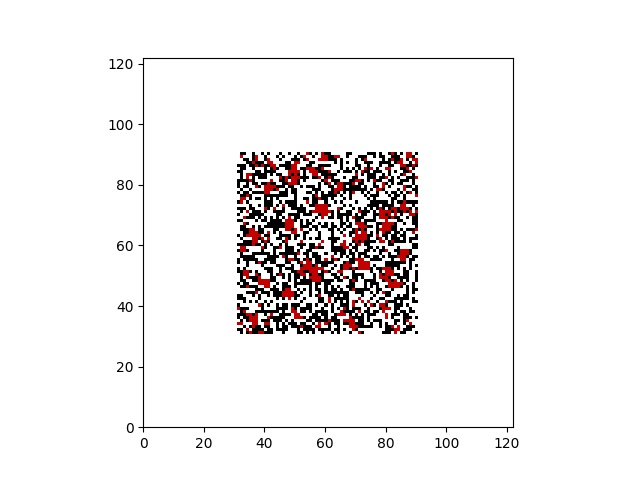

Supplement: Supplementary file 10 — Supplementary Software 1 [file 41467_2021_21614_MOESM10_ESM.zip › SupplementaryCode/DemoResult/Volume_60/seq00200.jpg]

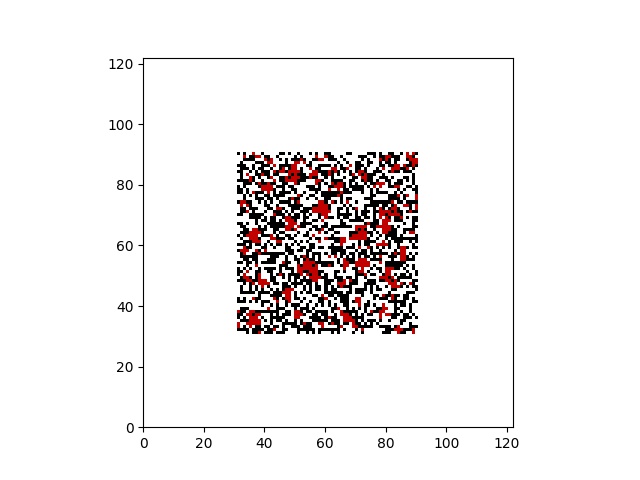

Supplement: Supplementary file 10 — Supplementary Software 1 [file 41467_2021_21614_MOESM10_ESM.zip › SupplementaryCode/DemoResult/Volume_60/seq00214.jpg]

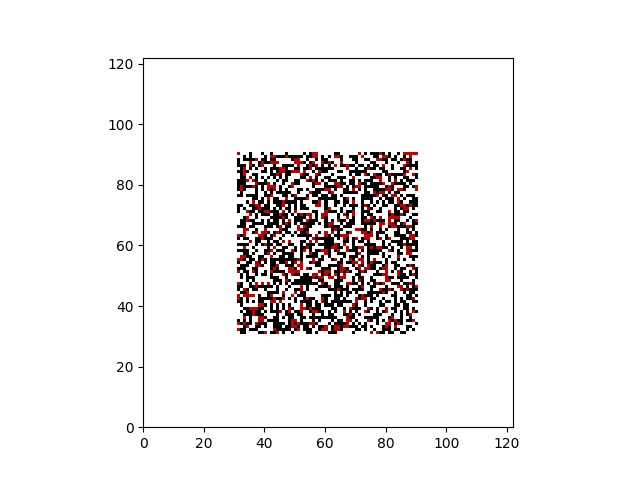

Supplement: Supplementary file 10 — Supplementary Software 1 [file 41467_2021_21614_MOESM10_ESM.zip › SupplementaryCode/DemoResult/Volume_60/seq00002.jpg]

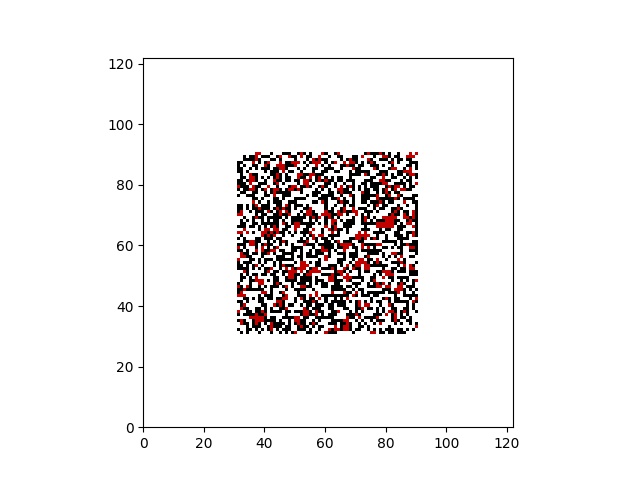

Supplement: Supplementary file 10 — Supplementary Software 1 [file 41467_2021_21614_MOESM10_ESM.zip › SupplementaryCode/DemoResult/Volume_60/seq00016.jpg]

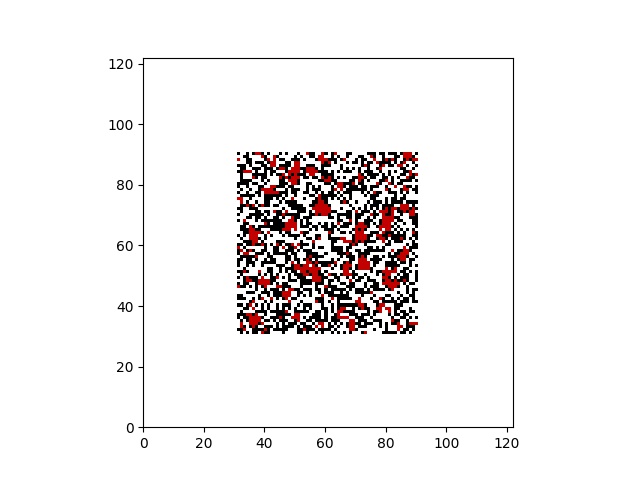

Supplement: Supplementary file 10 — Supplementary Software 1 [file 41467_2021_21614_MOESM10_ESM.zip › SupplementaryCode/DemoResult/Volume_60/seq00189.jpg]

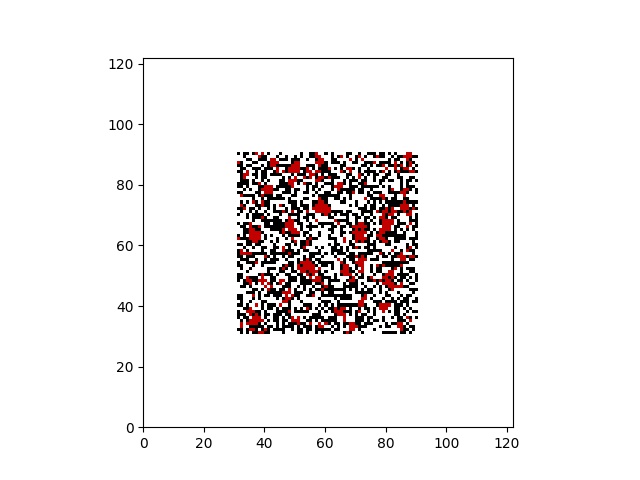

Supplement: Supplementary file 10 — Supplementary Software 1 [file 41467_2021_21614_MOESM10_ESM.zip › SupplementaryCode/DemoResult/Volume_60/seq00176.jpg]

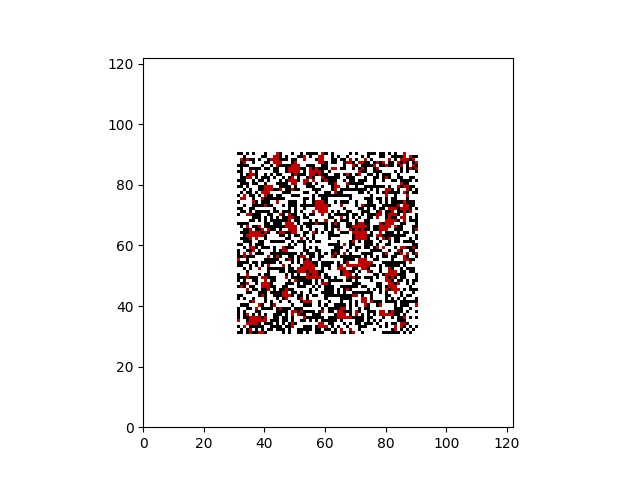

Supplement: Supplementary file 10 — Supplementary Software 1 [file 41467_2021_21614_MOESM10_ESM.zip › SupplementaryCode/DemoResult/Volume_60/seq00162.jpg]

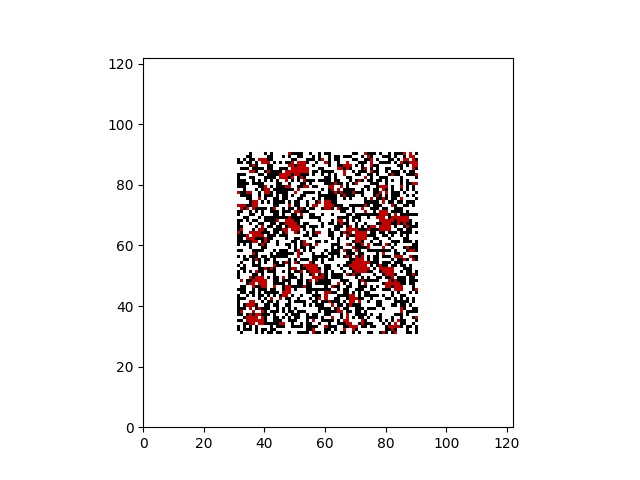

Supplement: Supplementary file 10 — Supplementary Software 1 [file 41467_2021_21614_MOESM10_ESM.zip › SupplementaryCode/DemoResult/Volume_60/seq00348.jpg]

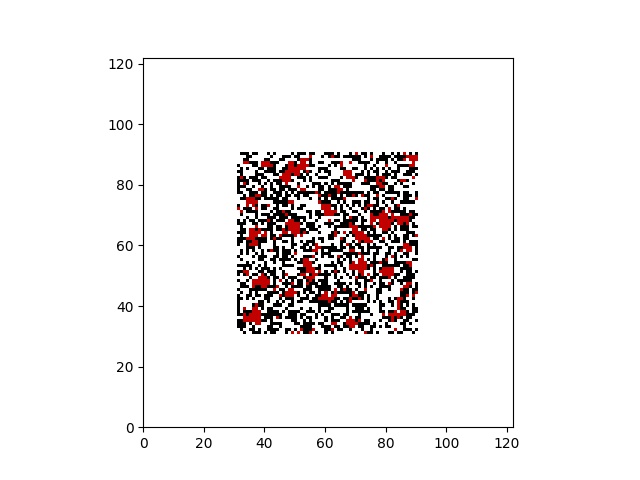

Supplement: Supplementary file 10 — Supplementary Software 1 [file 41467_2021_21614_MOESM10_ESM.zip › SupplementaryCode/DemoResult/Volume_60/seq00412.jpg]

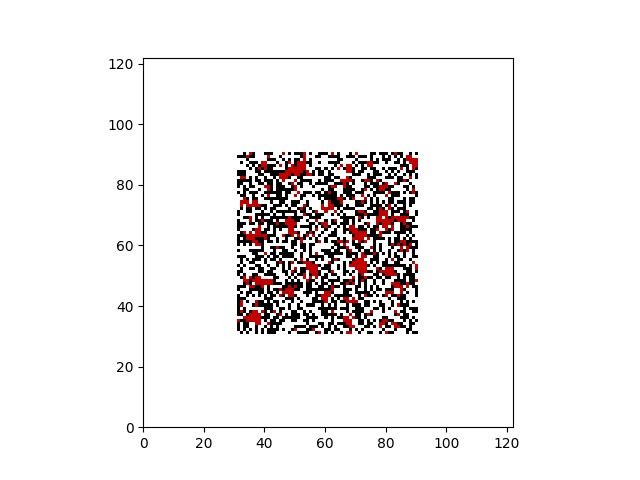

Supplement: Supplementary file 10 — Supplementary Software 1 [file 41467_2021_21614_MOESM10_ESM.zip › SupplementaryCode/DemoResult/Volume_60/seq00374.jpg]

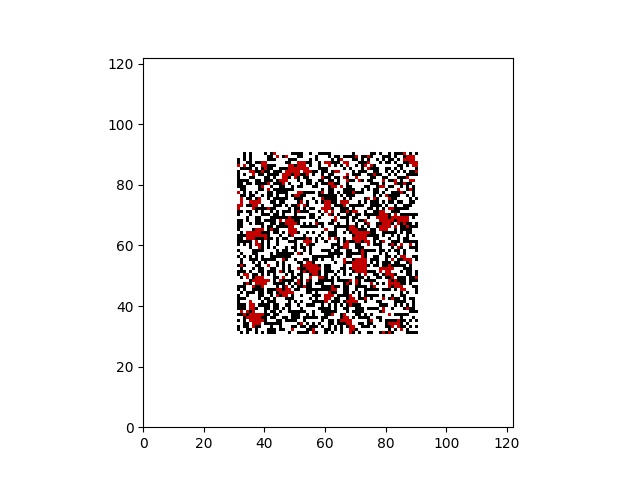

Supplement: Supplementary file 10 — Supplementary Software 1 [file 41467_2021_21614_MOESM10_ESM.zip › SupplementaryCode/DemoResult/Volume_60/seq00360.jpg]

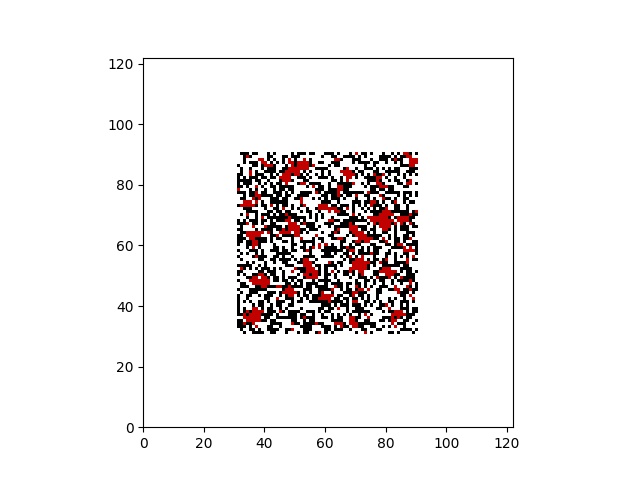

Supplement: Supplementary file 10 — Supplementary Software 1 [file 41467_2021_21614_MOESM10_ESM.zip › SupplementaryCode/DemoResult/Volume_60/seq00406.jpg]

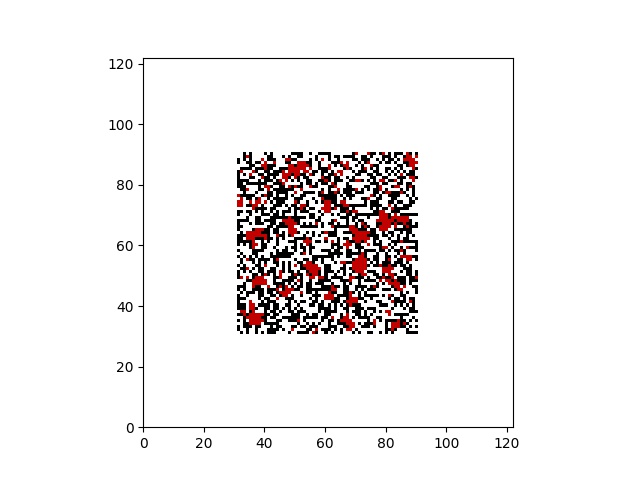

Supplement: Supplementary file 10 — Supplementary Software 1 [file 41467_2021_21614_MOESM10_ESM.zip › SupplementaryCode/DemoResult/Volume_60/seq00358.jpg]

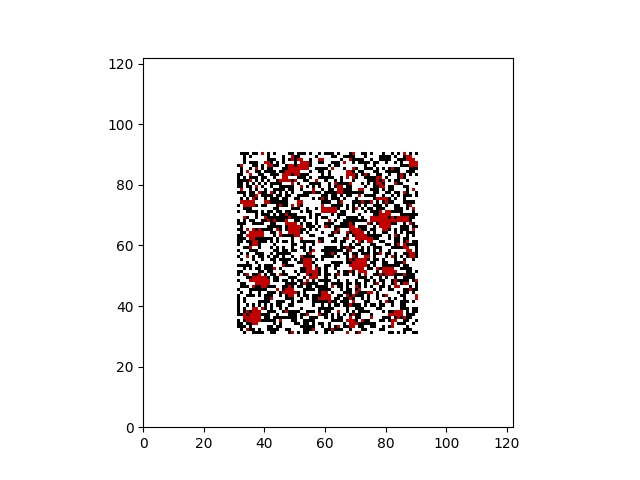

Supplement: Supplementary file 10 — Supplementary Software 1 [file 41467_2021_21614_MOESM10_ESM.zip › SupplementaryCode/DemoResult/Volume_60/seq00402.jpg]

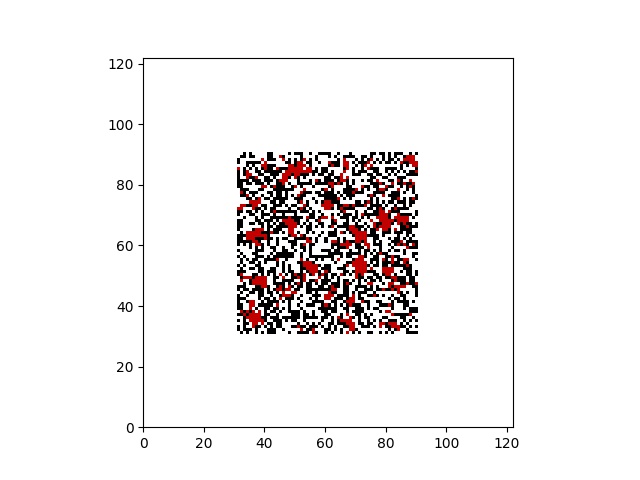

Supplement: Supplementary file 10 — Supplementary Software 1 [file 41467_2021_21614_MOESM10_ESM.zip › SupplementaryCode/DemoResult/Volume_60/seq00364.jpg]

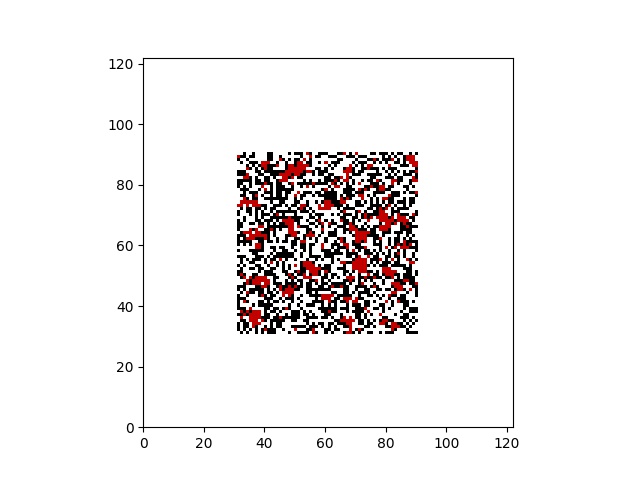

Supplement: Supplementary file 10 — Supplementary Software 1 [file 41467_2021_21614_MOESM10_ESM.zip › SupplementaryCode/DemoResult/Volume_60/seq00370.jpg]

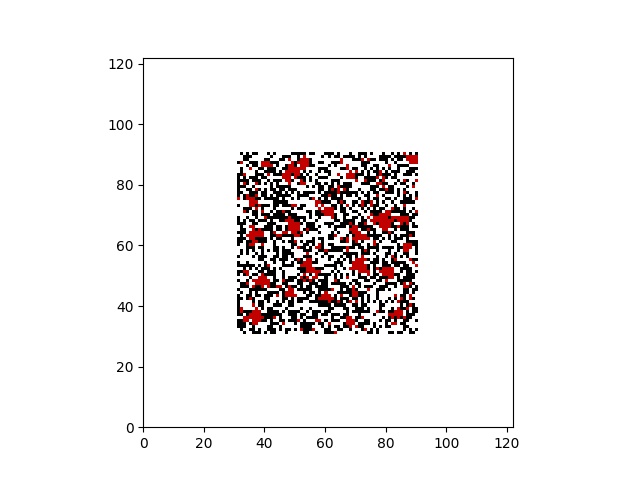

Supplement: Supplementary file 10 — Supplementary Software 1 [file 41467_2021_21614_MOESM10_ESM.zip › SupplementaryCode/DemoResult/Volume_60/seq00416.jpg]

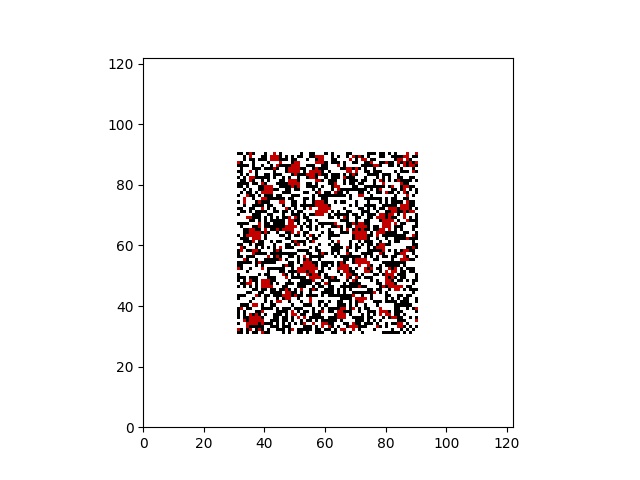

Supplement: Supplementary file 10 — Supplementary Software 1 [file 41467_2021_21614_MOESM10_ESM.zip › SupplementaryCode/DemoResult/Volume_60/seq00166.jpg]

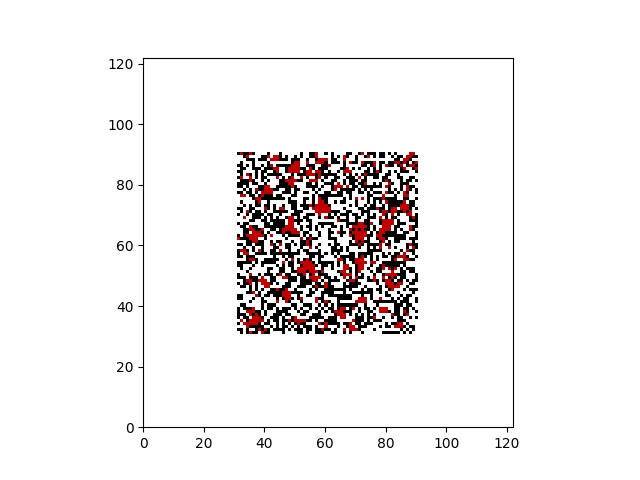

Supplement: Supplementary file 10 — Supplementary Software 1 [file 41467_2021_21614_MOESM10_ESM.zip › SupplementaryCode/DemoResult/Volume_60/seq00172.jpg]

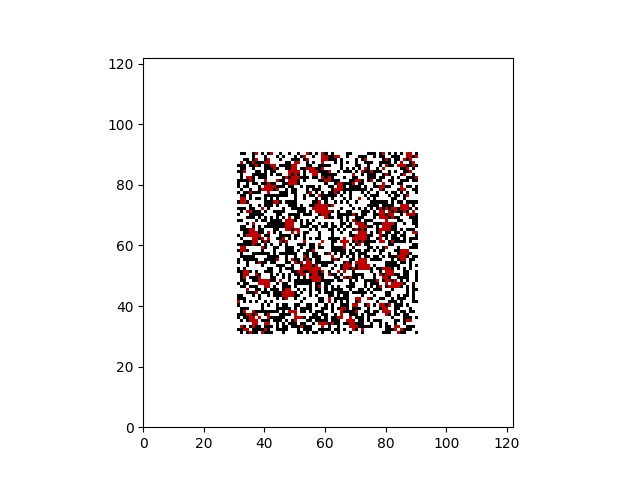

Supplement: Supplementary file 10 — Supplementary Software 1 [file 41467_2021_21614_MOESM10_ESM.zip › SupplementaryCode/DemoResult/Volume_60/seq00199.jpg]

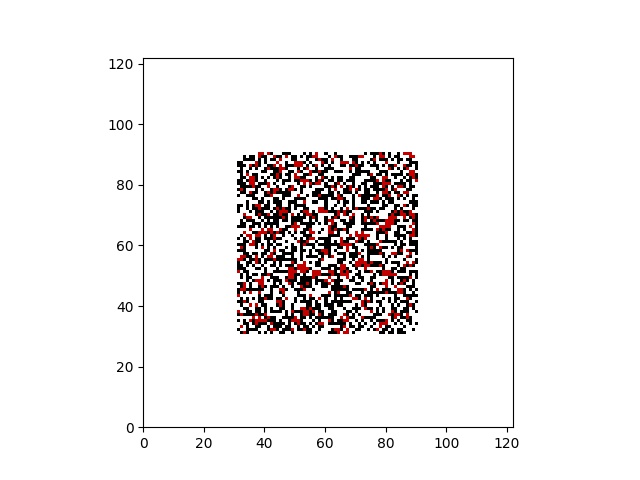

Supplement: Supplementary file 10 — Supplementary Software 1 [file 41467_2021_21614_MOESM10_ESM.zip › SupplementaryCode/DemoResult/Volume_60/seq00012.jpg]

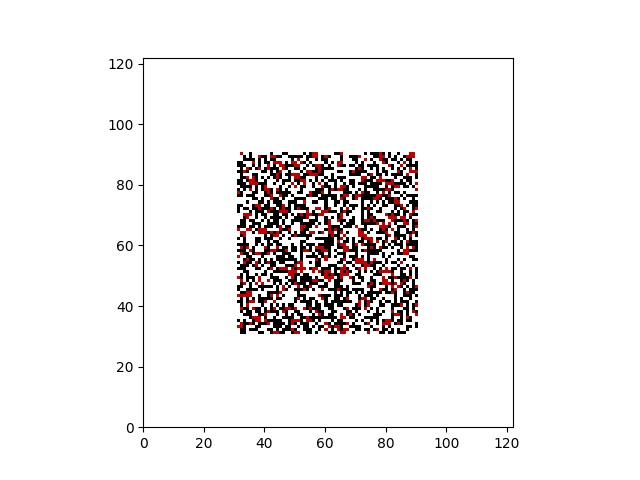

Supplement: Supplementary file 10 — Supplementary Software 1 [file 41467_2021_21614_MOESM10_ESM.zip › SupplementaryCode/DemoResult/Volume_60/seq00006.jpg]

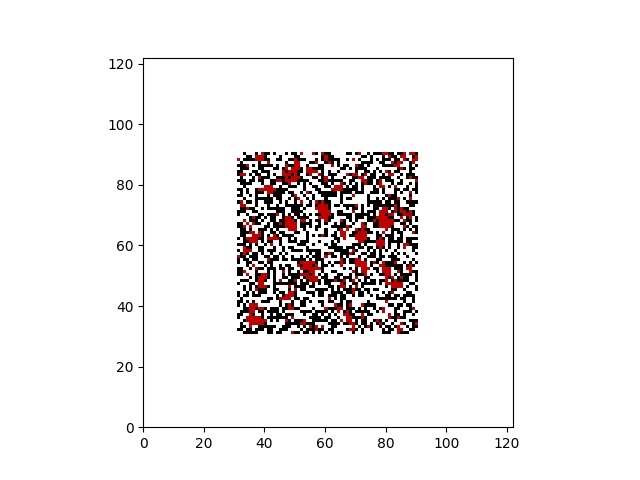

Supplement: Supplementary file 10 — Supplementary Software 1 [file 41467_2021_21614_MOESM10_ESM.zip › SupplementaryCode/DemoResult/Volume_60/seq00238.jpg]

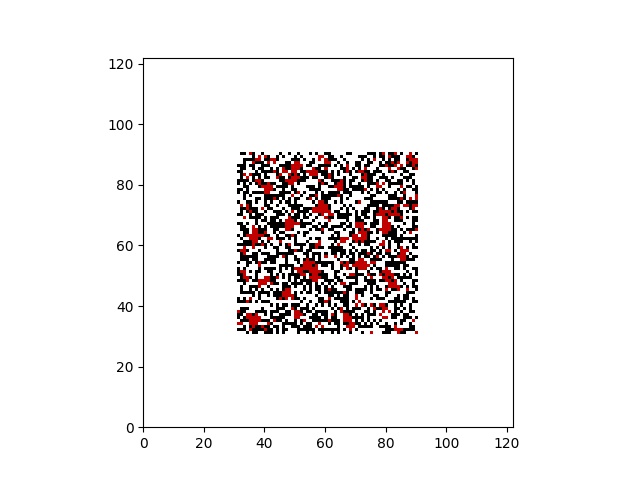

Supplement: Supplementary file 10 — Supplementary Software 1 [file 41467_2021_21614_MOESM10_ESM.zip › SupplementaryCode/DemoResult/Volume_60/seq00210.jpg]

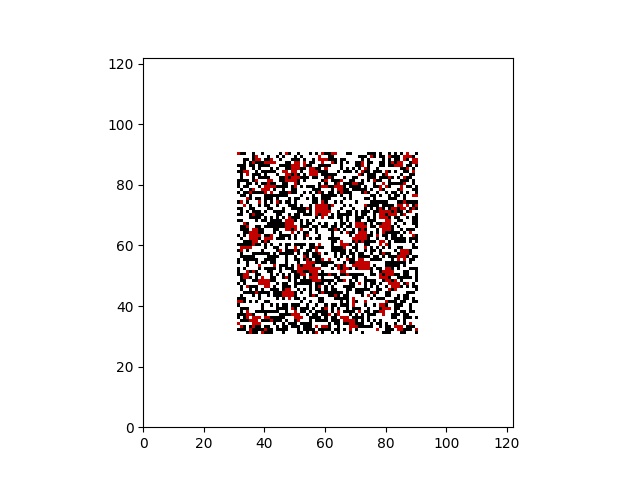

Supplement: Supplementary file 10 — Supplementary Software 1 [file 41467_2021_21614_MOESM10_ESM.zip › SupplementaryCode/DemoResult/Volume_60/seq00204.jpg]

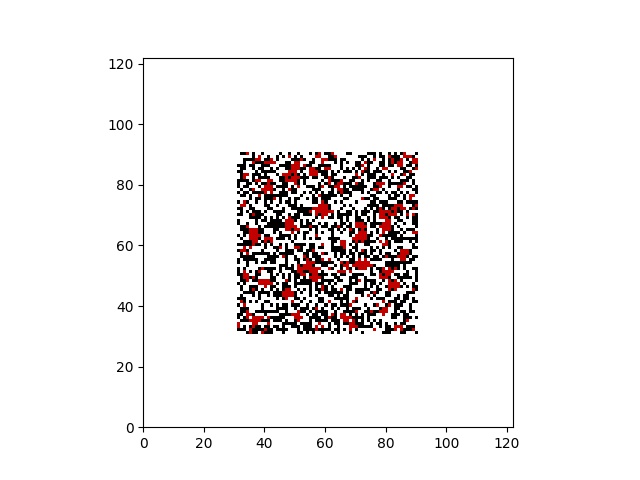

Supplement: Supplementary file 10 — Supplementary Software 1 [file 41467_2021_21614_MOESM10_ESM.zip › SupplementaryCode/DemoResult/Volume_60/seq00205.jpg]

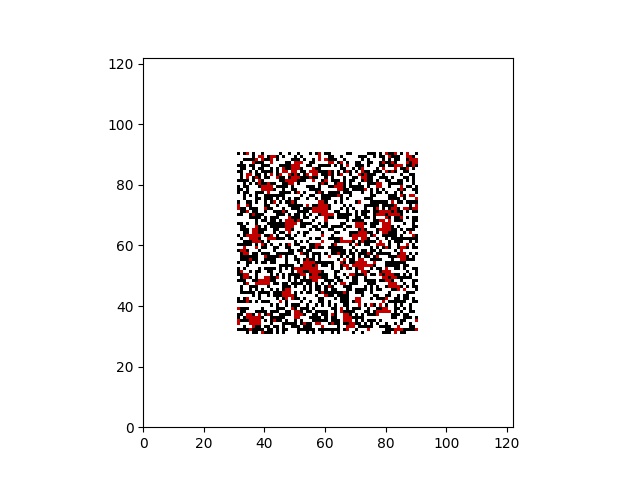

Supplement: Supplementary file 10 — Supplementary Software 1 [file 41467_2021_21614_MOESM10_ESM.zip › SupplementaryCode/DemoResult/Volume_60/seq00211.jpg]

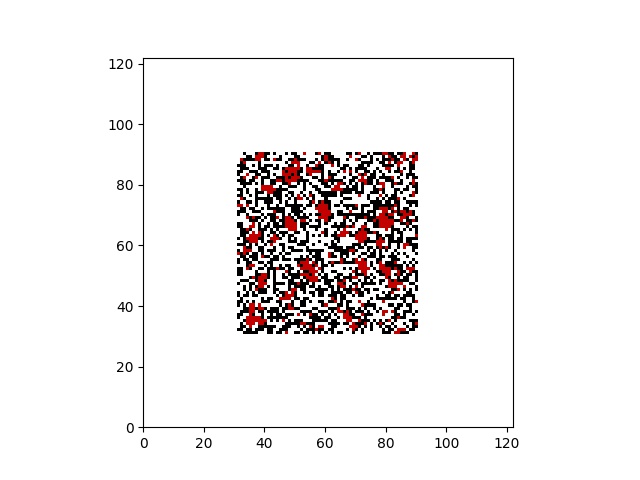

Supplement: Supplementary file 10 — Supplementary Software 1 [file 41467_2021_21614_MOESM10_ESM.zip › SupplementaryCode/DemoResult/Volume_60/seq00239.jpg]

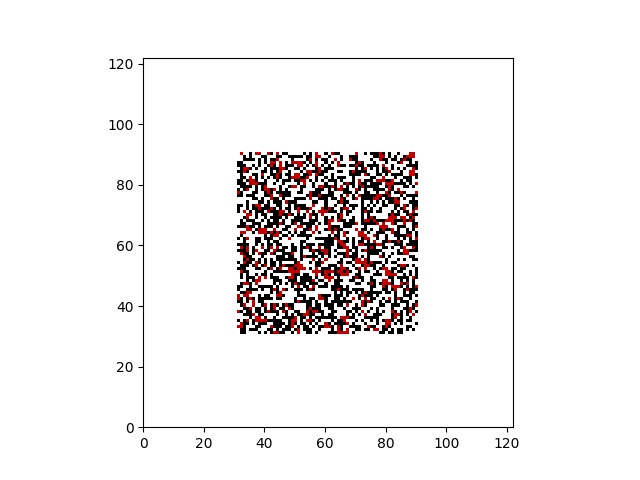

Supplement: Supplementary file 10 — Supplementary Software 1 [file 41467_2021_21614_MOESM10_ESM.zip › SupplementaryCode/DemoResult/Volume_60/seq00007.jpg]

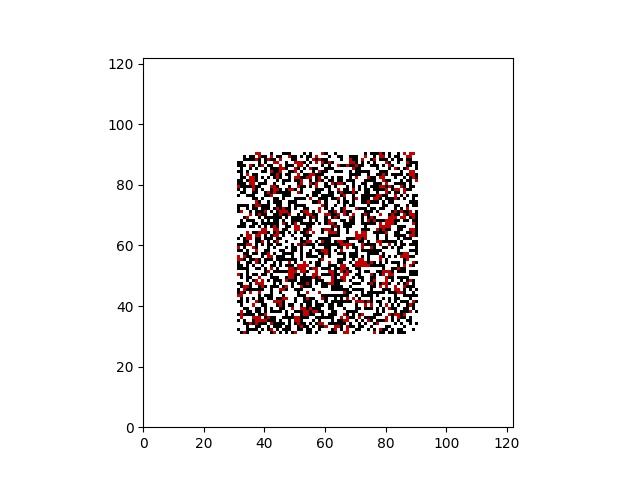

Supplement: Supplementary file 10 — Supplementary Software 1 [file 41467_2021_21614_MOESM10_ESM.zip › SupplementaryCode/DemoResult/Volume_60/seq00013.jpg]

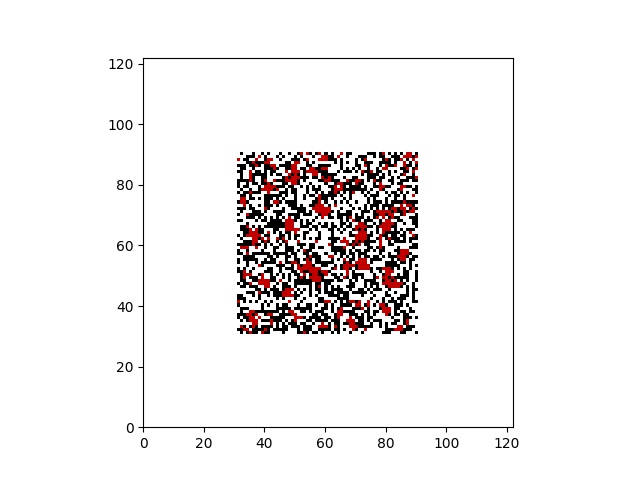

Supplement: Supplementary file 10 — Supplementary Software 1 [file 41467_2021_21614_MOESM10_ESM.zip › SupplementaryCode/DemoResult/Volume_60/seq00198.jpg]

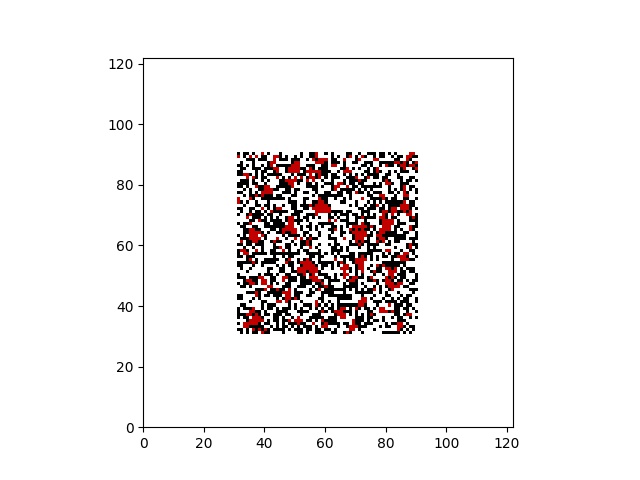

Supplement: Supplementary file 10 — Supplementary Software 1 [file 41467_2021_21614_MOESM10_ESM.zip › SupplementaryCode/DemoResult/Volume_60/seq00173.jpg]

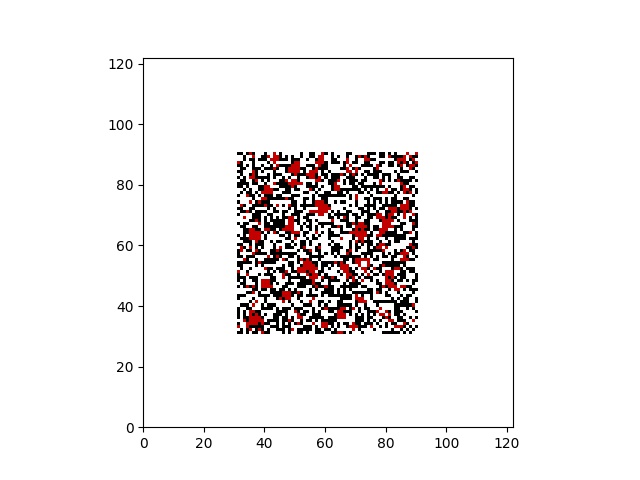

Supplement: Supplementary file 10 — Supplementary Software 1 [file 41467_2021_21614_MOESM10_ESM.zip › SupplementaryCode/DemoResult/Volume_60/seq00167.jpg]

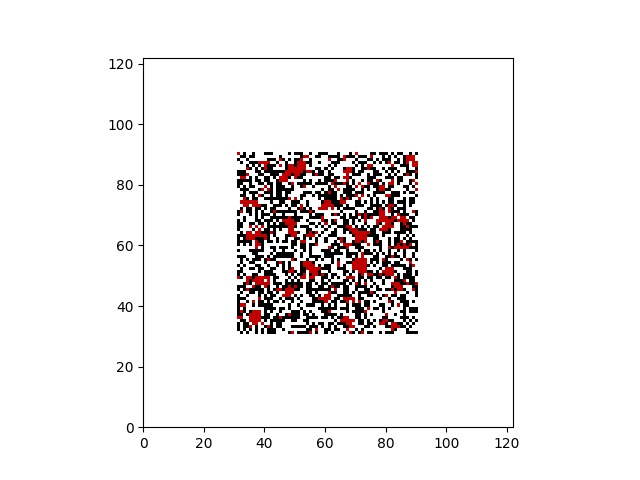

Supplement: Supplementary file 10 — Supplementary Software 1 [file 41467_2021_21614_MOESM10_ESM.zip › SupplementaryCode/DemoResult/Volume_60/seq00371.jpg]

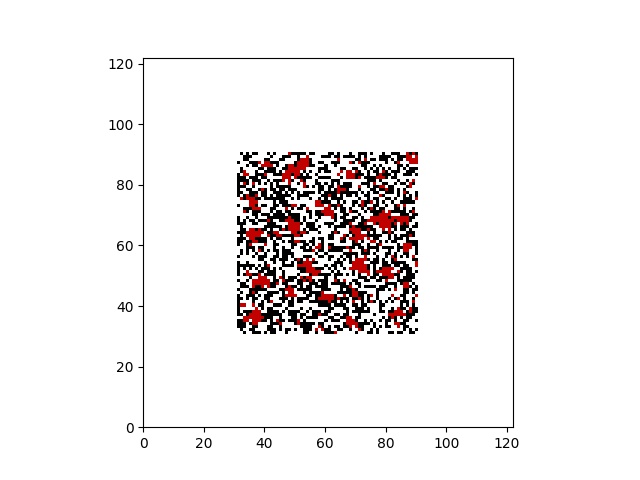

Supplement: Supplementary file 10 — Supplementary Software 1 [file 41467_2021_21614_MOESM10_ESM.zip › SupplementaryCode/DemoResult/Volume_60/seq00417.jpg]

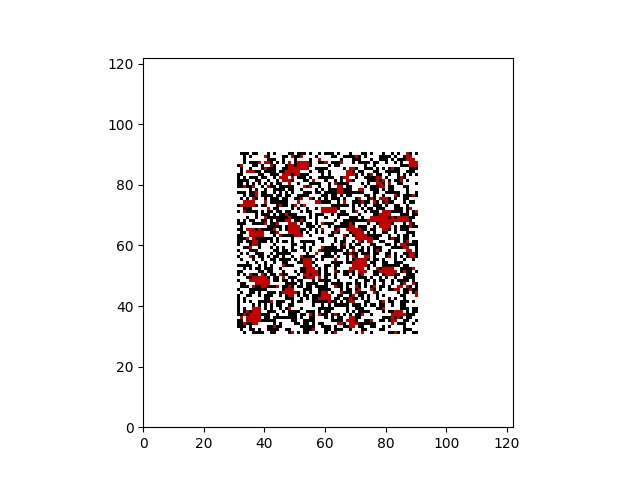

Supplement: Supplementary file 10 — Supplementary Software 1 [file 41467_2021_21614_MOESM10_ESM.zip › SupplementaryCode/DemoResult/Volume_60/seq00403.jpg]

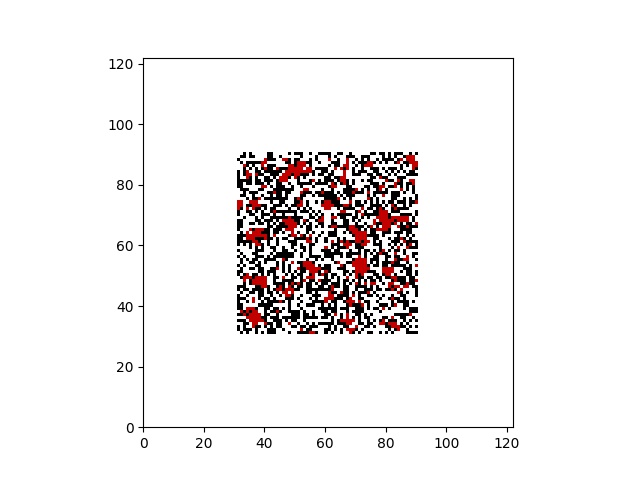

Supplement: Supplementary file 10 — Supplementary Software 1 [file 41467_2021_21614_MOESM10_ESM.zip › SupplementaryCode/DemoResult/Volume_60/seq00365.jpg]

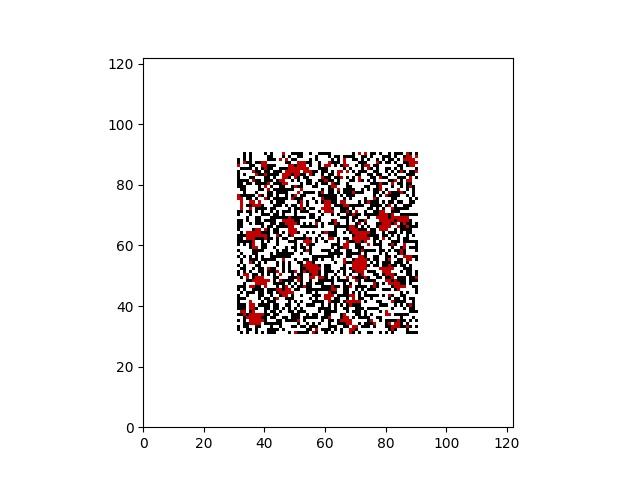

Supplement: Supplementary file 10 — Supplementary Software 1 [file 41467_2021_21614_MOESM10_ESM.zip › SupplementaryCode/DemoResult/Volume_60/seq00359.jpg]

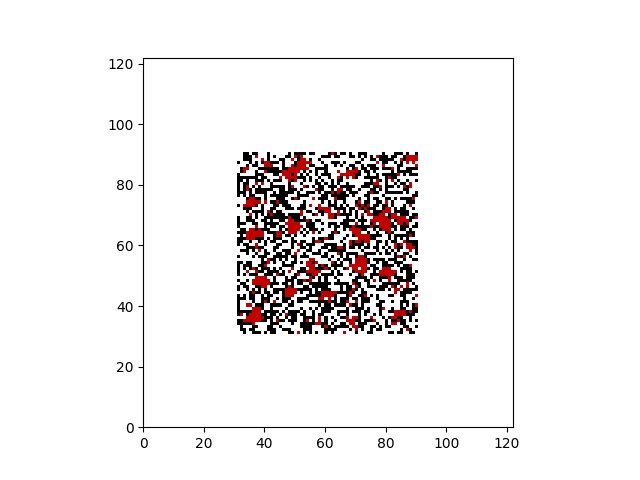

Supplement: Supplementary file 10 — Supplementary Software 1 [file 41467_2021_21614_MOESM10_ESM.zip › SupplementaryCode/DemoResult/Volume_60/seq00429.jpg]

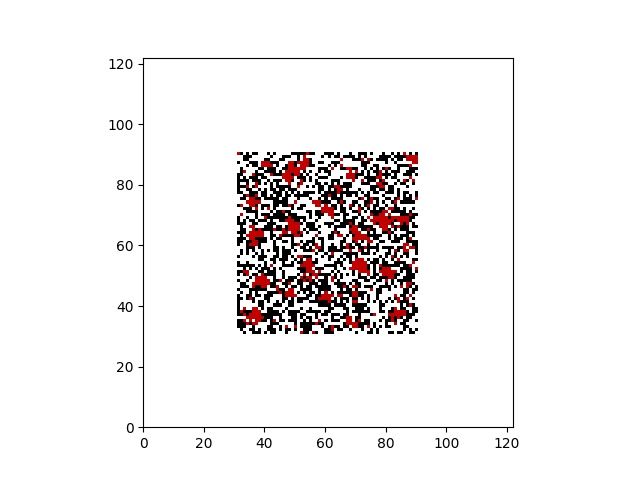

Supplement: Supplementary file 10 — Supplementary Software 1 [file 41467_2021_21614_MOESM10_ESM.zip › SupplementaryCode/DemoResult/Volume_60/seq00415.jpg]

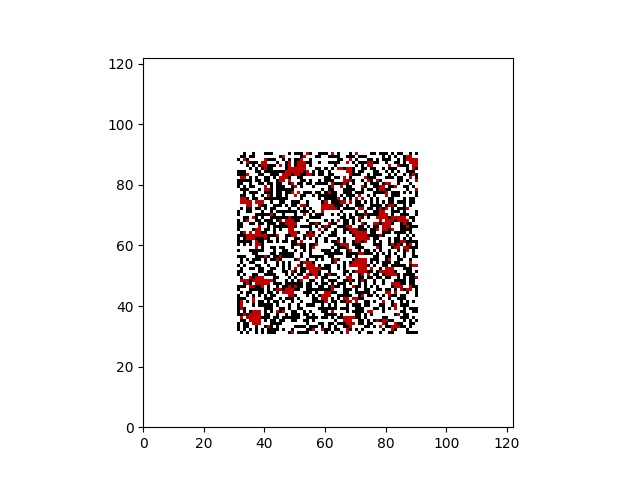

Supplement: Supplementary file 10 — Supplementary Software 1 [file 41467_2021_21614_MOESM10_ESM.zip › SupplementaryCode/DemoResult/Volume_60/seq00373.jpg]

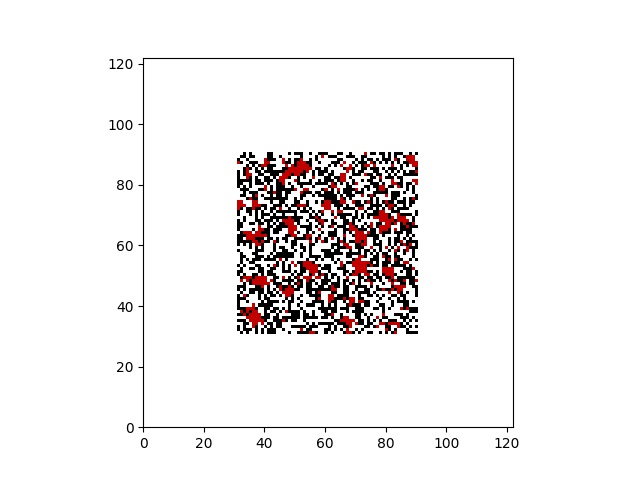

Supplement: Supplementary file 10 — Supplementary Software 1 [file 41467_2021_21614_MOESM10_ESM.zip › SupplementaryCode/DemoResult/Volume_60/seq00367.jpg]
