# Supplementary figures and images for: Cells recognize osmotic stress through liquid–liquid phase separation lubricated with poly(ADP-ribose) (part 1 of 2)
Source: Nat Commun. 2021 Mar 1;12:1353. doi: 10.1038/s41467-021-21614-5 (PMC7921423; doi:10.1038/s41467-021-21614-5)

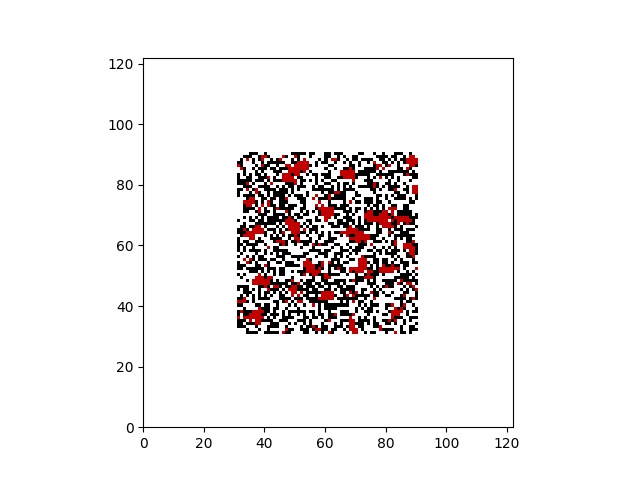

Supplement: Supplementary file 10 — Supplementary Software 1 [file 41467_2021_21614_MOESM10_ESM.zip › SupplementaryCode/DemoResult/Volume_60/seq00473.jpg]

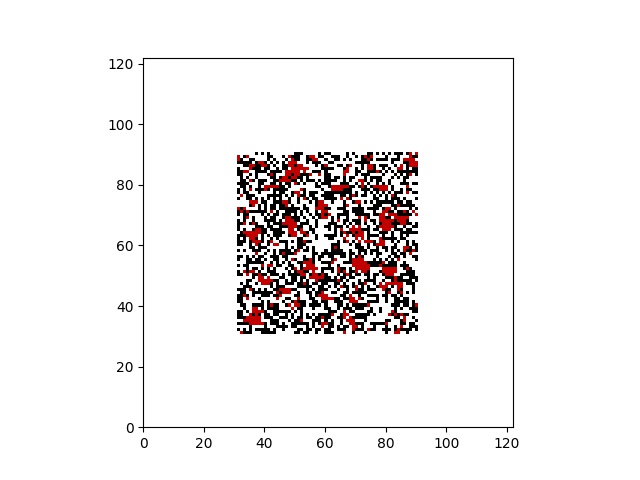

Supplement: Supplementary file 10 — Supplementary Software 1 [file 41467_2021_21614_MOESM10_ESM.zip › SupplementaryCode/DemoResult/Volume_60/seq00315.jpg]

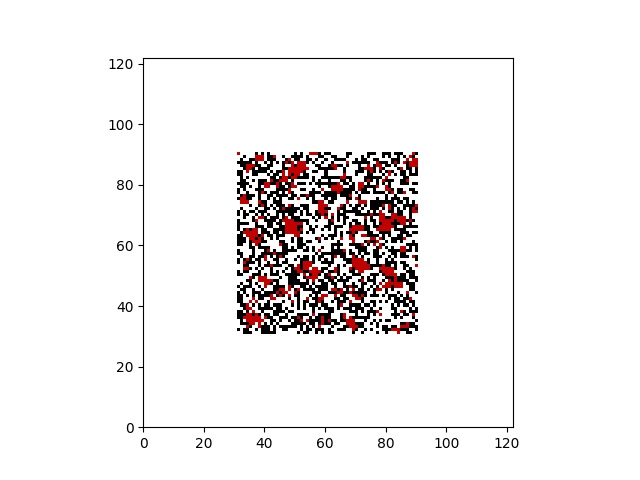

Supplement: Supplementary file 10 — Supplementary Software 1 [file 41467_2021_21614_MOESM10_ESM.zip › SupplementaryCode/DemoResult/Volume_60/seq00301.jpg]

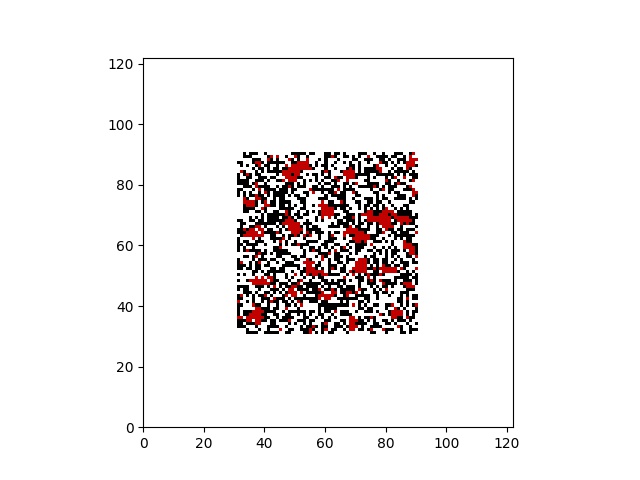

Supplement: Supplementary file 10 — Supplementary Software 1 [file 41467_2021_21614_MOESM10_ESM.zip › SupplementaryCode/DemoResult/Volume_60/seq00467.jpg]

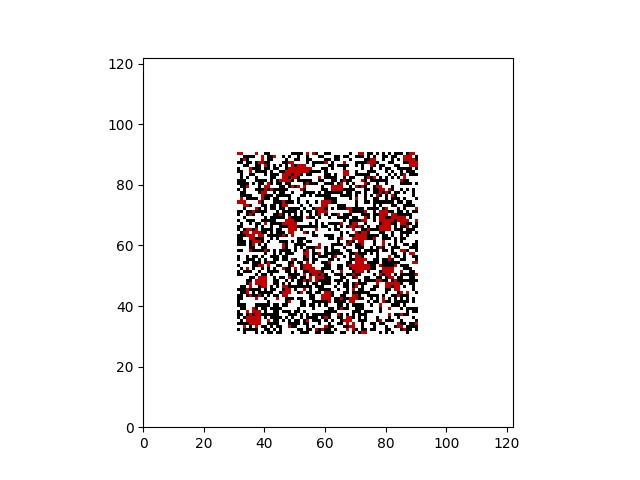

Supplement: Supplementary file 10 — Supplementary Software 1 [file 41467_2021_21614_MOESM10_ESM.zip › SupplementaryCode/DemoResult/Volume_60/seq00329.jpg]

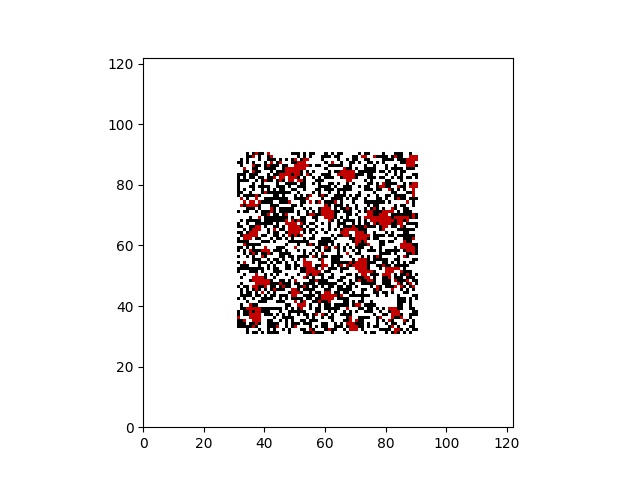

Supplement: Supplementary file 10 — Supplementary Software 1 [file 41467_2021_21614_MOESM10_ESM.zip › SupplementaryCode/DemoResult/Volume_60/seq00498.jpg]

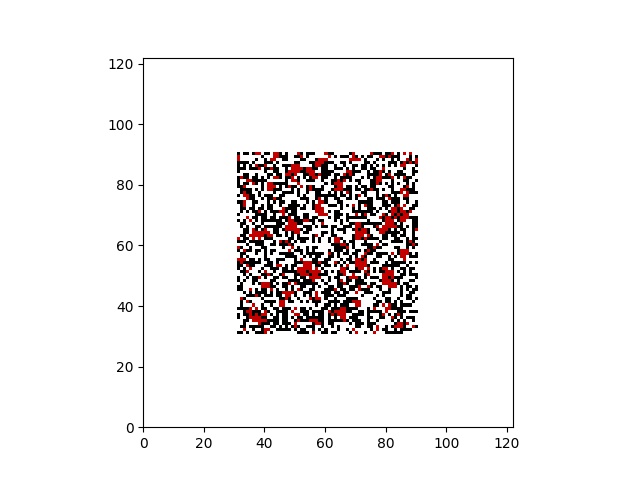

Supplement: Supplementary file 10 — Supplementary Software 1 [file 41467_2021_21614_MOESM10_ESM.zip › SupplementaryCode/DemoResult/Volume_60/seq00117.jpg]

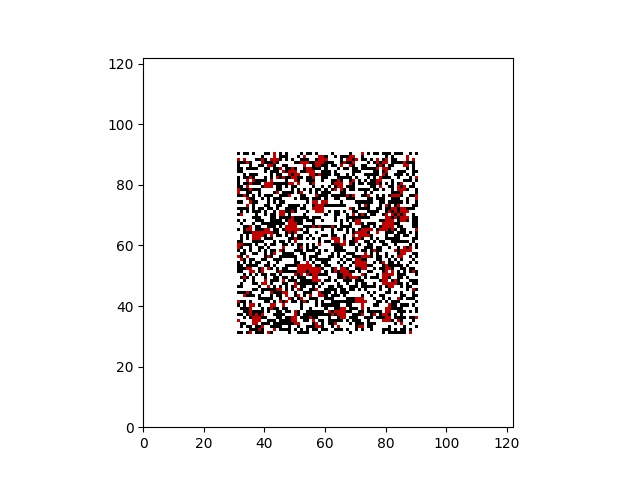

Supplement: Supplementary file 10 — Supplementary Software 1 [file 41467_2021_21614_MOESM10_ESM.zip › SupplementaryCode/DemoResult/Volume_60/seq00103.jpg]

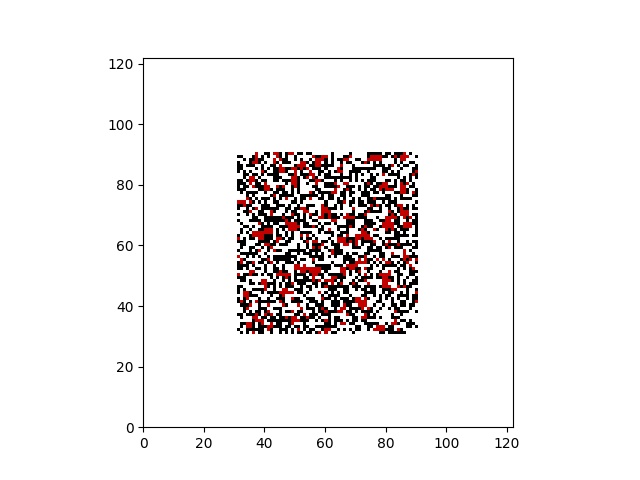

Supplement: Supplementary file 10 — Supplementary Software 1 [file 41467_2021_21614_MOESM10_ESM.zip › SupplementaryCode/DemoResult/Volume_60/seq00063.jpg]

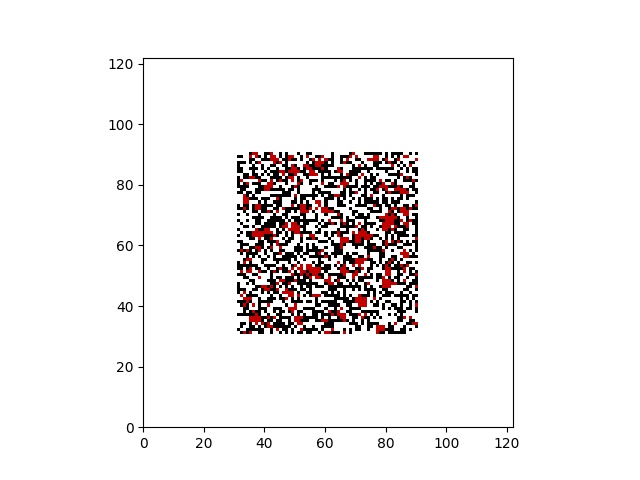

Supplement: Supplementary file 10 — Supplementary Software 1 [file 41467_2021_21614_MOESM10_ESM.zip › SupplementaryCode/DemoResult/Volume_60/seq00077.jpg]

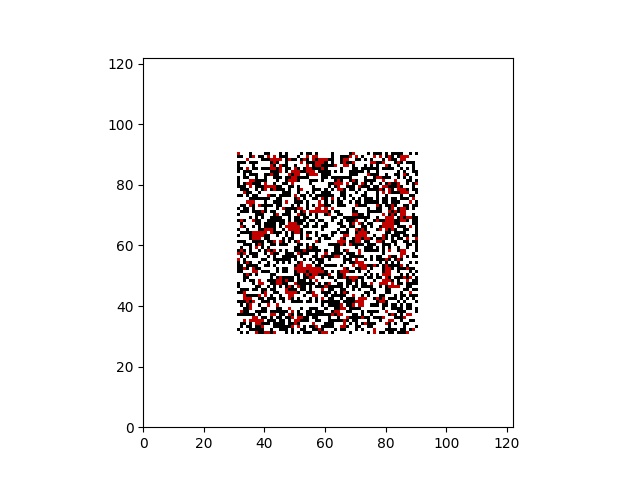

Supplement: Supplementary file 10 — Supplementary Software 1 [file 41467_2021_21614_MOESM10_ESM.zip › SupplementaryCode/DemoResult/Volume_60/seq00088.jpg]

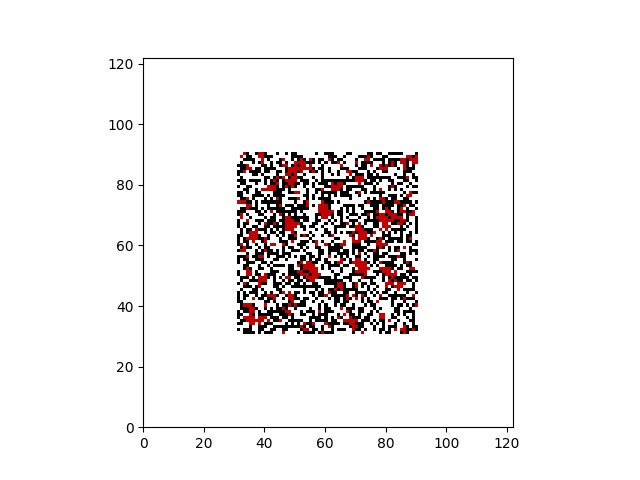

Supplement: Supplementary file 10 — Supplementary Software 1 [file 41467_2021_21614_MOESM10_ESM.zip › SupplementaryCode/DemoResult/Volume_60/seq00261.jpg]

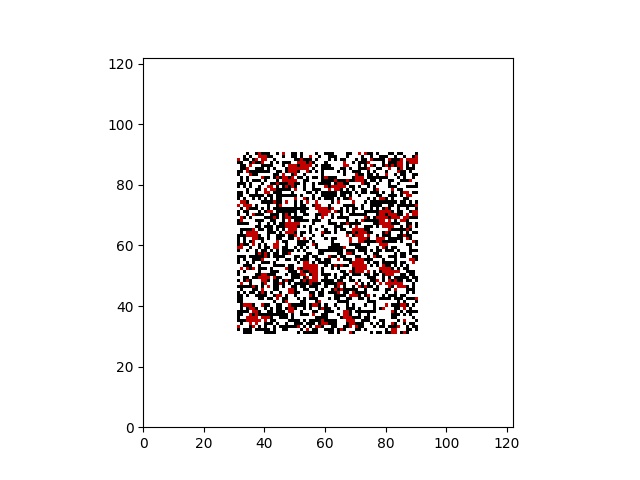

Supplement: Supplementary file 10 — Supplementary Software 1 [file 41467_2021_21614_MOESM10_ESM.zip › SupplementaryCode/DemoResult/Volume_60/seq00275.jpg]

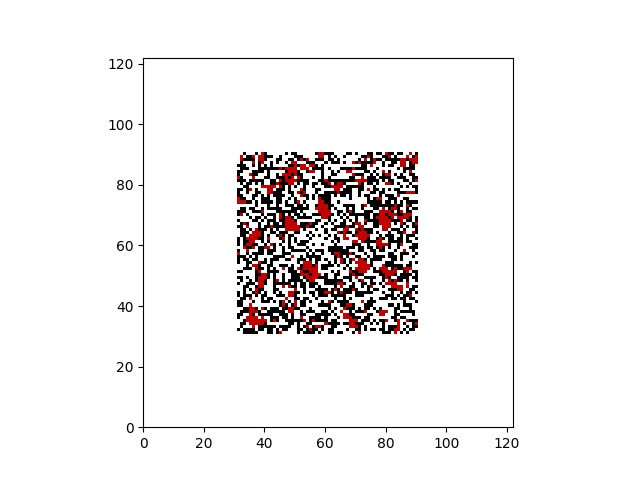

Supplement: Supplementary file 10 — Supplementary Software 1 [file 41467_2021_21614_MOESM10_ESM.zip › SupplementaryCode/DemoResult/Volume_60/seq00249.jpg]

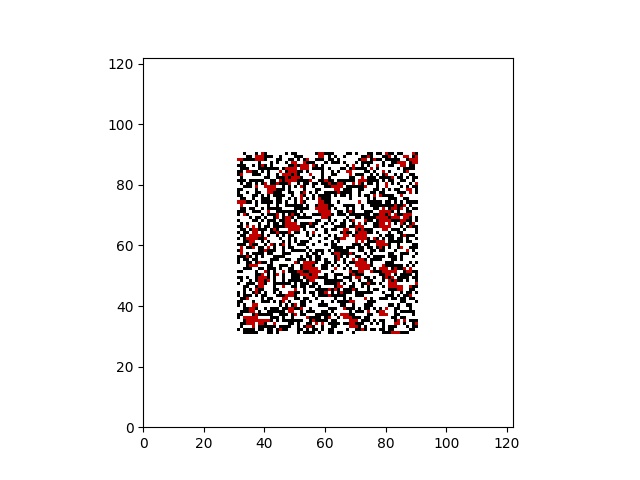

Supplement: Supplementary file 10 — Supplementary Software 1 [file 41467_2021_21614_MOESM10_ESM.zip › SupplementaryCode/DemoResult/Volume_60/seq00248.jpg]

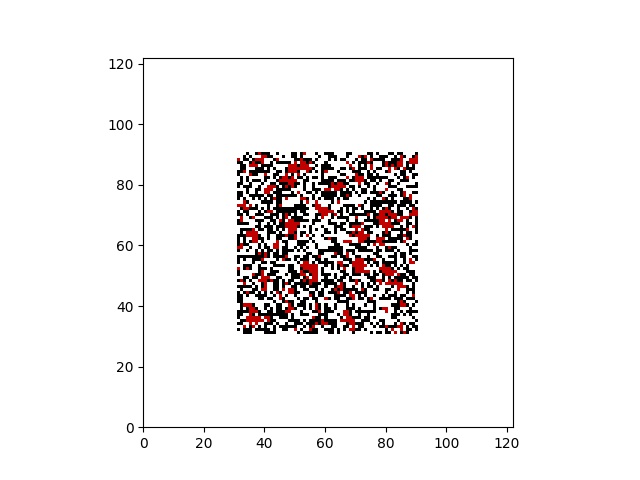

Supplement: Supplementary file 10 — Supplementary Software 1 [file 41467_2021_21614_MOESM10_ESM.zip › SupplementaryCode/DemoResult/Volume_60/seq00274.jpg]

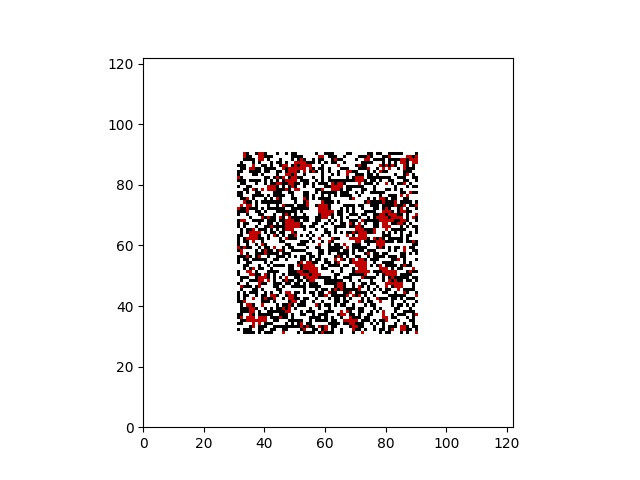

Supplement: Supplementary file 10 — Supplementary Software 1 [file 41467_2021_21614_MOESM10_ESM.zip › SupplementaryCode/DemoResult/Volume_60/seq00260.jpg]

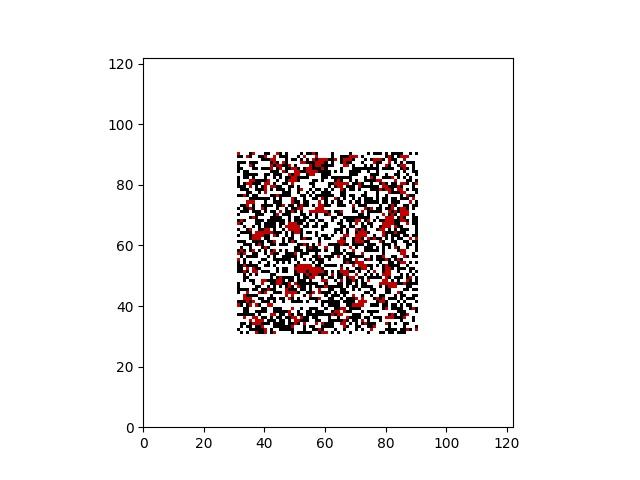

Supplement: Supplementary file 10 — Supplementary Software 1 [file 41467_2021_21614_MOESM10_ESM.zip › SupplementaryCode/DemoResult/Volume_60/seq00089.jpg]

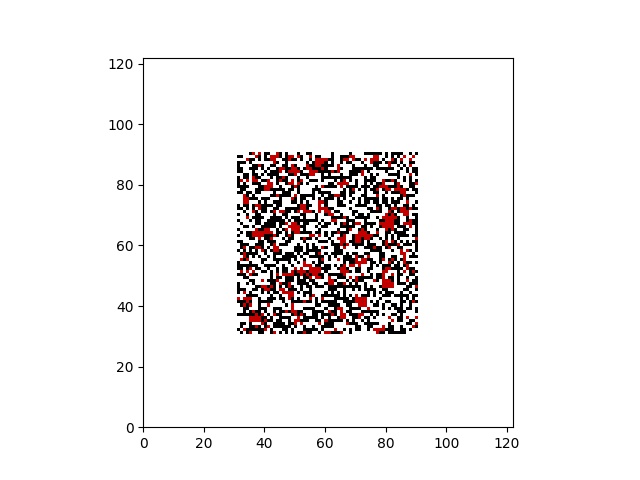

Supplement: Supplementary file 10 — Supplementary Software 1 [file 41467_2021_21614_MOESM10_ESM.zip › SupplementaryCode/DemoResult/Volume_60/seq00076.jpg]

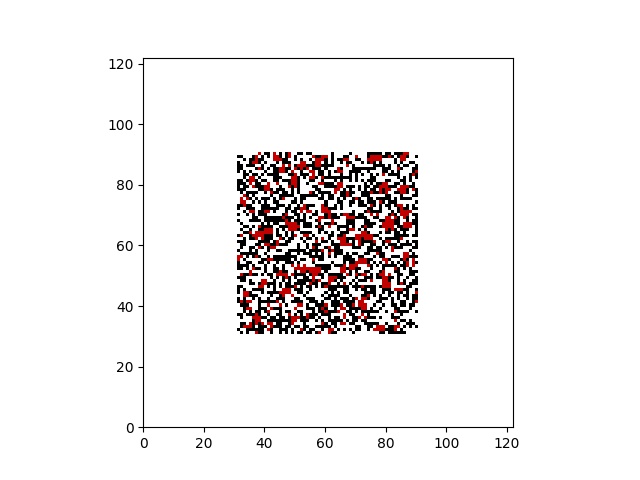

Supplement: Supplementary file 10 — Supplementary Software 1 [file 41467_2021_21614_MOESM10_ESM.zip › SupplementaryCode/DemoResult/Volume_60/seq00062.jpg]

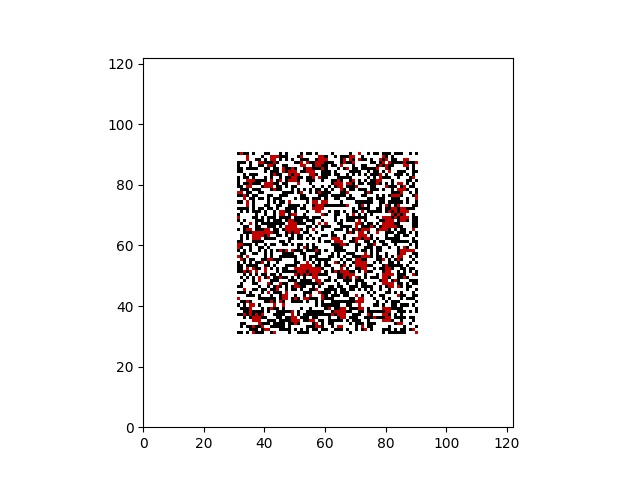

Supplement: Supplementary file 10 — Supplementary Software 1 [file 41467_2021_21614_MOESM10_ESM.zip › SupplementaryCode/DemoResult/Volume_60/seq00102.jpg]

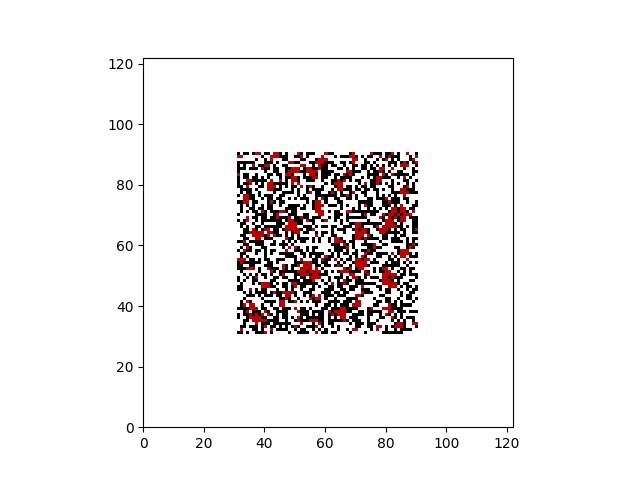

Supplement: Supplementary file 10 — Supplementary Software 1 [file 41467_2021_21614_MOESM10_ESM.zip › SupplementaryCode/DemoResult/Volume_60/seq00116.jpg]

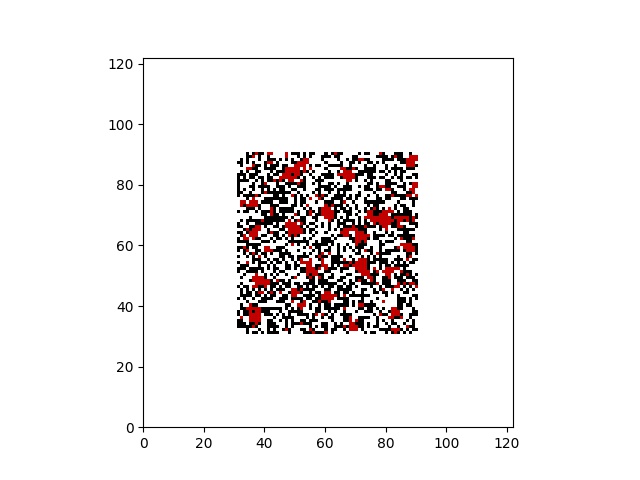

Supplement: Supplementary file 10 — Supplementary Software 1 [file 41467_2021_21614_MOESM10_ESM.zip › SupplementaryCode/DemoResult/Volume_60/seq00499.jpg]

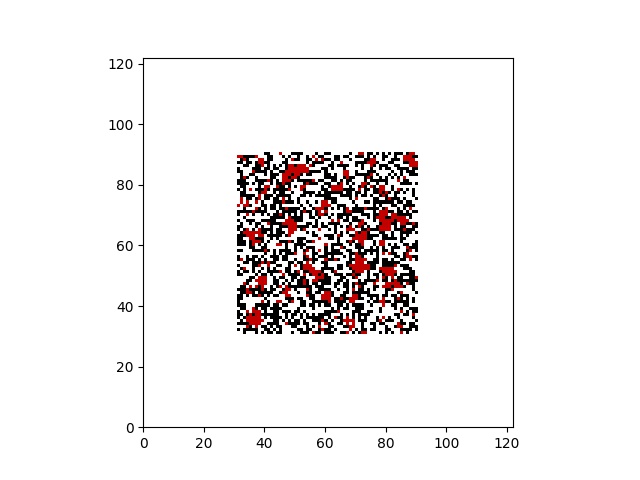

Supplement: Supplementary file 10 — Supplementary Software 1 [file 41467_2021_21614_MOESM10_ESM.zip › SupplementaryCode/DemoResult/Volume_60/seq00328.jpg]

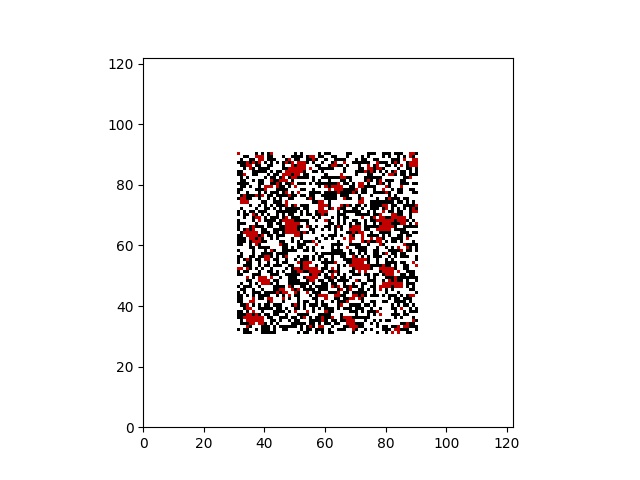

Supplement: Supplementary file 10 — Supplementary Software 1 [file 41467_2021_21614_MOESM10_ESM.zip › SupplementaryCode/DemoResult/Volume_60/seq00300.jpg]

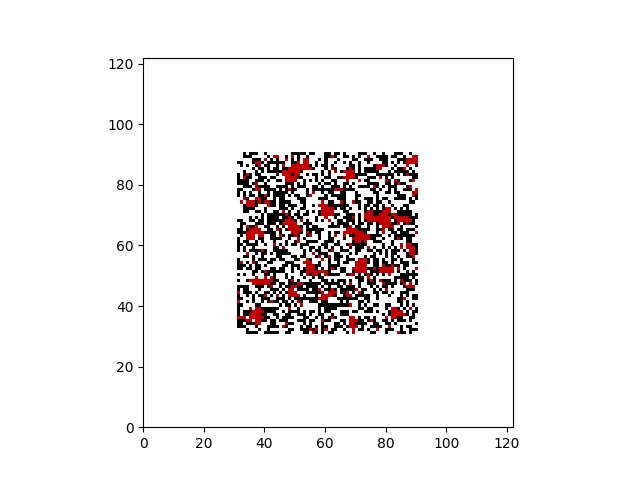

Supplement: Supplementary file 10 — Supplementary Software 1 [file 41467_2021_21614_MOESM10_ESM.zip › SupplementaryCode/DemoResult/Volume_60/seq00466.jpg]

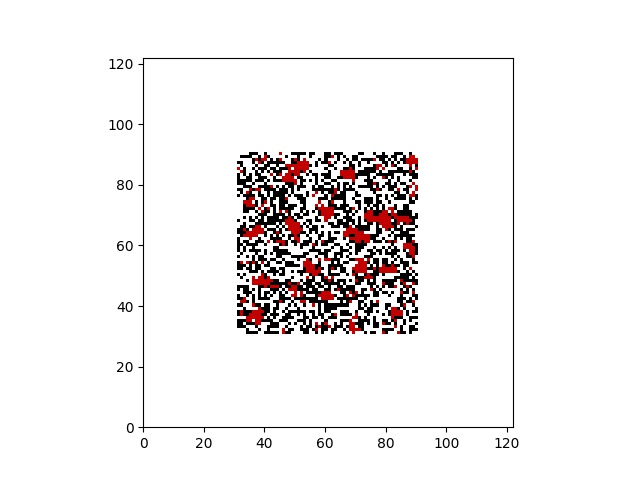

Supplement: Supplementary file 10 — Supplementary Software 1 [file 41467_2021_21614_MOESM10_ESM.zip › SupplementaryCode/DemoResult/Volume_60/seq00472.jpg]

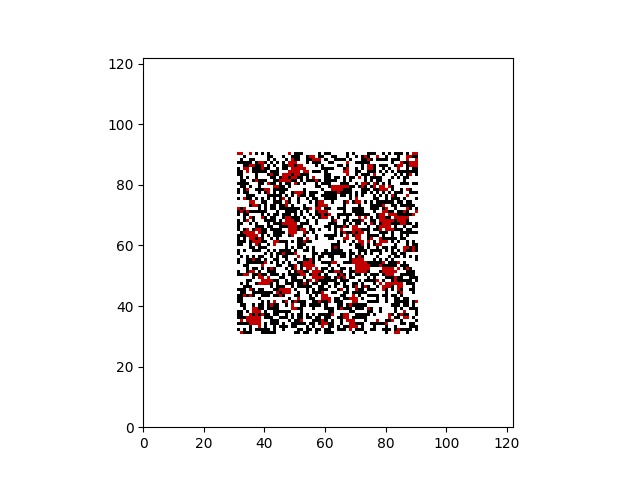

Supplement: Supplementary file 10 — Supplementary Software 1 [file 41467_2021_21614_MOESM10_ESM.zip › SupplementaryCode/DemoResult/Volume_60/seq00314.jpg]

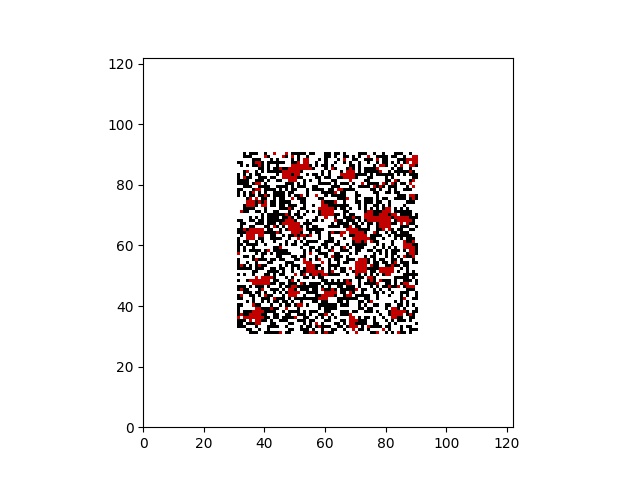

Supplement: Supplementary file 10 — Supplementary Software 1 [file 41467_2021_21614_MOESM10_ESM.zip › SupplementaryCode/DemoResult/Volume_60/seq00464.jpg]

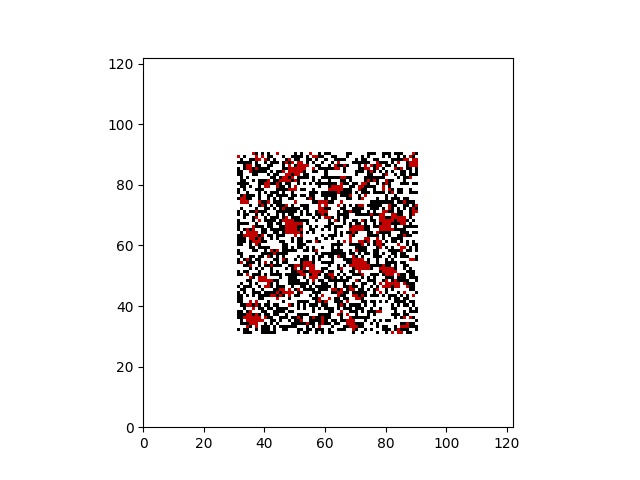

Supplement: Supplementary file 10 — Supplementary Software 1 [file 41467_2021_21614_MOESM10_ESM.zip › SupplementaryCode/DemoResult/Volume_60/seq00302.jpg]

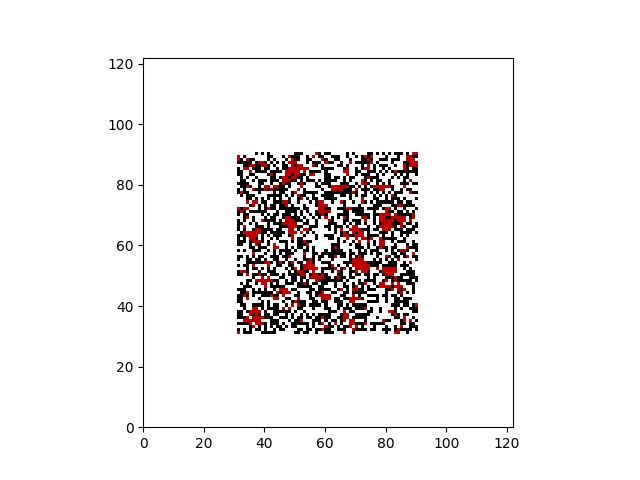

Supplement: Supplementary file 10 — Supplementary Software 1 [file 41467_2021_21614_MOESM10_ESM.zip › SupplementaryCode/DemoResult/Volume_60/seq00316.jpg]

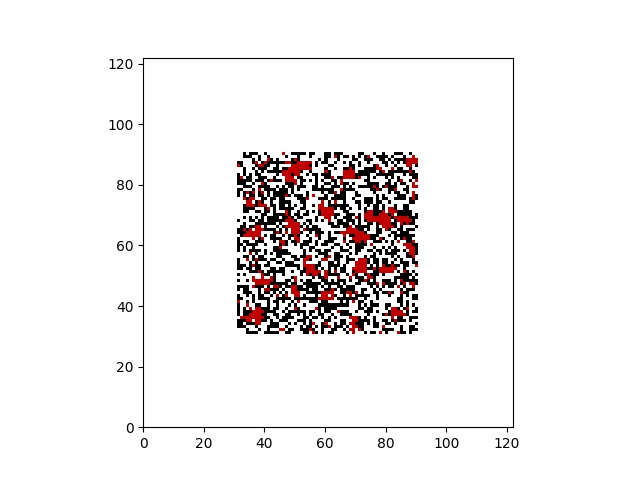

Supplement: Supplementary file 10 — Supplementary Software 1 [file 41467_2021_21614_MOESM10_ESM.zip › SupplementaryCode/DemoResult/Volume_60/seq00470.jpg]

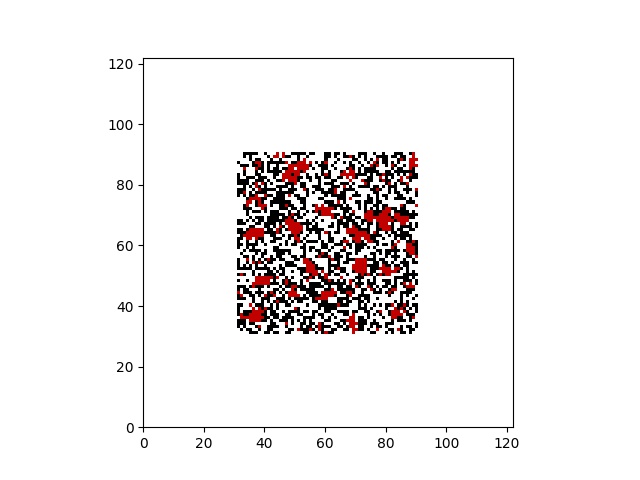

Supplement: Supplementary file 10 — Supplementary Software 1 [file 41467_2021_21614_MOESM10_ESM.zip › SupplementaryCode/DemoResult/Volume_60/seq00458.jpg]

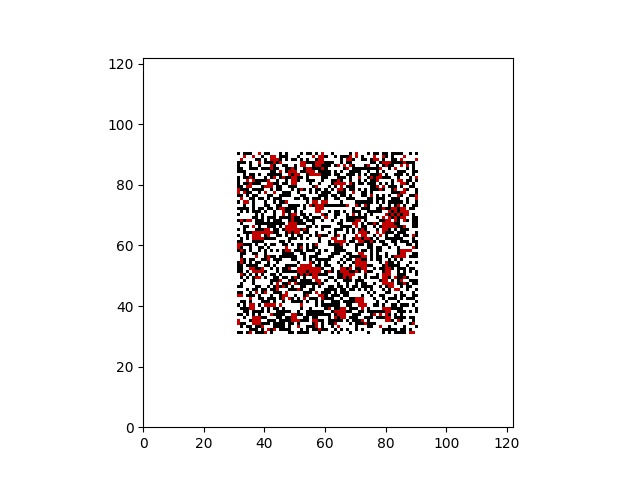

Supplement: Supplementary file 10 — Supplementary Software 1 [file 41467_2021_21614_MOESM10_ESM.zip › SupplementaryCode/DemoResult/Volume_60/seq00100.jpg]

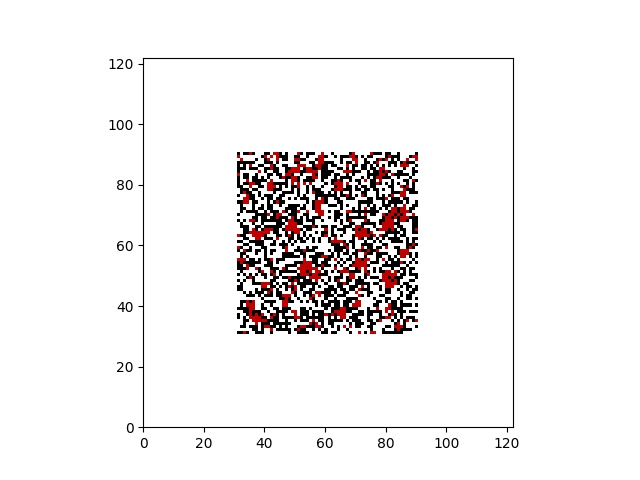

Supplement: Supplementary file 10 — Supplementary Software 1 [file 41467_2021_21614_MOESM10_ESM.zip › SupplementaryCode/DemoResult/Volume_60/seq00114.jpg]

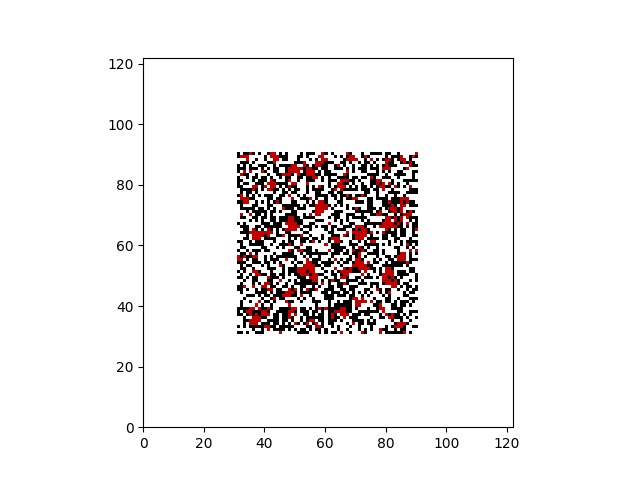

Supplement: Supplementary file 10 — Supplementary Software 1 [file 41467_2021_21614_MOESM10_ESM.zip › SupplementaryCode/DemoResult/Volume_60/seq00128.jpg]

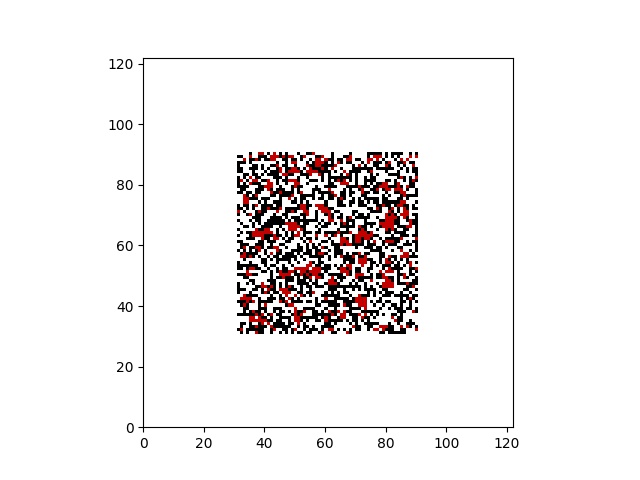

Supplement: Supplementary file 10 — Supplementary Software 1 [file 41467_2021_21614_MOESM10_ESM.zip › SupplementaryCode/DemoResult/Volume_60/seq00074.jpg]

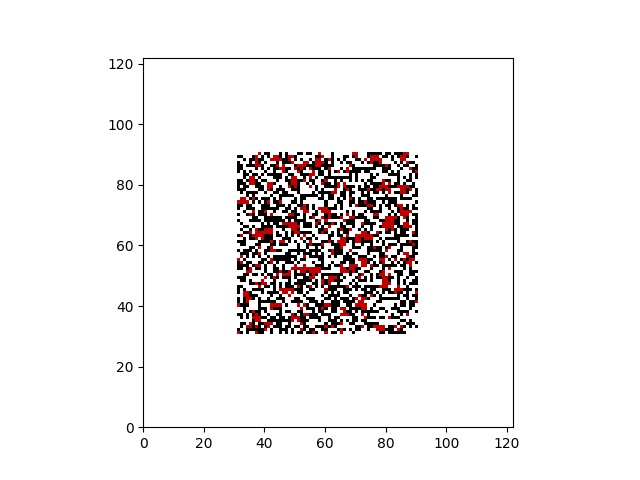

Supplement: Supplementary file 10 — Supplementary Software 1 [file 41467_2021_21614_MOESM10_ESM.zip › SupplementaryCode/DemoResult/Volume_60/seq00060.jpg]

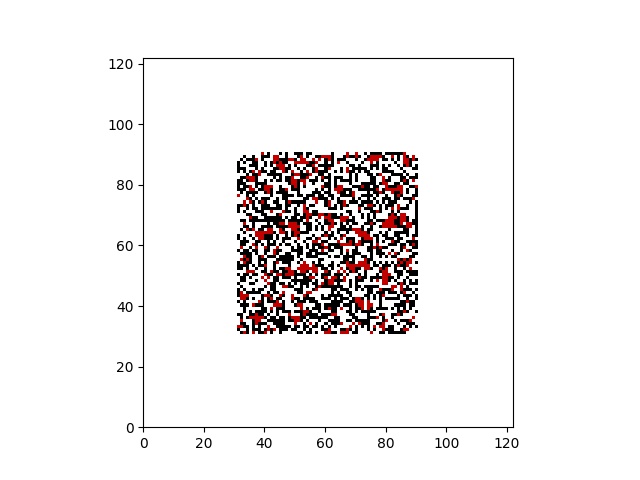

Supplement: Supplementary file 10 — Supplementary Software 1 [file 41467_2021_21614_MOESM10_ESM.zip › SupplementaryCode/DemoResult/Volume_60/seq00048.jpg]

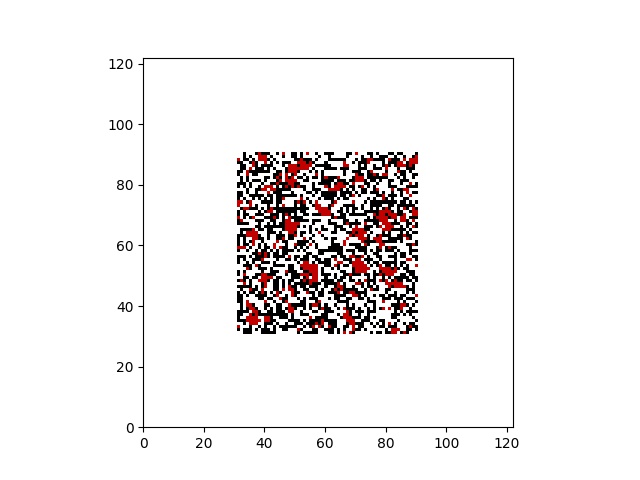

Supplement: Supplementary file 10 — Supplementary Software 1 [file 41467_2021_21614_MOESM10_ESM.zip › SupplementaryCode/DemoResult/Volume_60/seq00276.jpg]

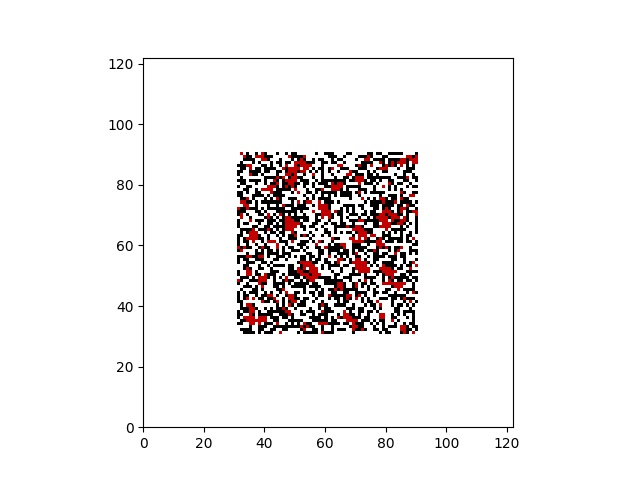

Supplement: Supplementary file 10 — Supplementary Software 1 [file 41467_2021_21614_MOESM10_ESM.zip › SupplementaryCode/DemoResult/Volume_60/seq00262.jpg]

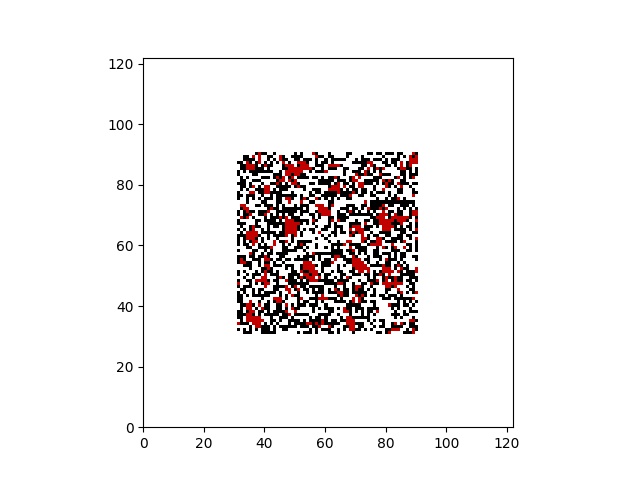

Supplement: Supplementary file 10 — Supplementary Software 1 [file 41467_2021_21614_MOESM10_ESM.zip › SupplementaryCode/DemoResult/Volume_60/seq00289.jpg]

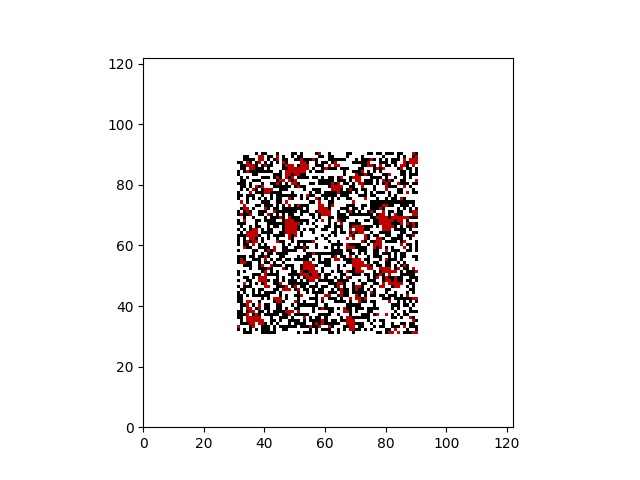

Supplement: Supplementary file 10 — Supplementary Software 1 [file 41467_2021_21614_MOESM10_ESM.zip › SupplementaryCode/DemoResult/Volume_60/seq00288.jpg]

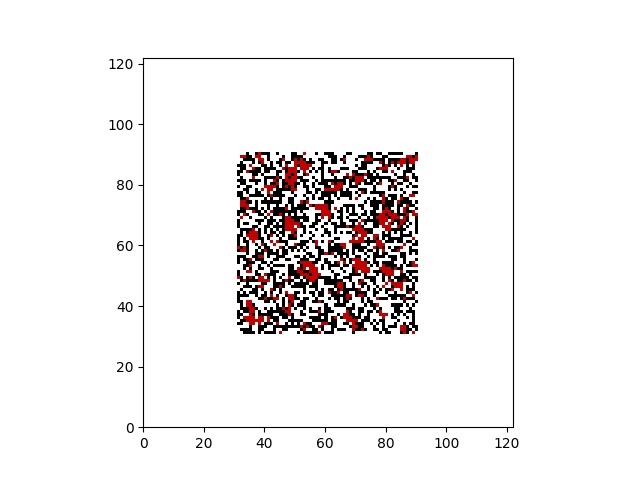

Supplement: Supplementary file 10 — Supplementary Software 1 [file 41467_2021_21614_MOESM10_ESM.zip › SupplementaryCode/DemoResult/Volume_60/seq00263.jpg]

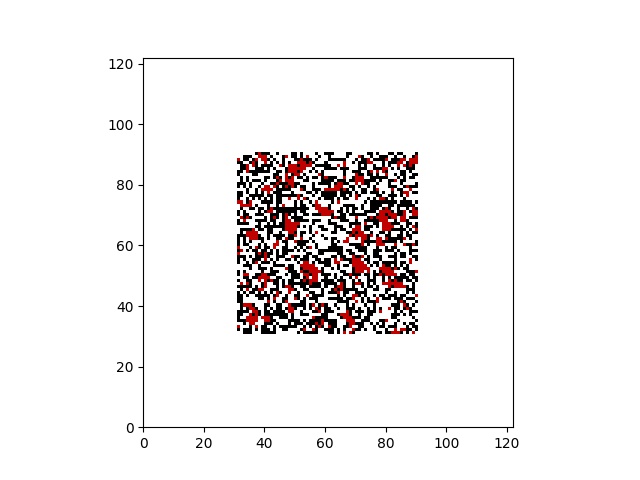

Supplement: Supplementary file 10 — Supplementary Software 1 [file 41467_2021_21614_MOESM10_ESM.zip › SupplementaryCode/DemoResult/Volume_60/seq00277.jpg]

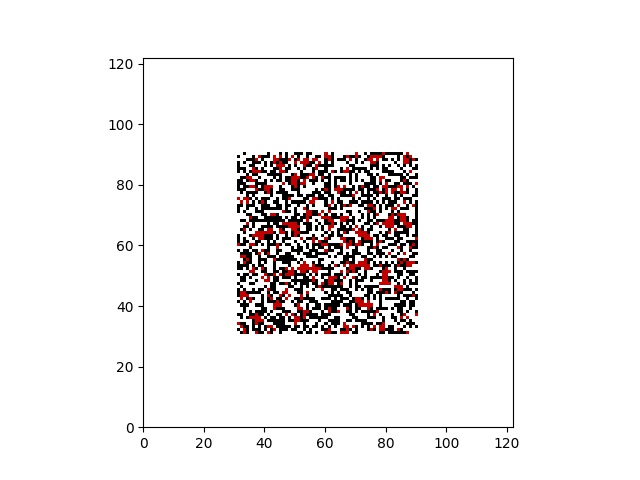

Supplement: Supplementary file 10 — Supplementary Software 1 [file 41467_2021_21614_MOESM10_ESM.zip › SupplementaryCode/DemoResult/Volume_60/seq00049.jpg]

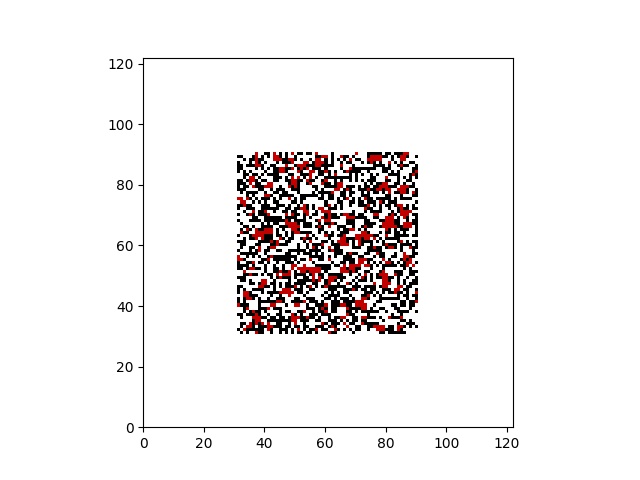

Supplement: Supplementary file 10 — Supplementary Software 1 [file 41467_2021_21614_MOESM10_ESM.zip › SupplementaryCode/DemoResult/Volume_60/seq00061.jpg]

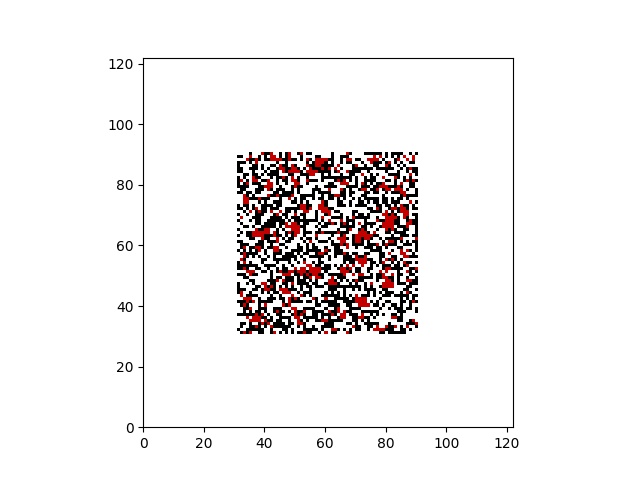

Supplement: Supplementary file 10 — Supplementary Software 1 [file 41467_2021_21614_MOESM10_ESM.zip › SupplementaryCode/DemoResult/Volume_60/seq00075.jpg]

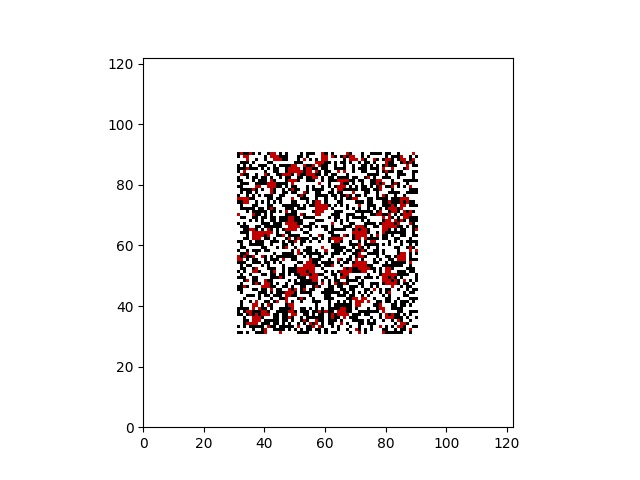

Supplement: Supplementary file 10 — Supplementary Software 1 [file 41467_2021_21614_MOESM10_ESM.zip › SupplementaryCode/DemoResult/Volume_60/seq00129.jpg]

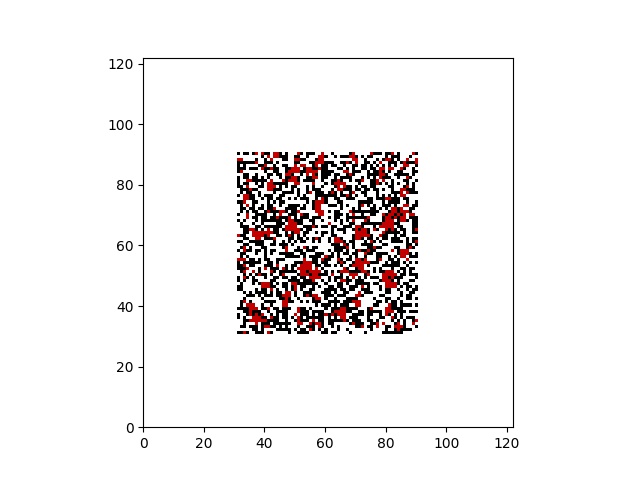

Supplement: Supplementary file 10 — Supplementary Software 1 [file 41467_2021_21614_MOESM10_ESM.zip › SupplementaryCode/DemoResult/Volume_60/seq00115.jpg]

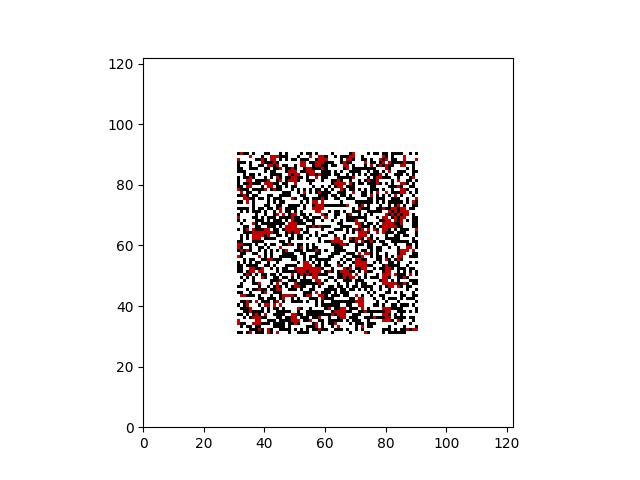

Supplement: Supplementary file 10 — Supplementary Software 1 [file 41467_2021_21614_MOESM10_ESM.zip › SupplementaryCode/DemoResult/Volume_60/seq00101.jpg]

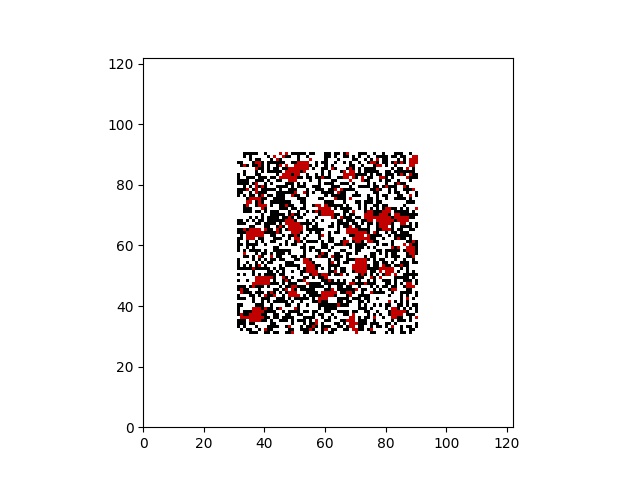

Supplement: Supplementary file 10 — Supplementary Software 1 [file 41467_2021_21614_MOESM10_ESM.zip › SupplementaryCode/DemoResult/Volume_60/seq00459.jpg]

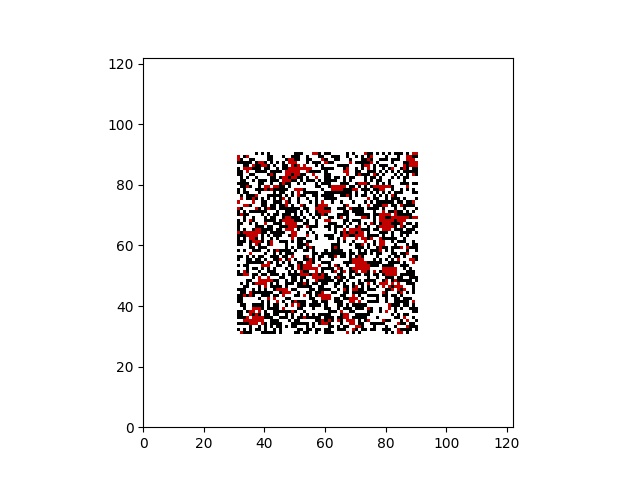

Supplement: Supplementary file 10 — Supplementary Software 1 [file 41467_2021_21614_MOESM10_ESM.zip › SupplementaryCode/DemoResult/Volume_60/seq00317.jpg]

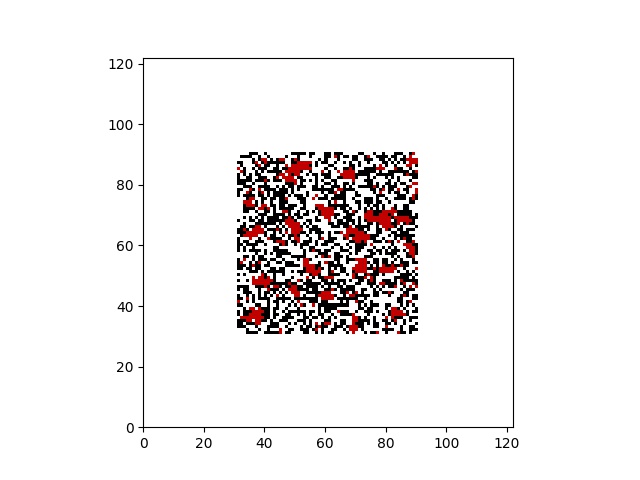

Supplement: Supplementary file 10 — Supplementary Software 1 [file 41467_2021_21614_MOESM10_ESM.zip › SupplementaryCode/DemoResult/Volume_60/seq00471.jpg]

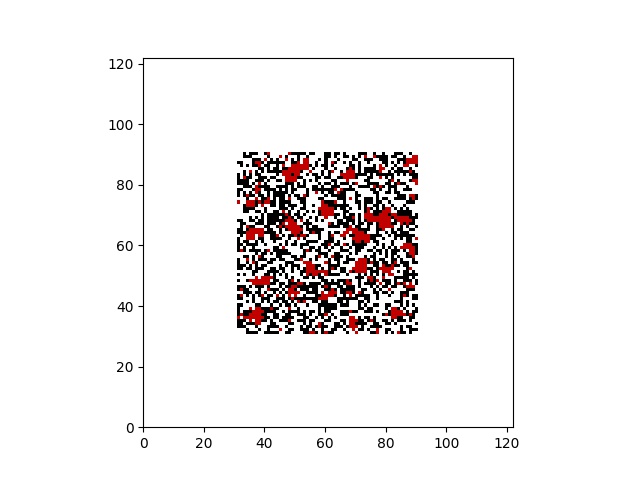

Supplement: Supplementary file 10 — Supplementary Software 1 [file 41467_2021_21614_MOESM10_ESM.zip › SupplementaryCode/DemoResult/Volume_60/seq00465.jpg]

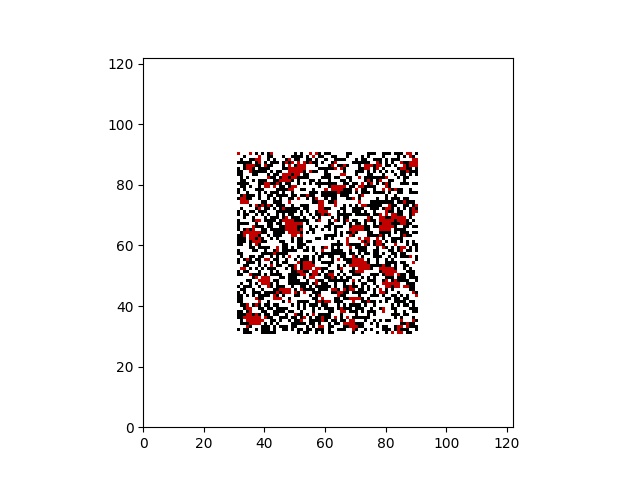

Supplement: Supplementary file 10 — Supplementary Software 1 [file 41467_2021_21614_MOESM10_ESM.zip › SupplementaryCode/DemoResult/Volume_60/seq00303.jpg]

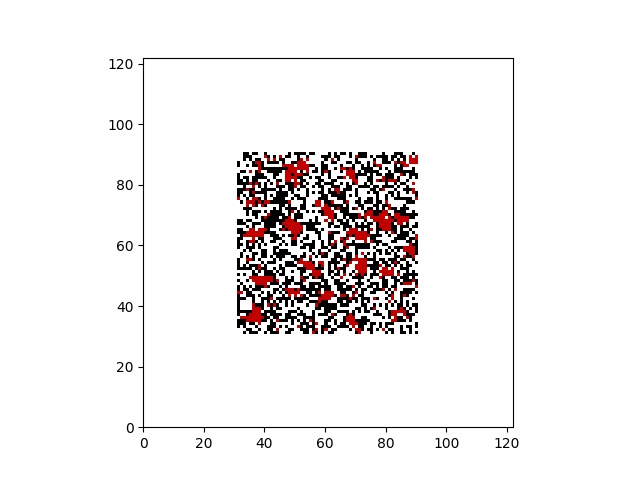

Supplement: Supplementary file 10 — Supplementary Software 1 [file 41467_2021_21614_MOESM10_ESM.zip › SupplementaryCode/DemoResult/Volume_60/seq00449.jpg]

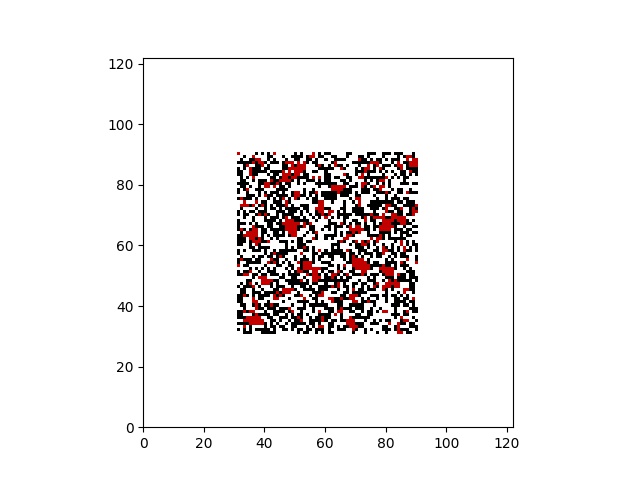

Supplement: Supplementary file 10 — Supplementary Software 1 [file 41467_2021_21614_MOESM10_ESM.zip › SupplementaryCode/DemoResult/Volume_60/seq00307.jpg]

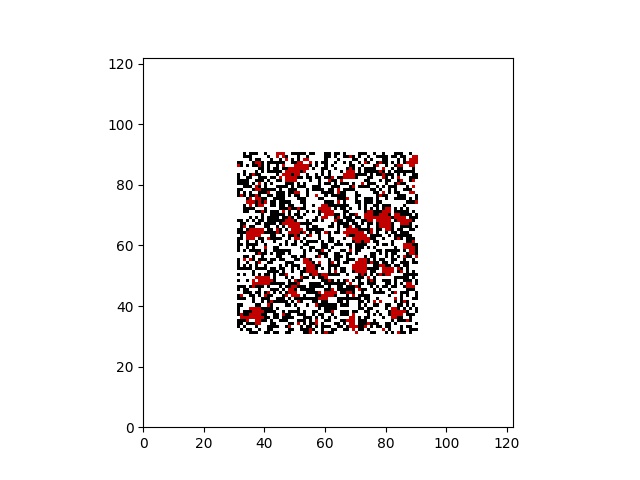

Supplement: Supplementary file 10 — Supplementary Software 1 [file 41467_2021_21614_MOESM10_ESM.zip › SupplementaryCode/DemoResult/Volume_60/seq00461.jpg]

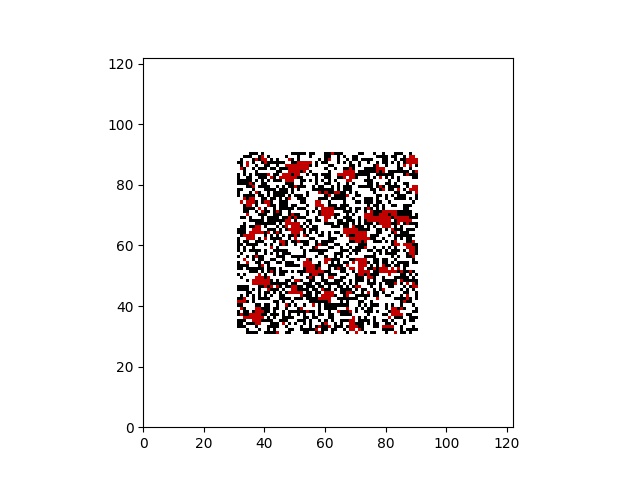

Supplement: Supplementary file 10 — Supplementary Software 1 [file 41467_2021_21614_MOESM10_ESM.zip › SupplementaryCode/DemoResult/Volume_60/seq00475.jpg]

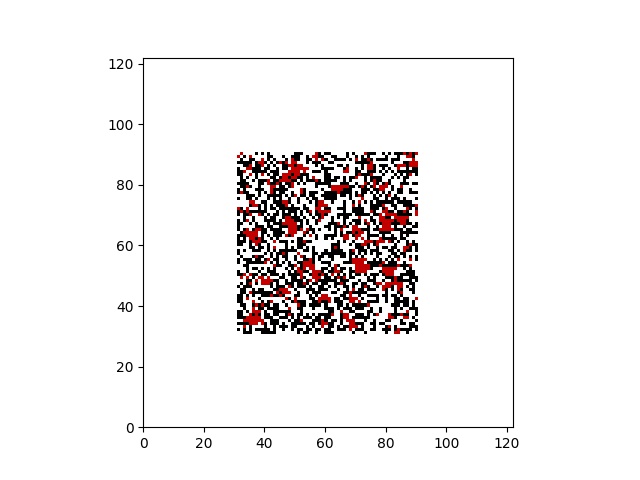

Supplement: Supplementary file 10 — Supplementary Software 1 [file 41467_2021_21614_MOESM10_ESM.zip › SupplementaryCode/DemoResult/Volume_60/seq00313.jpg]

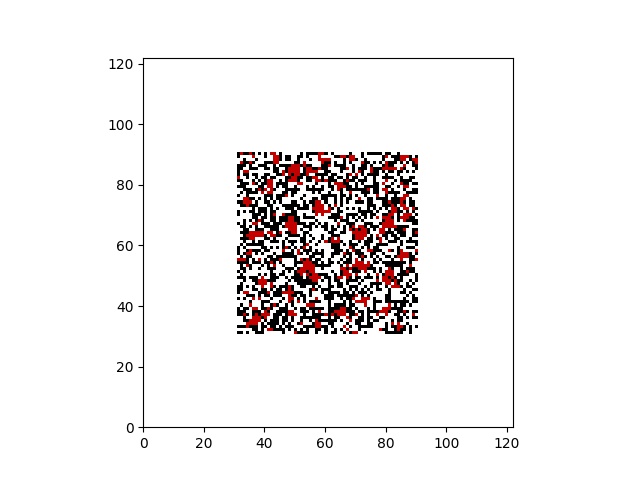

Supplement: Supplementary file 10 — Supplementary Software 1 [file 41467_2021_21614_MOESM10_ESM.zip › SupplementaryCode/DemoResult/Volume_60/seq00139.jpg]

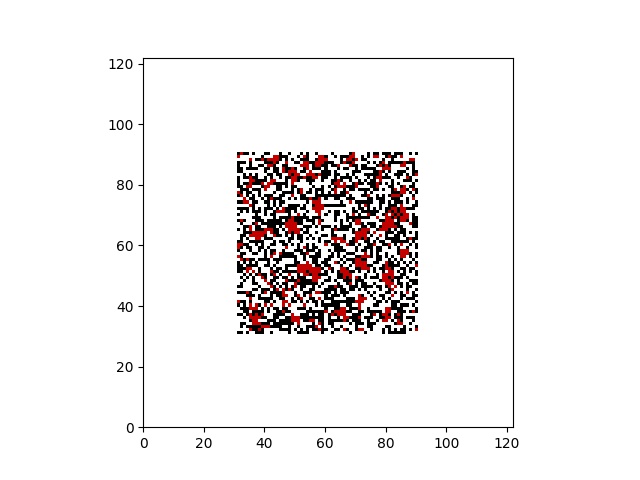

Supplement: Supplementary file 10 — Supplementary Software 1 [file 41467_2021_21614_MOESM10_ESM.zip › SupplementaryCode/DemoResult/Volume_60/seq00105.jpg]

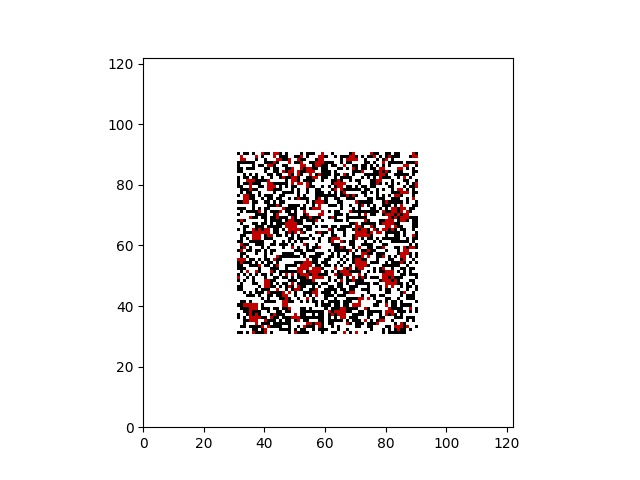

Supplement: Supplementary file 10 — Supplementary Software 1 [file 41467_2021_21614_MOESM10_ESM.zip › SupplementaryCode/DemoResult/Volume_60/seq00111.jpg]

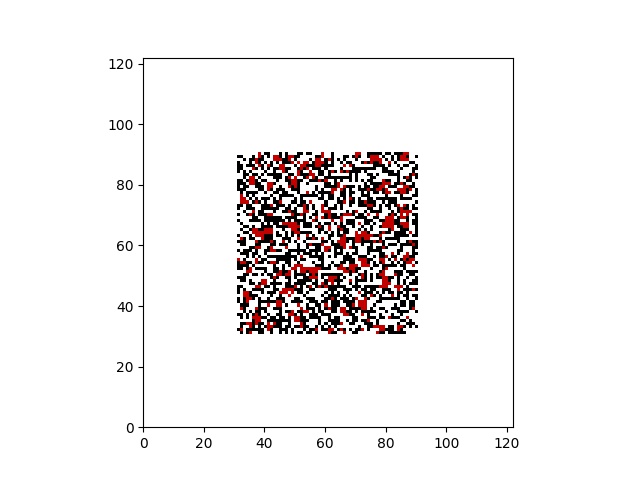

Supplement: Supplementary file 10 — Supplementary Software 1 [file 41467_2021_21614_MOESM10_ESM.zip › SupplementaryCode/DemoResult/Volume_60/seq00059.jpg]

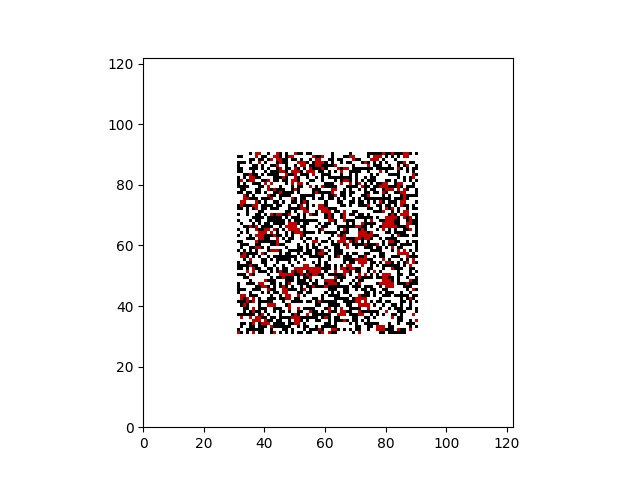

Supplement: Supplementary file 10 — Supplementary Software 1 [file 41467_2021_21614_MOESM10_ESM.zip › SupplementaryCode/DemoResult/Volume_60/seq00071.jpg]

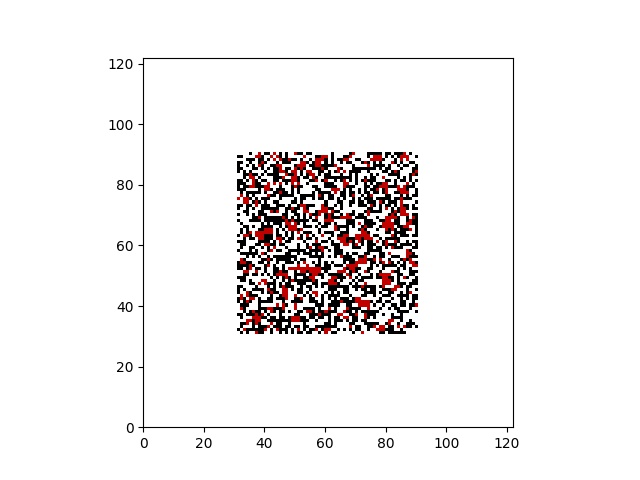

Supplement: Supplementary file 10 — Supplementary Software 1 [file 41467_2021_21614_MOESM10_ESM.zip › SupplementaryCode/DemoResult/Volume_60/seq00065.jpg]

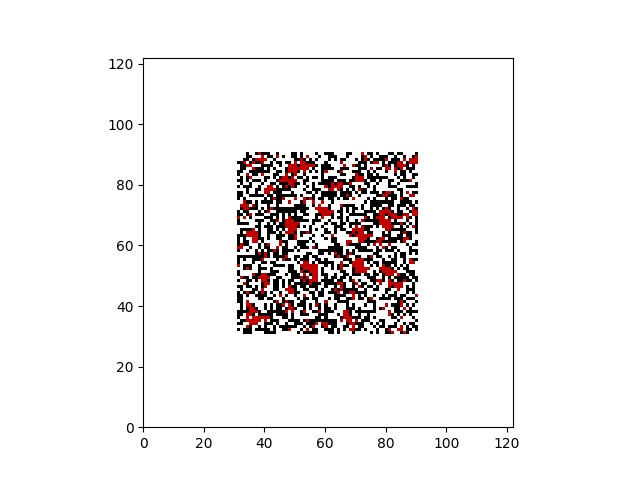

Supplement: Supplementary file 10 — Supplementary Software 1 [file 41467_2021_21614_MOESM10_ESM.zip › SupplementaryCode/DemoResult/Volume_60/seq00273.jpg]

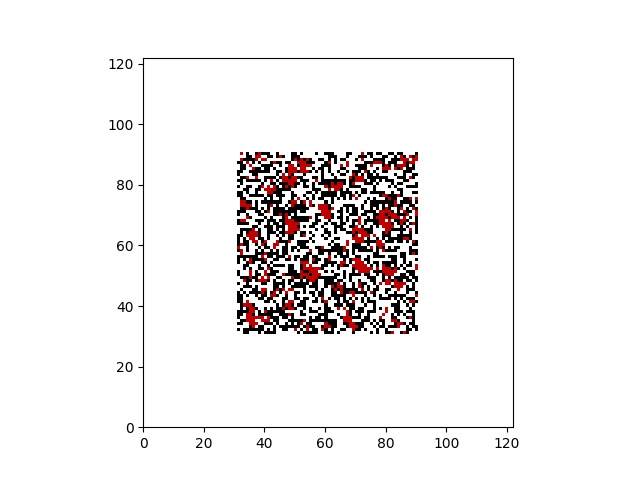

Supplement: Supplementary file 10 — Supplementary Software 1 [file 41467_2021_21614_MOESM10_ESM.zip › SupplementaryCode/DemoResult/Volume_60/seq00267.jpg]

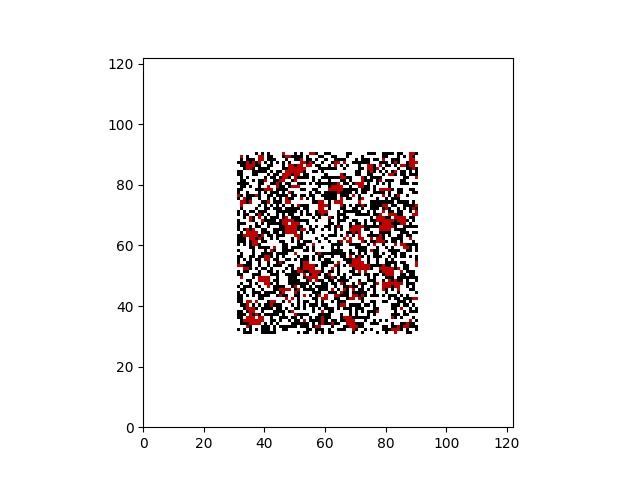

Supplement: Supplementary file 10 — Supplementary Software 1 [file 41467_2021_21614_MOESM10_ESM.zip › SupplementaryCode/DemoResult/Volume_60/seq00298.jpg]

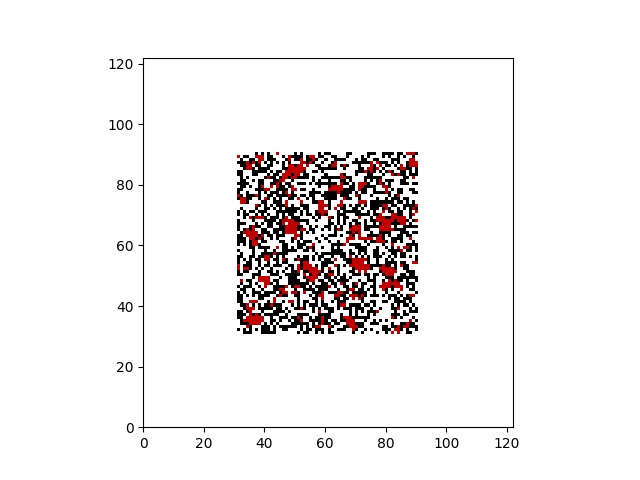

Supplement: Supplementary file 10 — Supplementary Software 1 [file 41467_2021_21614_MOESM10_ESM.zip › SupplementaryCode/DemoResult/Volume_60/seq00299.jpg]

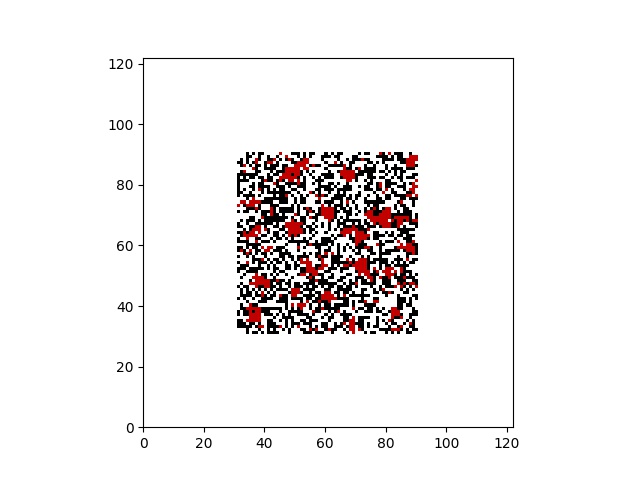

Supplement: Supplementary file 10 — Supplementary Software 1 [file 41467_2021_21614_MOESM10_ESM.zip › SupplementaryCode/DemoResult/Volume_60/seq00500.jpg]

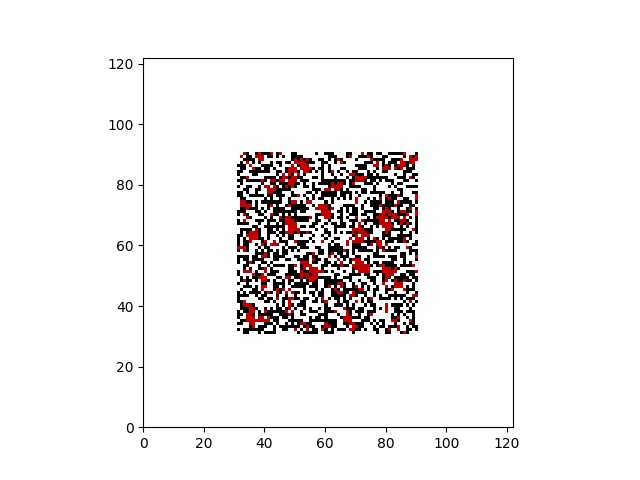

Supplement: Supplementary file 10 — Supplementary Software 1 [file 41467_2021_21614_MOESM10_ESM.zip › SupplementaryCode/DemoResult/Volume_60/seq00266.jpg]

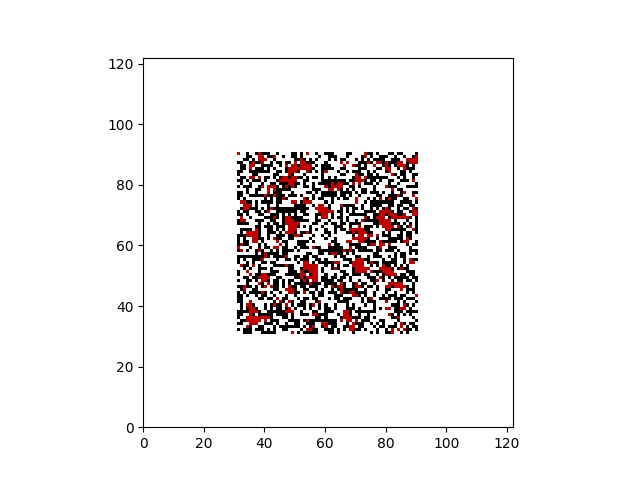

Supplement: Supplementary file 10 — Supplementary Software 1 [file 41467_2021_21614_MOESM10_ESM.zip › SupplementaryCode/DemoResult/Volume_60/seq00272.jpg]

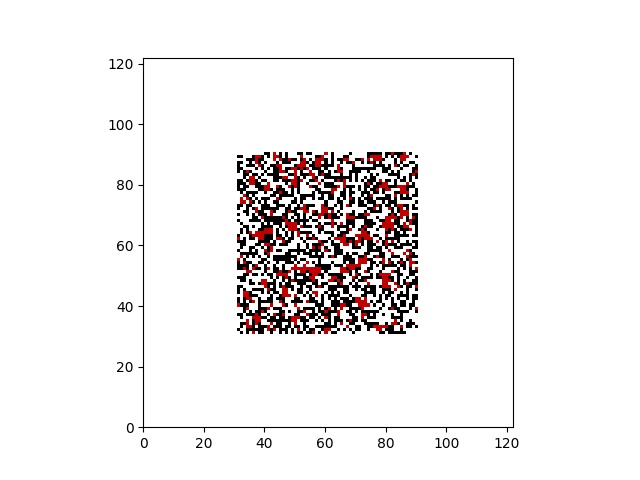

Supplement: Supplementary file 10 — Supplementary Software 1 [file 41467_2021_21614_MOESM10_ESM.zip › SupplementaryCode/DemoResult/Volume_60/seq00064.jpg]

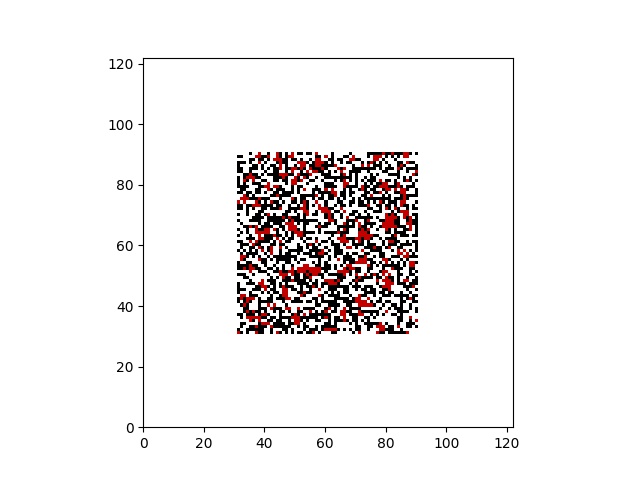

Supplement: Supplementary file 10 — Supplementary Software 1 [file 41467_2021_21614_MOESM10_ESM.zip › SupplementaryCode/DemoResult/Volume_60/seq00070.jpg]

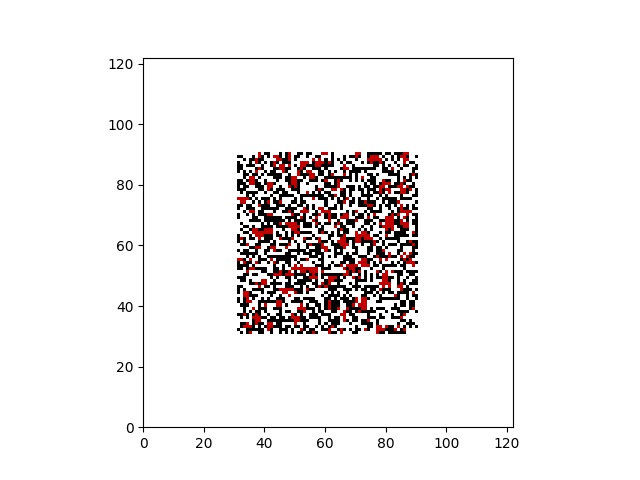

Supplement: Supplementary file 10 — Supplementary Software 1 [file 41467_2021_21614_MOESM10_ESM.zip › SupplementaryCode/DemoResult/Volume_60/seq00058.jpg]

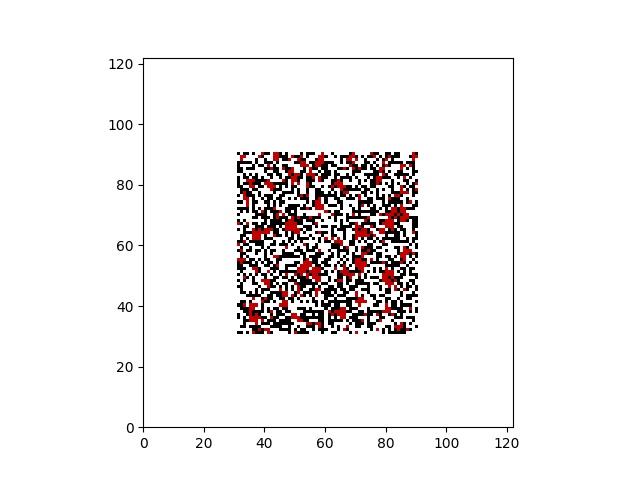

Supplement: Supplementary file 10 — Supplementary Software 1 [file 41467_2021_21614_MOESM10_ESM.zip › SupplementaryCode/DemoResult/Volume_60/seq00110.jpg]

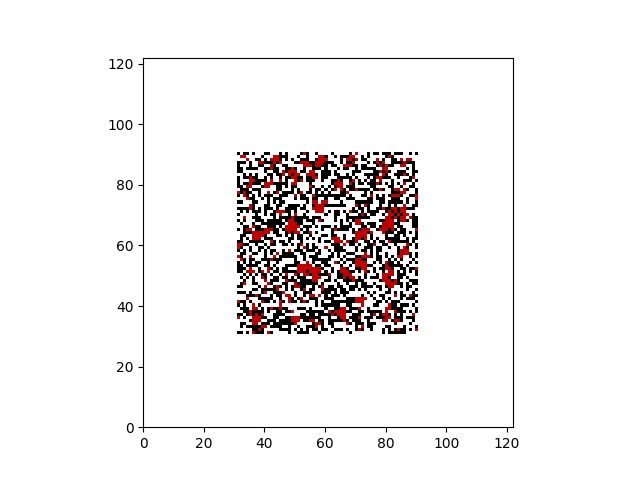

Supplement: Supplementary file 10 — Supplementary Software 1 [file 41467_2021_21614_MOESM10_ESM.zip › SupplementaryCode/DemoResult/Volume_60/seq00104.jpg]

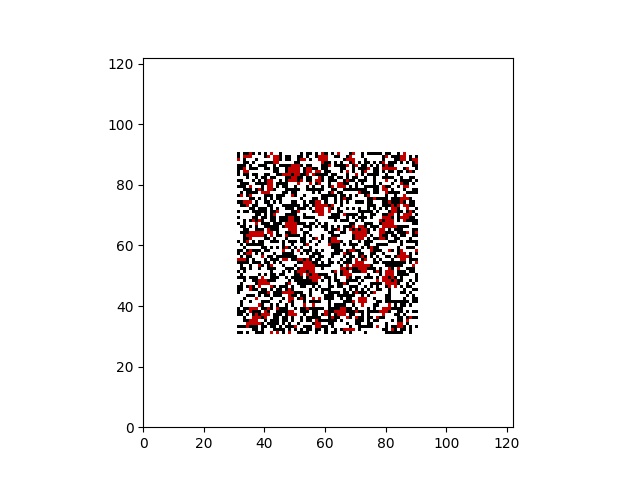

Supplement: Supplementary file 10 — Supplementary Software 1 [file 41467_2021_21614_MOESM10_ESM.zip › SupplementaryCode/DemoResult/Volume_60/seq00138.jpg]

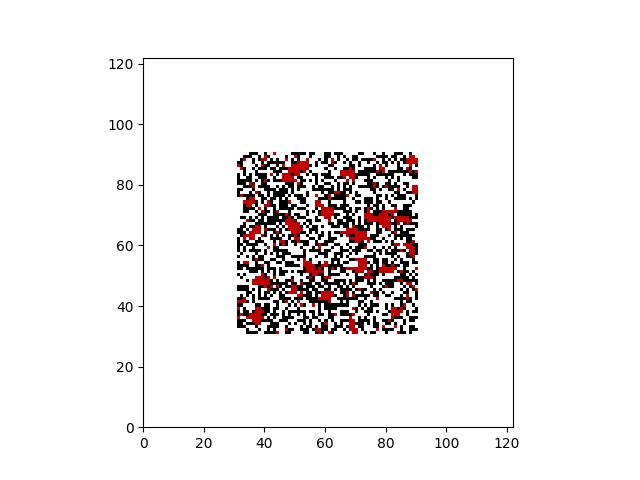

Supplement: Supplementary file 10 — Supplementary Software 1 [file 41467_2021_21614_MOESM10_ESM.zip › SupplementaryCode/DemoResult/Volume_60/seq00474.jpg]

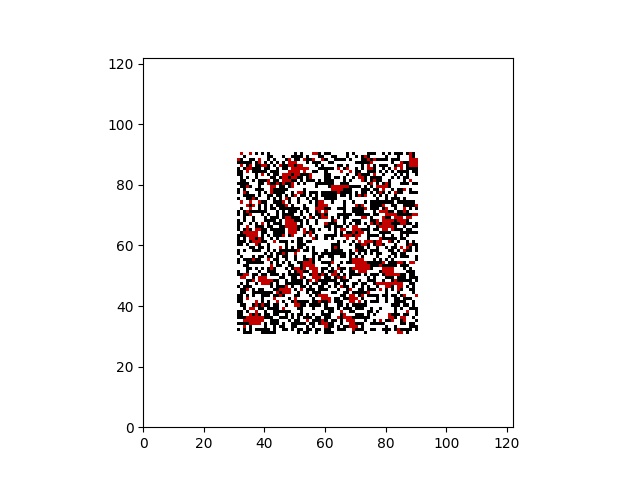

Supplement: Supplementary file 10 — Supplementary Software 1 [file 41467_2021_21614_MOESM10_ESM.zip › SupplementaryCode/DemoResult/Volume_60/seq00312.jpg]

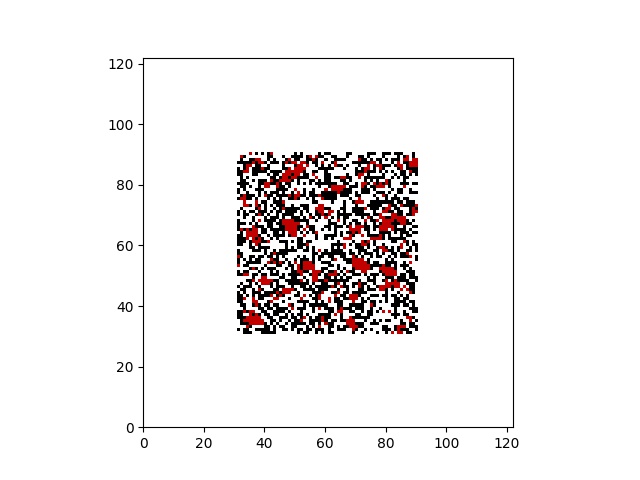

Supplement: Supplementary file 10 — Supplementary Software 1 [file 41467_2021_21614_MOESM10_ESM.zip › SupplementaryCode/DemoResult/Volume_60/seq00306.jpg]

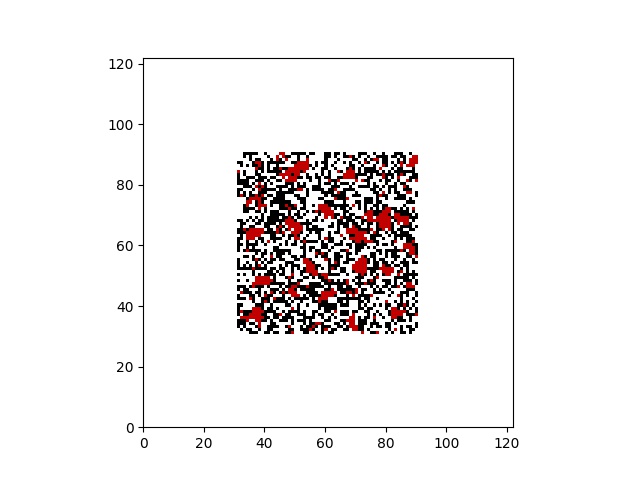

Supplement: Supplementary file 10 — Supplementary Software 1 [file 41467_2021_21614_MOESM10_ESM.zip › SupplementaryCode/DemoResult/Volume_60/seq00460.jpg]

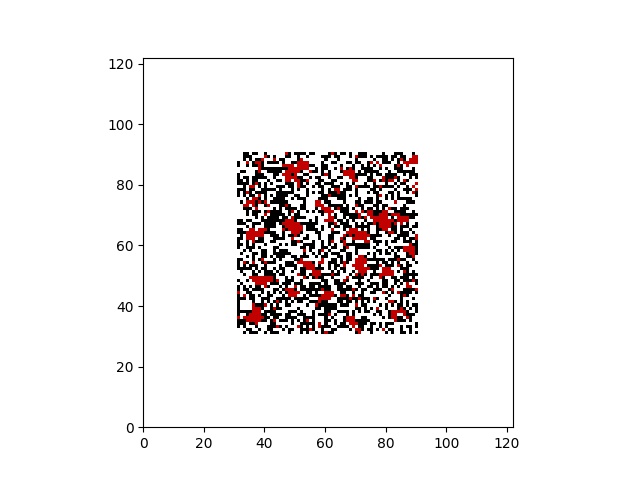

Supplement: Supplementary file 10 — Supplementary Software 1 [file 41467_2021_21614_MOESM10_ESM.zip › SupplementaryCode/DemoResult/Volume_60/seq00448.jpg]

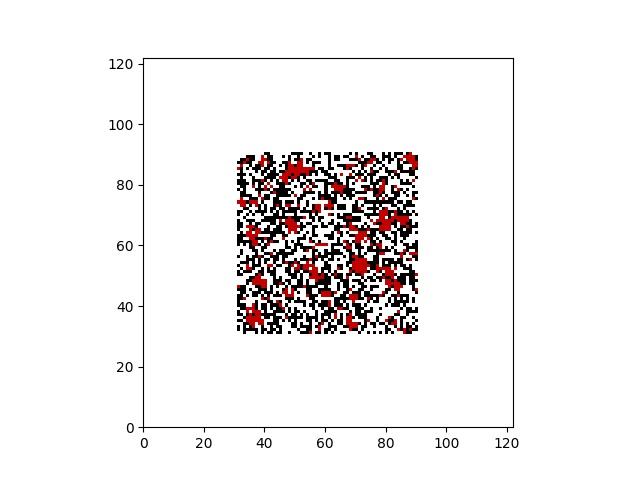

Supplement: Supplementary file 10 — Supplementary Software 1 [file 41467_2021_21614_MOESM10_ESM.zip › SupplementaryCode/DemoResult/Volume_60/seq00338.jpg]

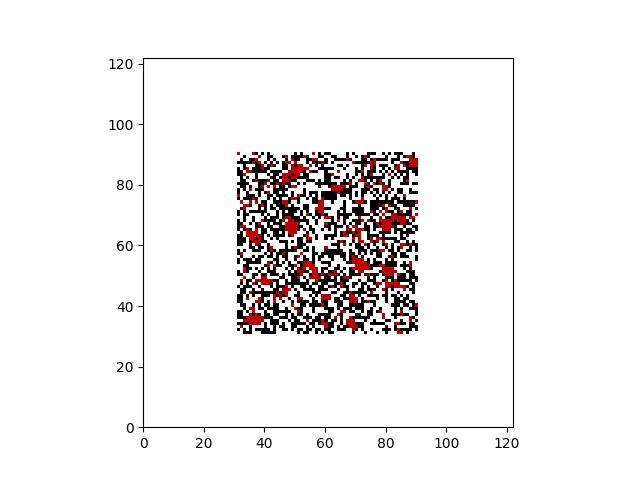

Supplement: Supplementary file 10 — Supplementary Software 1 [file 41467_2021_21614_MOESM10_ESM.zip › SupplementaryCode/DemoResult/Volume_60/seq00310.jpg]

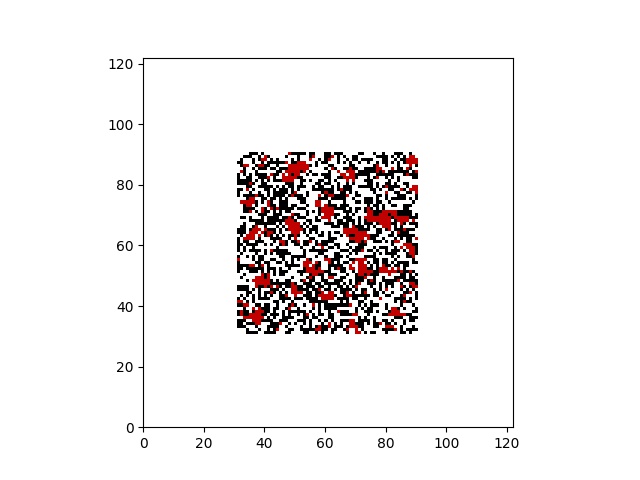

Supplement: Supplementary file 10 — Supplementary Software 1 [file 41467_2021_21614_MOESM10_ESM.zip › SupplementaryCode/DemoResult/Volume_60/seq00476.jpg]

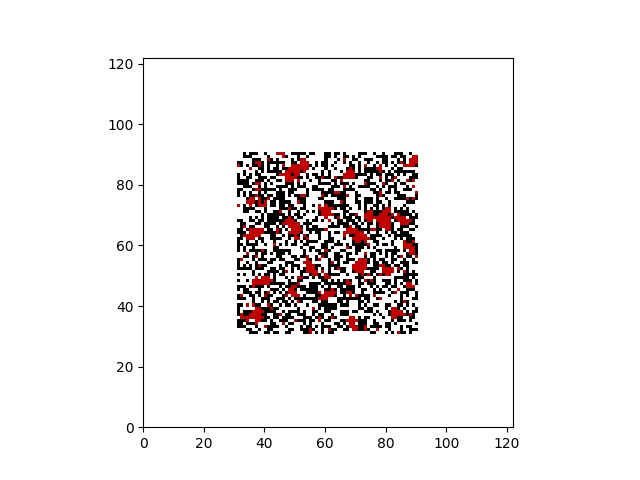

Supplement: Supplementary file 10 — Supplementary Software 1 [file 41467_2021_21614_MOESM10_ESM.zip › SupplementaryCode/DemoResult/Volume_60/seq00462.jpg]

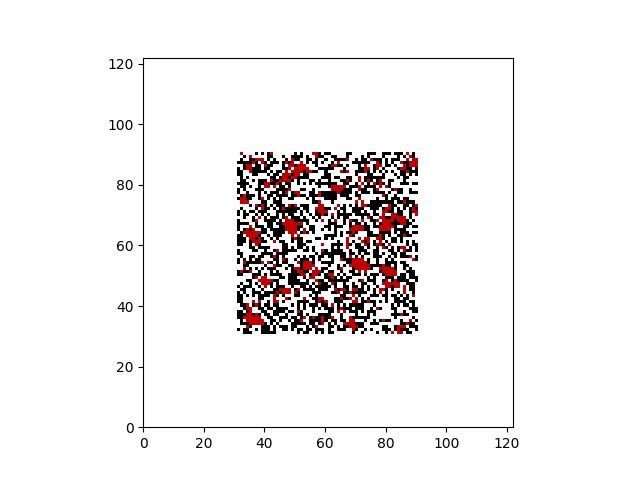

Supplement: Supplementary file 10 — Supplementary Software 1 [file 41467_2021_21614_MOESM10_ESM.zip › SupplementaryCode/DemoResult/Volume_60/seq00304.jpg]

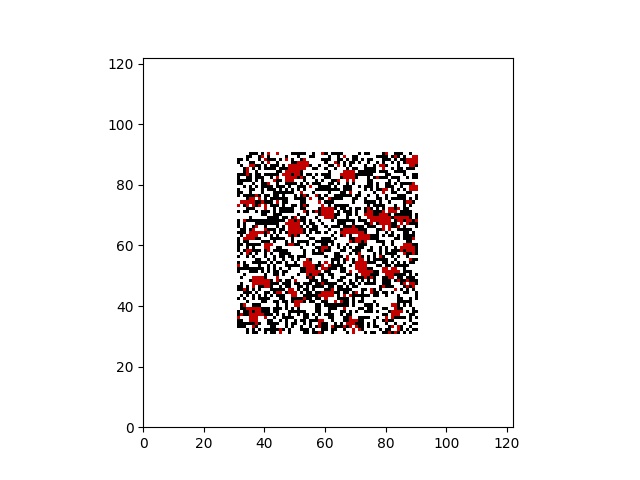

Supplement: Supplementary file 10 — Supplementary Software 1 [file 41467_2021_21614_MOESM10_ESM.zip › SupplementaryCode/DemoResult/Volume_60/seq00489.jpg]

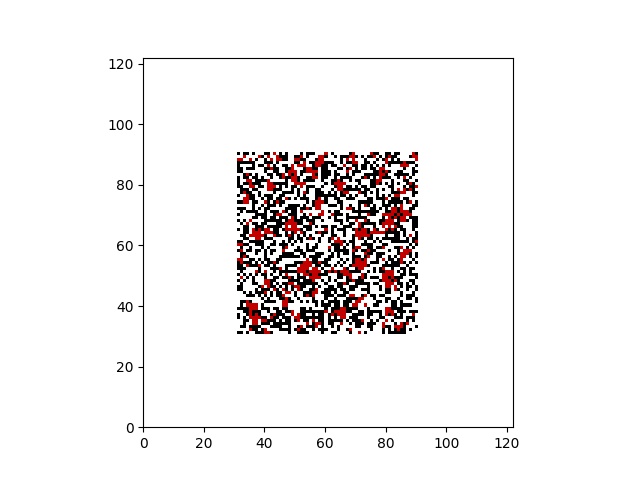

Supplement: Supplementary file 10 — Supplementary Software 1 [file 41467_2021_21614_MOESM10_ESM.zip › SupplementaryCode/DemoResult/Volume_60/seq00112.jpg]

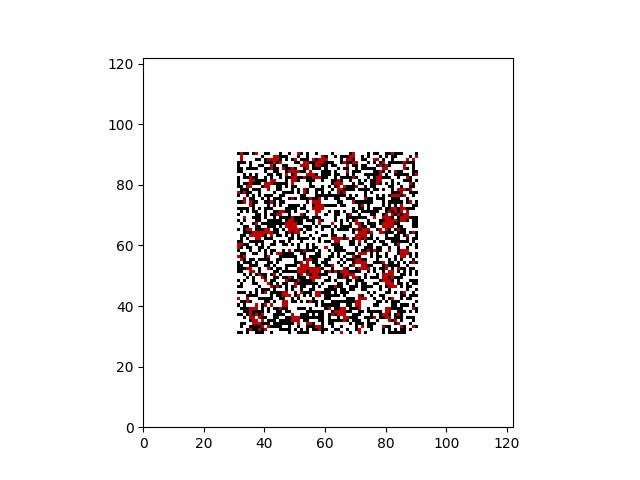

Supplement: Supplementary file 10 — Supplementary Software 1 [file 41467_2021_21614_MOESM10_ESM.zip › SupplementaryCode/DemoResult/Volume_60/seq00106.jpg]

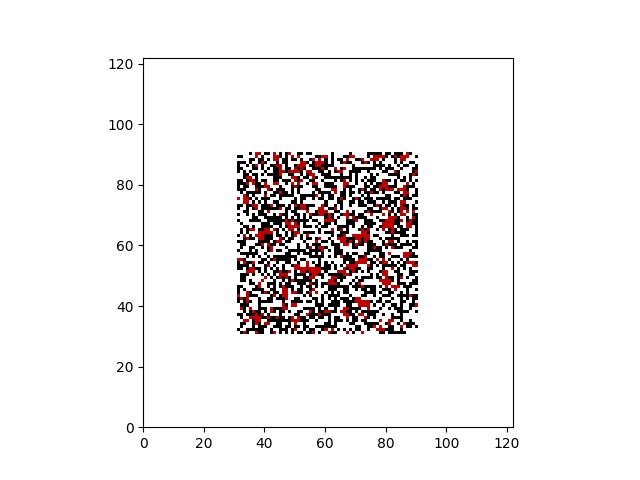

Supplement: Supplementary file 10 — Supplementary Software 1 [file 41467_2021_21614_MOESM10_ESM.zip › SupplementaryCode/DemoResult/Volume_60/seq00066.jpg]

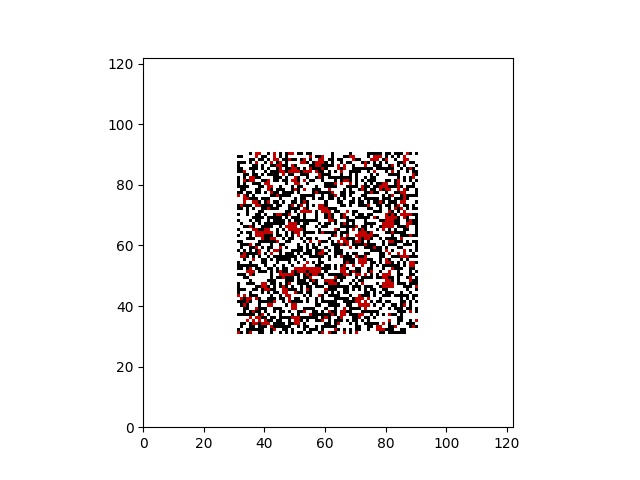

Supplement: Supplementary file 10 — Supplementary Software 1 [file 41467_2021_21614_MOESM10_ESM.zip › SupplementaryCode/DemoResult/Volume_60/seq00072.jpg]

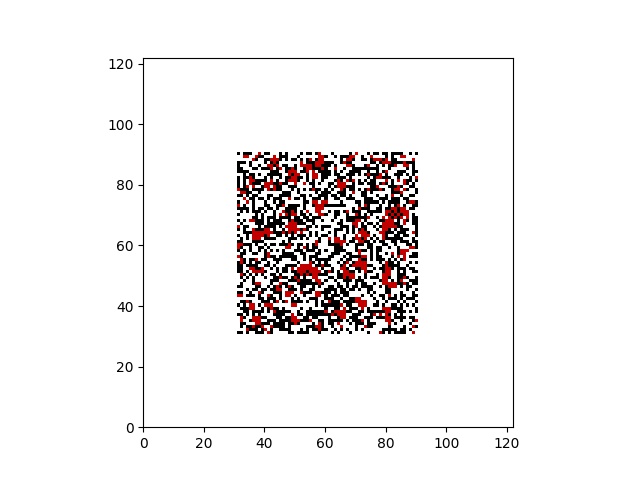

Supplement: Supplementary file 10 — Supplementary Software 1 [file 41467_2021_21614_MOESM10_ESM.zip › SupplementaryCode/DemoResult/Volume_60/seq00099.jpg]

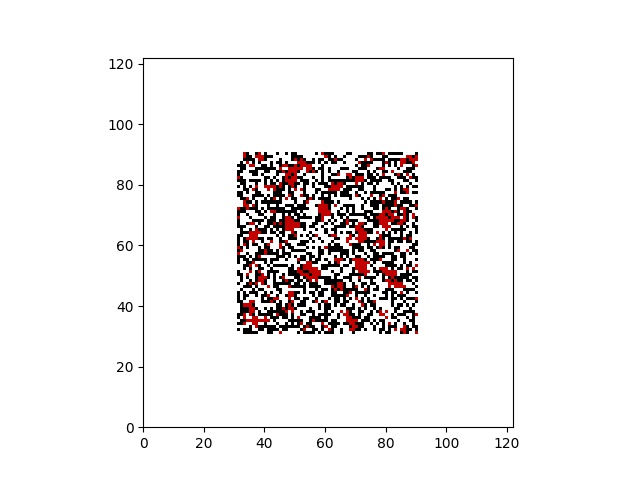

Supplement: Supplementary file 10 — Supplementary Software 1 [file 41467_2021_21614_MOESM10_ESM.zip › SupplementaryCode/DemoResult/Volume_60/seq00258.jpg]

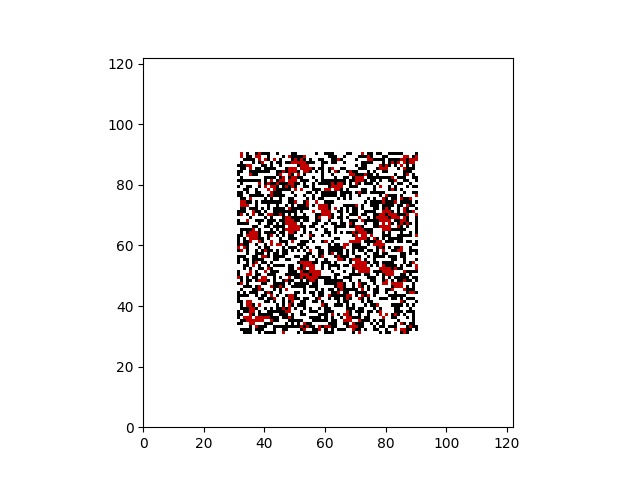

Supplement: Supplementary file 10 — Supplementary Software 1 [file 41467_2021_21614_MOESM10_ESM.zip › SupplementaryCode/DemoResult/Volume_60/seq00264.jpg]

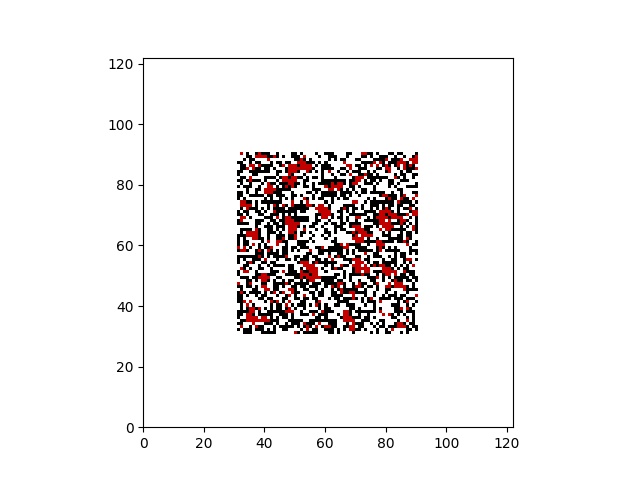

Supplement: Supplementary file 10 — Supplementary Software 1 [file 41467_2021_21614_MOESM10_ESM.zip › SupplementaryCode/DemoResult/Volume_60/seq00270.jpg]

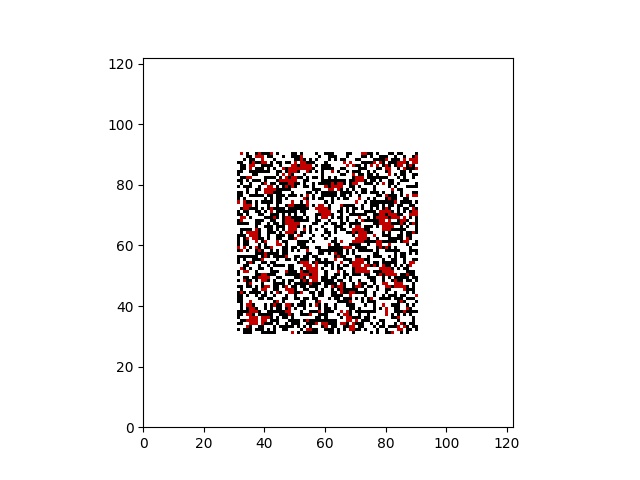

Supplement: Supplementary file 10 — Supplementary Software 1 [file 41467_2021_21614_MOESM10_ESM.zip › SupplementaryCode/DemoResult/Volume_60/seq00271.jpg]
