# Supplementary material for: Cells recognize osmotic stress through liquid–liquid phase separation lubricated with poly(ADP-ribose)
Source: Nat Commun. 2021 Mar 1;12:1353. doi: 10.1038/s41467-021-21614-5 (PMC7921423; doi:10.1038/s41467-021-21614-5)
Supplement: Supplementary file 11 — Reporting Summary [file 41467_2021_21614_MOESM11_ESM.pdf]

nature research

Corresponding author(s): Kengo Watanabe and Hidenori Ichijo

Last updated by author(s): Jan 11, 2021

Reporting Summary

Nature Research wishes to improve the reproducibility of the work that we publish. This form provides structure for consistency and transparency in reporting. For further information on Nature Research policies, see our Editorial Policies and the Editorial Policy Checklist.

Statistics

For all statistical analyses, confirm that the following items are present in the figure legend, table legend, main text, or Methods section.

n/a	Confirmed	
☐	☒	The exact sample size (*n*) for each experimental group/condition, given as a discrete number and unit of measurement
☐	☒	A statement on whether measurements were taken from distinct samples or whether the same sample was measured repeatedly
☐	☒	The statistical test(s) used AND whether they are one- or two-sided<br>*Only common tests should be described solely by name; describe more complex techniques in the Methods section.*
☒	☐	A description of all covariates tested
☐	☒	A description of any assumptions or corrections, such as tests of normality and adjustment for multiple comparisons
☒	☐	A full description of the statistical parameters including central tendency (e.g. means) or other basic estimates (e.g. regression coefficient) AND variation (e.g. standard deviation) or associated estimates of uncertainty (e.g. confidence intervals)
☐	☒	For null hypothesis testing, the test statistic (e.g. *F*, *t*, *r*) with confidence intervals, effect sizes, degrees of freedom and *P* value noted<br>*Give P values as exact values whenever suitable.*
☒	☐	For Bayesian analysis, information on the choice of priors and Markov chain Monte Carlo settings
☒	☐	For hierarchical and complex designs, identification of the appropriate level for tests and full reporting of outcomes
☒	☐	Estimates of effect sizes (e.g. Cohen's *d*, Pearson's *r*), indicating how they were calculated

Our web collection on statistics for biologists contains articles on many of the points above.

Software and code

Policy information about availability of computer code

Data collection	As summarized in Supplementary Data 1, Python (ver. 3.6.5) and its libraries NumPy (ver. 1.14.5), pandas (ver. 0.23.3) and Matplotlib (ver. 3.0.2) were used in computational simulations. Python scripts for the computational simulations are provided in Supplementary Software.
Data analysis	As described in the Methods section and summarized in Supplementary Data 1, Fiji/ImageJ (ver. 2.0.0) and its plugin TrackMate (ver. 4.0.1) were used in image data analyses; R (ver. 3.6.0) and its packages tidyverse (ver. 1.2.1) and multcomp (ver. 1.4.10) were used in numerical data and statistical analyses; IUPred2A (URL https://iupred2a.elte.hu/) was used in the prediction of intrinsically disordered regions; GNU Image Manipulation Program (GIMP; ver. 2.8.22), Fiji/ImageJ and R with its packages tidyverse, ggpubr (ver. 0.2.4), RColorBrewer (ver. 1.1.2) and lemon (ver. 0.4.3) were used in data visualizations. All other custom scripts used in this study are fundamental enough for standard users to code the procedures described in the Methods section, but further information and requests for the custom scripts should be directed to K.W. and H.I.

For manuscripts utilizing custom algorithms or software that are central to the research but not yet described in published literature, software must be made available to editors and reviewers. We strongly encourage code deposition in a community repository (e.g. GitHub). See the Nature Research guidelines for submitting code & software for further information.

Data

Policy information about availability of data

All manuscripts must include a data availability statement. This statement should provide the following information, where applicable:

- Accession codes, unique identifiers, or web links for publicly available datasets
- A list of figures that have associated raw data
- A description of any restrictions on data availability

The authors declare that all data supporting the findings of this study are available within the paper and its Supplementary Information files. Source Data are

provided with this paper. Further information and requests for resources and reagents should be directed to K.W. and H.I.

Field-specific reporting

Please select the one below that is the best fit for your research. If you are not sure, read the appropriate sections before making your selection.

- [x] Life sciences
- [ ] Behavioural & social sciences
- [ ] Ecological, evolutionary & environmental sciences

For a reference copy of the document with all sections, see nature.com/documents/nr-reporting-summary-flat.pdf

Life sciences study design

All studies must disclose on these points even when the disclosure is negative.

Sample size	No statistical method was utilized to predetermine the sample size because all experiments in this study were performed with defined laboratory reagents and cell lines. Based on several pilot experiments to determine the experimental conditions, each sample size was chosen as large as possible to represent experimental variation while still practically feasible in terms of data collection.
Data exclusions	In live-cell imaging and FRAP assays, we excluded several data points and samples in data analysis because they were reasonably regarded as poor quality data or extreme abnormal outliers. The exclusion criteria are clearly described in the Methods section.
Replication	All findings were reproduced more than three times through the independent experiments, including pilot experiments to determine the experimental conditions and experiments used in null hypothesis testing.
Randomization	The independent experiments were performed across different passages of cells, and the cells in the control and treated groups were seeded from the same population of cells.
Blinding	The investigators were not blinded to allocation during experiments and outcome assessments. Nevertheless, the assessments were semi-automated with macro scripts in Fiji/ImageJ and R scripts, and there was little room for subjective bias.

Reporting for specific materials, systems and methods

We require information from authors about some types of materials, experimental systems and methods used in many studies. Here, indicate whether each material, system or method listed is relevant to your study. If you are not sure if a list item applies to your research, read the appropriate section before selecting a response.

Materials & experimental systems

n/a	Involved in the study	
☐	☒	Antibodies
☐	☒	Eukaryotic cell lines
☒	☐	Palaeontology and archaeology
☒	☐	Animals and other organisms
☒	☐	Human research participants
☒	☐	Clinical data
☒	☐	Dual use research of concern

Methods

n/a	Involved in the study	
☒	☐	ChIP-seq
☒	☐	Flow cytometry
☒	☐	MRI-based neuroimaging

Antibodies

Antibodies used	As described in the Methods section and summarized in Supplementary Data 1, rabbit polyclonal anti-phospho-ASK (p-ASK; Thr808 in human ASK3; Naguro, I. et al. Nat. Commun. 2012; Tobiume, K. et al. J. Cell Physiol. 2002), anti-rabbit IgG, HRP-linked (Cell Signaling Technology, Cat. #7074), mouse monoclonal anti-DYKDDDDK tag (FLAG; clone 1E6; Wako Pure Chemical Industries, Cat. #012-22384), anti-mouse IgG, HRP-linked (Cell Signaling Technology, Cat. #7076), mouse monoclonal anti-Actin (Actin; clone AC-40; Sigma-Aldrich, Cat. #A3853), rabbit polyclonal anti-PBEF (PBEF; Bethyl Laboratories, Cat. #A300-372A), rat monoclonal anti-ASK3 (ASK3; Naguro, I. et al. Nat. Commun. 2012), anti-rat IgG, HRP-linked (Cell Signaling Technology, Cat. #7077), rabbit polyclonal anti-phospho-SPAK/OSR1 (p-SPAK/OSR1; Thr231 in human SPAK and Thr185 in human OSR1; Naguro, I. et al. Nat. Commun. 2012), mouse monoclonal anti-OXSR1 (OSR1; clone 2A2-1A2; Abnova, Cat. #H00009943-M01), mouse monoclonal anti-GFP (GFP; clone 1E4; Medical & Biological Laboratories, Cat. #M048-3), rat monoclonal anti-YPYDVPDYA tag (HA; clone 3F10; Roche Diagnostics, Cat. #11867431001), rabbit polyclonal anti-Poly(ADP-ribose) (PAR; Enzo Life Sciences, Cat. #ALX-210-890A), anti-DYKDDDDK tag Antibody Beads (FLAG; clone 1E6; Wako Pure Chemical Industries, Cat. #016-22784) and ANTI-FLAG M2 Affinity Gel (clone M2; Sigma-Aldrich, Cat. #A2220) were used in immunoblotting and coimmunoprecipitation; mouse monoclonal anti-EEA1 (EEA1; clone 14/EEA1; BD Transduction Laboratories, Cat. #610456); Alexa Fluor 555 goat anti-mouse IgG (H+L) (Molecular Probe, Cat. #A21422), mouse monoclonal anti-LAMP1 (LAMP1; clone H4A3; Santa Cruz Biotechnology, Cat. #sc-20011) and Alexa Fluor 633 goat anti-rat IgG (H+L) (Molecular Probe, Cat. #A21094) were used in immunofluorescence; rabbit polyclonal anti-GFP antibody (Frontier Institute (Cat. #GFP-Rb-Af2020) was used in immunogold labelling.
Validation	The specificity of commercially available antibodies were validated according to manufactures' datasheets. In addition, the specificity

of anti-PBEF antibody was also clearly validated in Fig. 4c, 4d, 5b, and the specificity of anti-PAR antibody was well characterized in Fig. 5b, 5c, 6d. The lab-made antibodies (p-ASK, ASK3 and p-SPAK/OSR1) were characterized in our previous report (Naguro, I. et al. Nat. Commun. 2012).

Eukaryotic cell lines

Policy information about cell lines

Cell line source(s)	HEK293A cells were purchased from Invitrogen. Tetracycline-inducible Venus-ASK3-stably expressing HEK293A (Venus-ASK3-HEK293A) cells were established in this study. Tetracycline-inducible FLAG-ASK3-stably expressing HEK293A (FLAG-ASK3-HEK293A) cells were established previously (Watanabe, K. et al. Cell Rep. 2018).
Authentication	None of the cell lines used in this study were authenticated.
Mycoplasma contamination	All cells were verified to be negative for mycoplasma.
Commonly misidentified lines (See ICLAC register)	No commonly misidentified cell lines were used in this study.
